# European Society for Organ Transplantation (ESOT) Consensus Statement on the Role of Pancreas Machine Perfusion to Increase the Donor Pool for Beta Cell Replacement Therapy

**DOI:** 10.3389/ti.2023.11374

**Published:** 2023-07-19

**Authors:** Joana Ferrer-Fàbrega, Benoît Mesnard, Franka Messner, Jason B. Doppenberg, Cinthia Drachenberg, Marten A. Engelse, Paul R. V. Johnson, Henri G. D. Leuvenink, Gabriel C. Oniscu, Vassilios Papalois, Rutger J. Ploeg, Trevor W. Reichman, William E Scott, Fabio Vistoli, Thierry Berney, Daniel Jacobs-Tulleneers-Thevissen, Nicos Kessaris, Annemarie Weissenbacher, Ann Etohan Ogbemudia, Steve White, Julien Branchereau

**Affiliations:** ^1^ Hepatobiliopancreatic Surgery and Liver and Pancreatic Transplantation Unit, Department of Surgery, Institute Clínic of Digestive and Metabolic Diseases (ICMDiM), Hospital Clínic, University of Barcelona, Barcelona, Spain; ^2^ Hepatic Oncology Unit, Barcelona Clínic Liver Cancer Group (BCLC), Hospital Clínic, Barcelona, Spain; ^3^ August Pi i Sunyer Biomedical, Research Institute (IDIBAPS), University of Barcelona, Barcelona, Spain; ^4^ Network for Biomedical Research in Hepatic and Digestive Diseases (CIBEREHD), Barcelona, Spain; ^5^ Department of Urology and Transplantation Surgery, Nantes University Hospital, Nantes, France; ^6^ Centre for Research in Transplantation and Translational Immunology, INSERM UMR 1064, ITUN5, Nantes, France; ^7^ Department of Visceral, Transplant and Thoracic Surgery, Medical University of Innsbruck, Innsbruck, Austria; ^8^ Transplantation Center, Leiden University Medical Center, Leiden, Netherlands; ^9^ Department of Pathology, University of Maryland School of Medicine, Baltimore, MD, United States; ^10^ Research Group for Islet Transplantation, Nuffield Department of Surgical Sciences, John Radcliffe Hospital, University of Oxford, Oxford, United Kingdom; ^11^ Department of Surgery, University Medical Center Groningen, Groningen, Netherlands; ^12^ Transplant Division, Department of Clinical Science, Intervention and Technology - CLINTEC, Karolinska Institutet, Stockholm, Sweden; ^13^ Imperial College Renal and Transplant Centre, Hammersmith Hospital, Imperial College Healthcare NHS Trust, London, United Kingdom; ^14^ Oxford Transplant Centre, Nuffield Department of Surgical Sciences, University of Oxford, Oxford, United Kingdom; ^15^ Ajmera Transplant Centre, Toronto General Hospital, University Health Network, Toronto, ON, Canada; ^16^ Translational and Clinical Research Institute, Newcastle University, Newcastle upon Tyne, United Kingdom; ^17^ Division of General Surgery and Transplantation, University of Pisa, Pisa, Italy; ^18^ Division of Transplantation, Department of Surgery, University of Geneva Hospitals, Geneva, Switzerland; ^19^ Diabetes Research Center, Vrije Universiteit Brussel, Brussels, Belgium; ^20^ Department of Surgery, Universitair Ziekenhuis Brussel, Vrije Universiteit Brussel, Brussels, Belgium; ^21^ Department of Nephrology and Transplantation, Guy’s Hospital, London, United Kingdom; ^22^ Department of HPB and Transplant Surgery, NIHR BTRU in Organ Donation and Transplantation, The Freeman Hospital, The University of Newcastle upon Tyne, Newcastle upon Tyne, United Kingdom

**Keywords:** islet transplantation, ischemia-reperfusion injury, machine perfusion, persufflation, whole pancreas transplantation

## Abstract

The advent of Machine Perfusion (MP) as a superior form of preservation and assessment for cold storage of both high-risk kidney’s and the liver presents opportunities in the field of beta-cell replacement. It is yet unknown whether such techniques, when applied to the pancreas, can increase the pool of suitable donor organs as well as ameliorating the effects of ischemia incurred during the retrieval process. Recent experimental models of pancreatic MP appear promising. Applications of MP to the pancreas, needs refinement regarding perfusion protocols and organ viability assessment criteria. To address the “Role of pancreas machine perfusion to increase the donor pool for beta cell replacement,” the European Society for Organ Transplantation (ESOT) assembled a dedicated working group comprising of experts to review literature pertaining to the role of MP as a method of improving donor pancreas quality as well as quantity available for transplant, and to develop guidelines founded on evidence-based reviews in experimental and clinical settings. These were subsequently refined during the Consensus Conference when this took place in Prague.

## Introduction

### Description of Health Problem

An estimated 537 million adults aged 20–79 years worldwide (61 million in Europe) have diabetes, approximately 10% of which have type 1 diabetes. By 2030, 643 million (67 million in Europe), and by 2045, 783 million (69 million in Europe) adults aged 20–79 years are projected to be living with diabetes. Thus, while the world’s population is estimated to grow 20% over this period, the number of those with diabetes is estimated to increase by 46% [1].

Diabetes is a major driver for mortality worldwide and is the leading cause of kidney failure, peripheral vascular disease, and adult-onset blindness. Excluding the mortality risks associated with the COVID-19 pandemic, it is estimated that 6.7 million adults between the age of 20–79 died as a result of diabetes related complications during 2021. When inadequately managed, diabetes significantly elevates the risk of a host of micro- and macro-vascular complications. Because of this, optimizing glycemic control is critical in order to delay and potentially prevent the onset of chronic diabetic complications [2, 3]. Despite the tremendous expenditure in human, material, and financial resources, only about 50% of patients achieve optimal treatment. In selected cases, beta cell replacement, by pancreas or islet transplantation, can provide durable glycemic control and improve survival, therefore, all efforts must be made to offer patients this type of treatment. Such patients include those with type 1 diabetes mellitus who already have end stage renal disease or who experience recurrent severe hypoglycemia or hyperglycemia despite optimal medical management through exogenous insulin administration [4]. Moreover, the proportion of patients with type-2 diabetes undergoing simultaneous pancreas and kidney transplant continues to increase, reaching 23% in 2020. Less often, pancreas transplants are undertaken for other forms of diabetes mellitus, including, cystic fibrosis-related diabetes mellitus and post pancreatectomy diabetes mellitus [5].

The authors acknowledge that during the past decade, the annual number of pancreas transplants performed in some European Countries as well as the United States has steadily declined [6, 7]. This trend is related to a number of factors but predominantly is due to the susceptibility of the pancreas to ischemia-reperfusion injury. As all categories of beta cell replacement are life-enhancing and life-extending procedures, an initiative is needed to “re-invigorate” the rates of pancreas donation while maintaining organ quality. The impact MP has shown promise in other areas of organ transplantation inspiring research in the field of beta-cell replacement. It is hoped further studies relating to MP will improve pancreas utilization for both whole pancreas and islet transplantation.

### Description of Target Population

Preservation of the pancreas is critical to maintain function of the organ and tissue during storage and has been the focus of research for decades [8, 9]. The gold-standard method for human beta cell preservation is hypothermic preservation by static cold storage (SCS), but may be insufficient when processing the marginal pancreases, thus opening up the possibility of improvement by new technologies [10, 11]. Recent innovations have focused on expanding the pancreas donor pool, including organs from older donors, those with higher BMI and those recovered from controlled donation after circulatory death (cDCD) [12–14]. In this sense, machine perfusion, in its’ hypothermic, normothermic or persufflation modalities, could be the key to improving the quality of the donor pancreas as well as the pool of organs available for pancreas and islet transplantation [15, 16].

In contrast to other solid organs [17], the role of *in-situ* Normothermic Regional Perfusion after cDCD is emerging whilst *ex situ* machine perfusion for pancreas and islet transplant is in its infancy. The intrinsic characteristics of the pancreas, with its low blood flow and complex vascular anatomy, makes it highly susceptible to ischemic injury during preservation, resulting in detrimental effects on the organ’s microcirculation [18]. This makes the design of the conditions, and the perfusion parameters of machines, more complex than for other solid organs and thus calls for further expert evaluation. Machine perfusion allows “real-time” investigations of the organ including perfusate analysis which opens the potential for objective assessment criteria for transplantation, a situation which has yet to be established. This would prove to be invaluable for the assessment and utilization of “marginal organs^”^ [19]. There are further gains to be translated clinically with the strategy of persufflation demonstrated to extend the duration of preservation and improve subsequent isolated viable islet yields [20].

Besides these initial encouraging data, preservation technologies still await a breakthrough. The relevant literature cites studies encompassing small numbers with varied protocols and outcome measures [21]. Because of this, it is imperative to develop optimal assessment parameters to evaluate organ quality and viability. Recent experimental animal and human models of *ex-situ* pancreas MP appear promising [22–24]. However, application of MP to the pancreas requires standardization that considers the unique characteristics of the pancreas and includes the highest quality evidence to inform MP protocols.

In the current era of MP technology, a consensus report is needed to define the role of pancreas machine perfusion. This consensus document, acknowledges the notable progress made in the research field of pancreas and islet MP and attempts to begin the bridge to clinical realization. This should, in turn, change the work dynamics for the transplant community, facilitating decision making based on objective morphological and functional criteria. MP could provide the paradigm shift providing opportunities for assessment, drug therapies, cellular therapies and facilitate further research and innovation.

### Aim of the Guideline

To address the role of pancreas machine perfusion in increasing the donor pool for beta cell replacement, the European Society for Organ Transplantation (ESOT) assembled a consensus conference within the Transplantation Learning Journey 3.0 (TLJ3.0) framework. The Working Group comprised a global panel of experts in islet and pancreas transplantation: biomedical science researchers, biologists, transplant surgeons, urologists, endocrinologists, and pathologists. Guidelines on key aspects of pancreatic MP experimental models were developed examining their potential benefits, technical aspects, and their clinical implications. In addition, a group of senior jurors from the field was present during all proceedings. Summaries of the evidence were presented to the entire group of expert panelists and jurors. The consensus findings and recommendations of the ESOT Consensus guideline on the “Role of pancreas machine perfusion to increase the donor pool for beta cell replacement,” are presented in this document for healthcare providers involved in this field. This guideline will be updated over time to reflect new evidence as it becomes available.

## Methods

The consensus development process was organized by a dedicated Guidelines Taskforce within ESOT and its sections ELITA, EKITA, EPITA, ECTTA, ETHAP, Education Committee, YPT, Transplant International editorial board members and patient representatives. The detailed description of methodology used was reported previously [25].

Briefly, key issues related to the topic, namely: “Role of pancreas machine perfusion to increase the donor pool for beta cell replacement” were identified by the working group, and specific clinical questions were formulated according to the PICO methodology (PICO = Population, Intervention, Comparator and Outcome) [25]. All PICO questions are listed in Table 1. Following the definition of the PICOs, literature searches (not preregistered) were developed by expert staff (with extensive systematic review experience) from the Centre for Evidence in Transplantation (CET) and were subsequently integrated, when needed, by the steering committee experts (Supplementary Appendices S1, S2).

**TABLE 1:** PICO question on the topic “Role of pancreas machine perfusion to increase the donor pool for beta cell replacement”.

***Ex-situ* hypothermic machine perfusion in whole pancreas transplantation**	
PICO 1	For whole pancreas transplantation, should *ex-situ* hypothermic machine perfusion be performed at a pressure less than 30 mmHg?
PICO 2	For whole pancreas transplantation, should *ex-situ* hypothermic machine perfusion be beneficial if the duration is more than 1 h and less than 6 h?
PICO 3	For whole pancreas transplantation, should *ex-situ* hypothermic machine perfusate temperature be maintained at a range between 4°C and 12°C?
PICO 4	For whole pancreas transplantation, should *ex-situ* hypothermic machine perfusion be performed with Belzer-MPS or IGL-1?
PICO 5	For whole pancreas transplantation, could *ex-situ* hypothermic machine perfusion be performed by continuous or pulsatile perfusion?
PICO 6	Should *ex-situ* hypothermic machine perfusion for whole pancreas transplantation be performed simultaneously through the superior mesenteric artery and the splenic artery?
PICO 7	For whole pancreas transplantation, should *ex-situ* hypothermic machine perfusion be performed after a completed back table preparation to reduce organ leakage?
PICO 8	Does the decrease in resistance indexes during *ex-situ* hypothermic machine perfusion correlate with better preservation of the whole pancreas?
***Ex-situ* normothermic perfusion in whole pancreas transplantation**	
PICO 1	Could *ex-situ* normothermic machine perfusion be a method for evaluating whole pancreas after cold preservation for whole pancreas transplantation?
PICO 2	For whole pancreas transplantation, should *ex-situ* normothermic machine perfusion be performed at temperatures ranging from 34°C to 37°C, with a perfusate solution containing an oxygen carrier?
PICO 3	For whole pancreas transplantation, should *ex-situ* normothermic machine perfusion be performed at a maintenance pressure range from 25 to 50 mmHg?
PICO 4	For whole pancreas transplantation, does *ex-situ* normothermic machine perfusion require a balance of pressure and flow to ensure minimal damage to the endothelium?
PICO 5	In *ex-situ* normothermic machine perfusion for whole pancreas transplantation, does the addition of an oncotic factor to the perfusate ensure there is an oncotic pressure to minimize edema formation?
PICO 6	For whole pancreas transplantation, should *ex-situ* normothermic machine perfusion be beneficial if the duration is more than 1 h and less than 6 h?
PICO 7	For whole pancreas transplantation, could *ex-situ* normothermic machine perfusion be performed by continuous or pulsatile perfusion?
PICO 8	In case of prolonged perfusion, does *ex-situ* normothermic machine perfusion require the management of exocrine secretions to potentially prevent the development of tissue injury?
PICO 9	During *ex-situ* normothermic machine perfusion for pancreas transplantation, could the endocrine function of the pancreas graft be assessed by hormone secretion tests?
PICO 10	During *ex-situ* normothermic machine perfusion for pancreas transplantation, could preservation of pancreatic exocrine function be assessed by amylase and lipase levels in the perfusate?
PICO 11	Should *ex-situ* normothermic machine perfusion for pancreas transplantation be performed simultaneously through the superior mesenteric artery and the splenic artery?
***In-situ* normothermic regional perfusion in whole pancreas transplantation**	
PICO 1	Is *in-situ* normothermic regional perfusion a reliable and reproducible method for donation after cDCD in the scenario of whole pancreas transplantation?
PICO 2	For whole pancreas transplantation, is *in-situ* normothermic regional perfusion in the setting of cDCD compatible with the procurement of liver and kidneys?
PICO 3	For whole pancreas transplantation, is *in-situ* normothermic regional perfusion in the setting of cDCD compatible with the procurement of heart and lungs?
PICO 4	Should post-mortem *in-situ* normothermic regional perfusion in the setting of cDCD be run for a duration of 1–4 h in the context of whole pancreas transplantation?
PICO 5	Should valid parameters (machine perfusion-monitoring flow and temperature, analytical/biochemical parameters, and functional warm ischemia time) be defined to assess the quality of the pancreatic graft before deciding the suitability/validity of the organ for whole pancreas transplant?
PICO 6	Could *in-situ* normothermic regional perfusion in donation in the setting of cDCD improve graft and patient outcomes compared with *in-situ* cooling and rapid procurement in whole pancreas transplantation?
PICO 7	Does *in-situ* normothermic regional perfusion in the setting of cDCD have the potential to expand the donor pool for whole pancreas transplantation?
***Ex-situ* hypothermic machine perfusion in islets transplantation**	
PICO 1	Should *ex-situ* hypothermic perfusion of the pancreas for islet isolation be performed in the same manner as for vascularized pancreas transplantation with regards to: temperature, pressure, perfusate composition, oxygenation, duration, and timing?
PICO 2	In islet transplantation, could *ex-situ* hypothermic perfusion be used to increase cellular energy reserves, especially in controlled donation after circulatory death procedures?
PICO 3	Could *ex-situ* hypothermic perfusion be used to avoid night-time islet isolations?
***Ex-situ* normothermic perfusion in islets transplantation**	
PICO 1	Could *ex-situ* normothermic machine perfusion be a reliable method for evaluating whole pancreases after cold preservation in islet transplantation?
PICO 2	In islet transplantation, should *ex-situ* machine perfusion be performed at physiologic temperature, with perfusate solution containing an oxygen carrier to sustain metabolic activities of the cells?
PICO 3	In islet transplantation, should *ex-situ* normothermic machine perfusion be performed at a maintenance pressure range from 25 to 50 mmHg?
PICO 4	In islet transplantation, does *ex-situ* normothermic machine perfusion require a balance of pressure and flow to ensure minimal damage to the endothelium?
PICO 5	In *ex situ* normothermic machine perfusion for islet transplantation, does the addition of an oncotic factor to the perfusate ensure there is an oncotic pressure to minimize edema formation?
PICO 6	In islet transplantation, should *ex-situ* normothermic machine perfusion be beneficial if the duration is more than 1 h and less than 6 h?
PICO 7	In islet transplantation, could *ex-situ* normothermic machine perfusion be performed continuous or pulsatile perfusion?
PICO 8	In the case of prolonged perfusion, does *ex-situ* normothermic machine perfusion require the management of exocrine secretions to prevent the development of tissue injury?
PICO 9	During *ex-situ* normothermic machine perfusion for islet transplantation, could the endocrine function of the pancreas graft be assessed by hormone secretion tests?
PICO 10	During *ex-situ* normothermic machine perfusion for islet transplantation, could preservation of pancreatic exocrine function be assessed by amylase and lipase levels in the perfusate?
PICO 11	Should *ex-situ* normothermic machine perfusion for islet transplantation be performed simultaneously through the superior mesenteric artery and the splenic artery?
***In-situ* normothermic regional perfusion in islets transplantation**	
PICO 1	Is *in-situ* normothermic regional perfusion in the setting of cDCD a reliable and reproducible method for donation after controlled circulatory death in the scenario of islet transplantation?
PICO 2	For islet transplantation, is *in-situ* normothermic regional perfusion in the setting of cDCD compatible with the procurement of other abdominal organs (kidneys, liver)?
PICO 3	For islet transplantation, is *in-situ* normothermic regional perfusion in the setting of cDCD compatible with the procurement of thoracic organs (heart, lungs)?
PICO 4	Should post-mortem *in-situ* normothermic regional perfusion in the setting of cDCD be run for a duration 1–4 h in the context of islet transplantation?
PICO 5	Should valid parameters (machine perfusion-monitoring flow and temperature, analytical/biochemical parameters, and functional warm ischemia time) be defined to assess the quality of the pancreatic graft before deciding the suitability/validity of the organ for islet transplant?
PICO 6	Could *in-situ* normothermic regional perfusion in donation after controlled circulatory death improve isolation outcomes (yield, function, and viability) and post transplantation outcomes compared to *in-situ* cooling and rapid procurement in islet transplantation?
PICO 7	Does the *in-situ* normothermic regional perfusion in the setting of cDCD have the potential to expand the donor pool for islet transplantation?
**Persufflation in islets transplantation**	
PICO 1	In islet transplantation, should persufflation be performed using a humidified gaseous flow of 40% oxygen and 60% nitrogen?
PICO 2	Should persufflation be performed at a temperature of 4°C–8°C in an organ preservation solution?
PICO 3	Should persufflation be performed using a gaseous flow rate of 20–25 mL/hr?
PICO 4	Should persufflation be performed by canulation of the superior mesenteric artery and the splenic artery and optionally the pancreaticoduodenal artery?
PICO 5	Should arterial leakages be closed until the gaseous outflow is mainly venous when starting persufflation?
PICO 6	Can persufflation be used to prevent further cold ischemic damage for up to 24 h?
PICO 7	Can persufflation be performed during organ transport or as an end-ischemic strategy?
PICO 8	Can persufflation attenuate pro-inflammatory signaling in isolated islets?

cDCD, controlled Donation after Circulatory Death.

The Transplant Library was searched on 30 October 2022. The Transplant Library includes all randomized controlled trials and systematic reviews in the field of solid organ transplantation published as full text or in abstract form, sourced mainly from MEDLINE/PubMed and hand-searches of congress proceedings. The search strategy used is as follows: (Pancreas transplantation or pancreas or islets of Langerhans transplantation or islet) and (perfusion or organ preservation or persufflation or perfusion or preservation or two layer method or two-layer method or TLM). Searches were expanded to include non-randomized studies. MEDLINE and EMBASE were searched on 30 October 2022 using the search strategy below: (Pancreas transplantation or pancreas transplant or islets of Langerhans transplantation or islet or organ transplant or simultaneous pancreas kidney or simultaneous pancreas-kidney or SPK) and (persufflation or two layer method or two-layer method or TLM or cardiopulmonary bypass or heart-lung bypass or extracorporeal circulation or extracorporeal membrane oxygenation or ECMO or regional perfusion or machine perfusion or perfusion or *ex-situ* perfusion or oxygenation or hypothermic perfusion or normothermic perfusion). Citations in articles were then reviewed and analyzed to extract unidentified articles.

**FIGURE 1:**
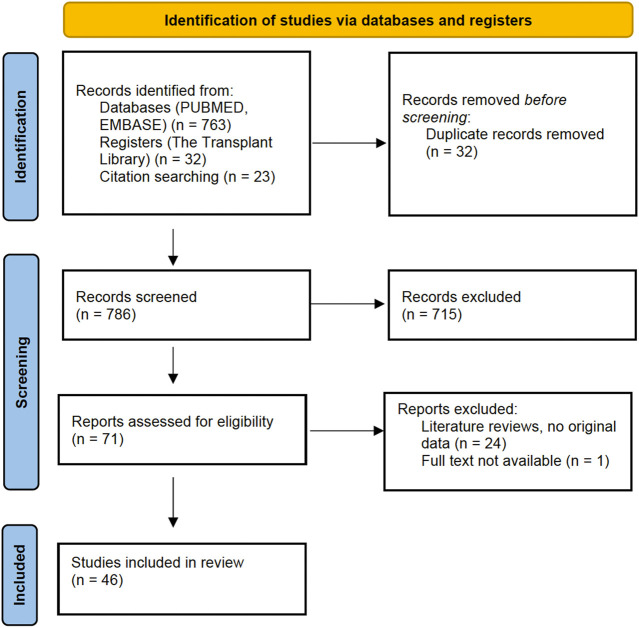
PRISMA Flowchart: Literature search.

A PRISMA flowchart describing the number of studies identified by the literature search and number of studies selected for inclusion (Supplementary Appendix S3) in the consensus statement appears in Figure 1.

A summary of the evidence addressing each key question by the included studies was prepared in an evidence table and sent to all members of the workgroup and the jury (Supplementary Appendix S4). The workgroup proposed a recommendation for each key question, based on the quality of evidence rated using the GRADE approach, with high quality rated as A, medium quality as B, and low quality as C. Very low quality of evidence was not considered. For evaluation of the quality of evidence according to GRADE [26] the following features were considered: study design, risk of bias, inconsistency, indirectness, imprecision, number of patients, effect, importance, and publication bias. Strength of recommendation was rated as 1 (strong) or 2 (weak). The Delphi method was applied with a view to reaching a group opinion/consensus during the conference. For each PICO question, recommendation, quality of the evidence and strength of the recommendation were voted on by an independent jury (4 members). Each recommendation was retained if more than 3 jury members agreed with it.

Complete information including: the list of consensus conference workgroup domains (and topics noted below); the process regarding consensus conference participant selection; development and refinement of consensus statements, and modified Delphi methodology including consensus polling, were previously reported in the in-person conference held in Prague, Czech Republic, 13–15 November 2022 [25].

## Results

### *Ex-Situ* Hypothermic Machine Perfusion in Whole Pancreas Transplantation

No clinical studies are presently reported regarding the implementation of *ex-situ* hypothermic machine perfusion in whole pancreas transplantation. All studies are pre-clinical studies in animal or human models. No human whole pancreas transplants have been performed after preservation with hypothermic machine perfusion. Therefore, the quality of evidence was Grade C for all recommendations. The strength of recommendation was 1 for 6 recommendations and 2 for 2 recommendations.

Recommendation 1.1: For whole pancreas transplantation, *ex-situ* hypothermic machine perfusion should be performed up to a pressure of 30 mmHg.

Quality of Evidence: C.

Strength of Recommendation: 1.

Recommendation 1.2: For whole pancreas transplantation, *ex-situ* hypothermic machine perfusion should be performed for a duration greater than 1 h but less than 6 h.

Quality of Evidence: C.

Strength of Recommendation: 2.

Recommendation 1.3: For whole pancreas transplantation, non-oxygenated hypothermic perfusate temperature should be maintained at a temperature range between 4°C and 12°C.

Quality of Evidence: C.

Strength of Recommendation: 1.

Recommendation 1.4: *Ex-situ* hypothermic machine perfusion should be performed with a colloid based solution, clinically licensed for machine use (for abdominal organs).

Quality of Evidence: C.

Strength of Recommendation: 1.

Recommendation 1.5: For whole pancreas transplantation, *ex-situ* hypothermic machine perfusion can be performed by either continuous or pulsatile perfusion.

Quality of Evidence: C.

Strength of Recommendation: 2.

Recommendation 1.6: *Ex-situ* hypothermic machine perfusion for whole pancreas transplantation must be performed simultaneously through the superior mesenteric artery and the splenic artery.

Quality of Evidence: C.

Strength of Recommendation: 1.

Recommendation 1.7: For whole pancreas transplantation a complete back table preparation must be performed prior to *ex-situ* hypothermic machine perfusion to reduce leakage of the perfusate.

Quality of Evidence: C.

Strength of Recommendation: 1.

Recommendation 1.8: During *ex-situ* hypothermic machine perfusion, a decrease in resistance index may be correlated with better preservation of the whole pancreas.

Quality of Evidence: C.

Strength of Recommendation: 1.

### *Ex-Situ* Normothermic Perfusion in Whole Pancreas Transplantation

No clinical studies are available regarding the implementation of *ex-situ* normothermic perfusion in whole pancreas transplantation. All studies are pre-clinical studies in either animal or human models. No human whole pancreas transplants have been performed after preservation with *ex-situ* normothermic perfusion. Therefore, the quality of evidence was Grade C for all recommendations. The strength of recommendation was 1 for 9 recommendations and 2 for 2 recommendations.

Recommendation 2.1: *Ex-situ* normothermic machine perfusion can be a method for evaluating the whole pancreas after cold preservation.

Quality of Evidence: C.

Strength of Recommendation: 1.

Recommendation 2.2: For whole pancreas transplantation, *ex-situ* normothermic machine perfusion with a perfusate solution containing an oxygen carrier should be performed within a temperature range of 34°C–37°C.

Quality of Evidence: C.

Strength of Recommendation: 1.

Recommendation 2.3: For whole pancreas transplantation, *ex-situ* normothermic machine perfusion should be performed at a maintenance pressure range from 25 to 50 mmHg.

Quality of Evidence: C.

Strength of Recommendation: 1.

Recommendation 2.4: For whole pancreas transplantation, *ex-situ* normothermic machine perfusion requires a balance of pressure and flow to preserve the endothelium.

Quality of Evidence: C.

Strength of Recommendation: 1.

Recommendation 2.5: In *ex-situ* normothermic machine perfusion for whole pancreas transplantation, addition of oncotic agents to the perfusate could help to minimize graft edema.

Quality of Evidence: C.

Strength of Recommendation: 1.

Recommendation 2.6: For whole pancreas transplantation, *ex-situ* normothermic machine perfusion should be performed for a duration longer than 1 h.

Quality of Evidence: C.

Strength of Recommendation: 1.

Recommendation 2.7: For whole pancreas transplantation, *ex-situ* normothermic machine perfusion can be performed by either continuous or pulsatile perfusion.

Quality of Evidence: C.

Strength of Recommendation: 2.

Recommendation 2.8: *Ex-situ* normothermic machine perfusion for whole pancreas transplantation requires diversion of exocrine secretions to prevent tissue injury.

Quality of Evidence: C.

Strength of Recommendation: 2.

Recommendation 2.9: During *ex-situ* normothermic machine perfusion for whole pancreas transplantation, the endocrine function of the pancreas graft can be assessed by hormone secretion tests.

Quality of Evidence: C.

Strength of Recommendation: 1.

Recommendation 2.10: During *ex-situ* normothermic machine perfusion for whole pancreas transplantation, amylase, and lipase perfusate levels are not reliable exocrine markers for tissue viability or injury.

Quality of Evidence: C.

Strength of Recommendation: 1.

Recommendation 2.11: *Ex-situ* normothermic machine perfusion for whole pancreas transplantation must be performed simultaneously through the superior mesenteric artery and the splenic artery.

Quality of Evidence: C.

Strength of Recommendation: 1.

### *In-Situ* Normothermic Regional Perfusion in Whole Pancreas Transplantation

Regarding the implementation of *in-situ* normothermic regional perfusion in whole pancreas transplantation, a total of nine studies reported outcomes after cDCD pancreas transplantation have been published so far. These are cohort (*n* = 2) or case studies (*n* = 7). A total of 59 human whole pancreas transplants have been reported in the literature. The quality of evidence was Grade A for one recommendation and C for 7 recommendations. The strength of recommendation was 1 for 5 recommendations and 2 for 2 recommendations.

Recommendation 3.1: *In-situ* normothermic regional perfusion is a reliable and reproducible method for donation after controlled circulatory death in the scenario of whole pancreas transplantation.

Quality of Evidence: C.

Strength of Recommendation: 1.

Recommendation 3.2: For whole pancreas transplantation, *in-situ* normothermic regional perfusion in the setting of cDCD is compatible with the procurement of liver and kidneys.

Quality of Evidence: A.

Strength of Recommendation: 1.

Recommendation 3.3: For whole pancreas transplantation, *in-situ* normothermic regional perfusion in the setting of cDCD is compatible with the procurement of heart and lungs.

Quality of Evidence: C.

Strength of Recommendation: 1.

Recommendation 3.4: In the context of whole pancreas transplantation, *in-situ* normothermic regional perfusion in the setting of cDCD should be maintained between 1 and 4 h.

Quality of Evidence: C.

Strength of Recommendation: 1.

Recommendation 3.5: In the context of whole pancreas transplantation after *in-situ* normothermic regional perfusion of cDCD, valid assessment parameters of graft quality still need to be defined.

Quality of Evidence: C.

Strength of Recommendation: 1.

Recommendation 3.6: For whole pancreas transplantation, *in-situ* normothermic regional perfusion in the setting of cDCD might improve graft and patient outcomes when compared to *in-situ* cooling and rapid procurement.

Quality of Evidence: C.

Strength of Recommendation: 2.

Recommendation 3.7: *In-situ* normothermic regional perfusion in the setting of cDCD has the potential to expand the donor pool for whole pancreas transplantation.

Quality of Evidence: C.

Strength of Recommendation: 2.

### *Ex-Situ* Hypothermic Machine Perfusion in Islets Transplantation

Regarding the implementation of *ex-situ* hypothermic machine perfusion in islet transplantation, no clinical studies are available. All studies are pre-clinical in either animal or human models. No human islet transplants have been performed after preservation with hypothermic machine perfusion. Therefore, the quality of evidence was Grade C for all recommendations. The strength of recommendation was 1 for 3 recommendations.

Recommendation 4.1: *Ex-situ* hypothermic perfusion of the pancreas for islet transplantation should be performed in the same manner as for whole pancreas transplantation with the addition of oxygenation.

Quality of Evidence: C.

Strength of Recommendation: 1.

Recommendation 4.2: In the context of pancreas for islet transplantation, oxygenated *ex-situ* hypothermic perfusion could be used to increase cellular ATP levels, especially during recovery from cDCD.

Quality of Evidence: C.

Strength of Recommendation: 1.

Recommendation 4.3: In the context of pancreas for islet transplantation, oxygenated *ex-situ* hypothermic machine perfusion has the potential to prolong cold preservation times, which may be helpful for logistical considerations in islet isolation and transplantation.

Quality of Evidence: C.

Strength of Recommendation: 1.

### *Ex-Situ* Normothermic Perfusion in Islets Transplantation

Regarding the implementation of *ex-situ* normothermic perfusion in islet transplantation, given the absence of references describing the use of *ex-situ* normothermic perfusion in islet transplantation, the same PICO questions have been raised as described for whole pancreas transplantation. In this sense, we consider that recommendations can be extrapolated but with a low level of evidence. Therefore, the quality of evidence was Grade C for all recommendations. The strength of recommendation was 1 for 9 recommendations and 2 for 2 recommendations.

Recommendation 5.1: *Ex-situ* normothermic machine perfusion has the potential for evaluating the donor pancreas after cold preservation for islet transplantation.

Quality of Evidence: C.

Strength of Recommendation: 2.

Recommendation 5.2: For islet transplantation, *ex situ* normothermic machine perfusion with a perfusate solution containing an oxygen carrier should be performed within a temperature range of 34°C–37°C.

Quality of Evidence: C.

Strength of Recommendation: 1.

Recommendation 5.3: If *ex situ* normothermic machine perfusion of the whole pancreas is to be performed prior to islet transplantation, it should be carried out at a maintenance pressure ranging between 25–50 mmHg.

Quality of Evidence: C.

Strength of Recommendation: 2.

Recommendation 5.4: During *ex-situ* normothermic machine perfusion of the pancreas prior to islet transplantation, consideration for pressure and flow is necessary to minimize injury to the endothelium.

Quality of Evidence: C.

Strength of Recommendation: 1.

Recommendation 5.5: During *ex-situ* normothermic machine perfusion of the pancreas for islet transplantation, the addition of oncotic agents to the perfusate could help to minimize graft edema.

Quality of Evidence: C.

Strength of Recommendation: 1.

Recommendation 5.6: *Ex-situ* normothermic machine perfusion of the pancreas for islet transplantation should be performed for a duration longer than 1 h.

Quality of Evidence: C.

Strength of Recommendation: 1.

Recommendation 5.7: *Ex-situ* normothermic machine perfusion of the pancreas prior to islet transplantation can be performed by either continuous or pulsatile perfusion.

Quality of Evidence: C.

Strength of Recommendation: 1.

Recommendation 5.8: *Ex-situ* normothermic machine perfusion of the pancreas prior to islet transplantation requires diversion of exocrine secretions to prevent tissue injury.

Quality of Evidence: C.

Strength of Recommendation: 1.

Recommendation 5.9: During *ex-situ* normothermic machine perfusion of the pancreas for islet transplantation, the endocrine function of the pancreas can be assessed by hormone secretion tests.

Quality of Evidence: C.

Strength of Recommendation: 1.

Recommendation 5.10: During *ex-situ* normothermic machine perfusion of the pancreas for islet transplantation, amylase and lipase perfusate levels are not reliable exocrine markers for tissue viability or injury.

Quality of Evidence: C.

Strength of Recommendation: 1.

Recommendation 5.11: *Ex-situ* normothermic machine perfusion of the pancreas for islet transplantation must be performed simultaneously through the superior mesenteric artery and the splenic artery.

Quality of Evidence: C.

Strength of Recommendation: 1.

### *In-Situ* Normothermic Regional Perfusion in Islets Transplantation

Regarding the implementation of *in-situ* normothermic regional perfusion in islet transplantation, a total of 2 studies reporting outcome after cDCD pancreas transplants have been published so far. A total of 5 clinical islet transplants have also been reported in the literature. The quality of evidence was Grade C for all recommendations. The strength of recommendation was 1 for 7 recommendations.

Recommendation 6.1: *In-situ* normothermic regional perfusion is a reliable and reproducible method for recovery of the pancreas in a cDCD when utilized in the scenario of islet transplantation.

Quality of Evidence: C.

Strength of Recommendation: 1.

Recommendation 6.2: In the context of pancreas for islet transplantation, *in-situ* normothermic regional perfusion in the setting of cDCD is compatible with the procurement of liver and kidneys.

Quality of Evidence: C.

Strength of Recommendation: 1.

Recommendation 6.3: In the context of pancreas for islet transplantation, *in-situ* normothermic regional perfusion in the setting of cDCD is compatible with the procurement of heart and lungs.

Quality of Evidence: C.

Strength of Recommendation: 1.

Recommendation 6.4: In the context of pancreas for islet transplantation, *in-situ* normothermic regional perfusion in the setting of cDCD should be maintained between 1 and 4 h.

Quality of Evidence: C.

Strength of Recommendation: 1.

Recommendation 6.5: Valid assessment parameters of pancreas graft quality still need to be defined in the context of pancreas for islet transplantation after *in-situ* normothermic regional perfusion for cDCD.

Quality of Evidence: C.

Strength of Recommendation: 1.

Recommendation 6.6: *In-situ* normothermic regional perfusion in donation after cDCD may improve islet isolation and transplant outcomes compared to *in-situ* cooling and rapid procurement.

Quality of Evidence: C.

Strength of Recommendation: 1.

Recommendation 6.7: *In-situ* normothermic regional perfusion in the setting of cDCD has the potential to expand the donor pool for islet transplantation.

Quality of Evidence: C.

Strength of Recommendation: 1.

### Persufflation in Islet Transplantation

No clinical studies are available regarding the implementation of persufflation in islet transplantation. All studies are pre-clinical in either animal or human models. No human islet transplants have been reported after preservation with persufflation. Therefore, the quality of evidence was Grade C for all recommendations. The strength of recommendation was 1 for 6 recommendations and 2 for 1 recommendation. The jury could not deliberate on one query (PICO 8) due to lack of evidence.

Recommendation 7.1: In the context of pancreas for islet transplantation, persufflation should be performed using a humidified gaseous flow of 40% oxygen.

Quality of Evidence: C.

Strength of Recommendation: 1.

Recommendation 7.2: In the context of pancreas for islet transplantation, persufflation should be performed at a temperature of 4°C–8°C in an organ preservation solution.

Quality of Evidence: C.

Strength of Recommendation: 1.

Recommendation 7.3: In the context of pancreas for islet transplantation, persufflation should be performed using a gaseous flow rate of 20–25 mL/hr.

Quality of Evidence: C.

Strength of Recommendation: 1.

Recommendation 7.4: In the context of pancreas for islet transplantation, persufflation can be performed by cannulation of both the superior mesenteric artery and the splenic artery and optionally, the gastroduodenal artery.

Quality of Evidence: C.

Strength of Recommendation: 1.

Recommendation 7.5: In the context of pancreas for islet transplantation, a back table preparation must be performed prior to persufflation to stop arterial gaseous leaks.

Quality of Evidence: C.

Strength of Recommendation: 1.

Recommendation 7.6: In the context of pancreas for islet transplantation, persufflation has the potential to prolong cold preservation up to 24 h.

Quality of Evidence: C.

Strength of Recommendation: 1.

Recommendation 7.7: In the context of pancreas for islet transplantation, persufflation can be performed during organ transport or as an end-ischemic strategy.

Quality of Evidence: C.

Strength of Recommendation: 2.

## Summary and Next Steps

Improved preservation of the pancreas with a view to undertaking either whole pancreas or islet transplantation is a particularly interesting and developing field. In recent years there has been a significant increase in the number of pre-clinical and clinical studies reporting the use of hypothermic and normothermic machine perfusion as well as normothermic regional perfusion. However, there are very few studies relating to pancreas and islet transplantation. With the likely development of this field, it is important to have consensus to allow systematic reporting and comparison.

The clinical implementation of normothermic regional perfusion is the only modality that has been successfully reported for both whole pancreas and islet transplantation. None of the other preservation modalities have been implemented in pancreas and islet transplantation and can therefore not be considered at present as a preservation modality that can be used in humans. Normothermic regional perfusion is already considered the standard of care in a minority of European countries but it is hoped that further funding in other countries will support its wider application. These data highlight the major difficulty in obtaining high quality data on organ preservation for either whole pancreas transplantation or islet transplantation, when compared to other organ transplants (kidney-liver) where it has been more widely reported. These ESOT TLJ3.0 guidelines are the first to specifically address perfusion preservation modalities in both pancreas and islet transplantation. Despite the lack of high-quality data, these guidelines define the current technical modalities of perfusion in both hypothermic and normothermic conditions. They also aim to define the main outcomes expected in various perfusion modalities. The development of a consensus on perfusion modalities through preclinical studies allows us to consider high quality clinical studies to accurately assess the role of perfusion in whole pancreas and islet transplantation. In the authors’ opinion, this consensus provides the technical basis to guide future clinical studies with a view to conducting the first human clinical feasibility trials.

## Data Availability

The datasets presented in this study can be found in online repositories. The names of the repository/repositories and accession number(s) can be found in the article/Supplementary Material.

## Supplementary Material

SUPPLEMENTARY APPENDIX S1 Evidence report - Pancreas Machine Perfusion.

SUPPLEMENTARY APPENDIX S2 Congress abstracts Pancreas Machine Perfusion.

SUPPLEMENTARY APPENDIX S3 Studies included in review.

SUPPLEMENTARY APPENDIX S4 Preliminary Statements Draft - Role of Pancreas Machine Perfusion to Increase the Donor Pool for Beta Cell Replacement.

## Abbreviations

cDCD, Controlled Donation after Circulatory Death; CET, Centre for Evidence in Transplantation; ECTTA, European Cardio Thoracic Transplant Association; EKITA, European Kidney Transplant Association; ELITA, European Liver and Intestine Transplant Association; EPITA, European Pancreas and Islet Transplant Association; ESOT, European Society for Organ Transplantation; ETAHP, European Transplant Allied Healthcare Professionals; GRADE, Grading of Recommendations, Assessment, Development and Evaluation; MP, Machine Perfusion; PICO, Population, Intervention, Comparator and Outcome; PRISMA, Preferred Reporting Items for Systematic Reviews and Meta-Analyses; SCS, Static cold storage; TJL 3.0, Transplantation Learning Journey 3.0; YPT, Young Professionals in Transplantation.
