# Supplementary material for: European Society for Organ Transplantation (ESOT) Consensus Statement on the Role of Pancreas Machine Perfusion to Increase the Donor Pool for Beta Cell Replacement Therapy
Source: Transpl Int. 2023 Jul 19;36:11374. doi: 10.3389/ti.2023.11374 (PMC10402633; doi:10.3389/ti.2023.11374)
Supplement: Supplementary file 1 [file DataSheet2.pdf]

Bellini, M. I., et al. (2019). "Towards the temperature paradigm in machine perfusion preservation: A systematic review of animal models." Transplant International **32(Supplement 2)**: 127-128.

Background: There is limited knowledge in the effects of perfusion temperature on intrinsic cell metabolism, which in turns governs the extent of injury and function of grafts in recipients. Molecular parameters of ischaemiareperfusion injury include mRNA expression of pro-inflammatory cytokines, ATP and cellular injury. Method(s): A systematic search in databases (Embase, Medline, Cochrane Library, Transplant Library) generated 10,585 studies, with 134 studies included. Result(s): A novel study demonstrated that a combined liver-kidney normothermic machine perfusion (NMP) is associated with an increase in graft tissue ATP and a decrease in liver injury enzyme levels, AST/ALT. Other NMP studies showed that a higher temperature could also be associated with increase in graft function. The impact of perfusion temperature on the increase in tissue graft ATP compared to conventional static cold storage (SCS) was analysed as a measure of metabolic recovery, cellular oxygen consumption and function. This association was demonstrated between hypothermic machine perfusion (HMP) versus SCS in 4 liver studies, with limited data in NMP. An analysis of 3 studies on the preservation temperature on pancreas graft function revealed a lack of consensus on the optimal temperature associated with lower rate of DGF and IRI. Two studies suggested that a range of (7-10degreeC) was superior to a hypothermic (0-4degreeC) one. Qualitative representation of novel findings was common in different studies, therefore, more quantitative studies are needed for further investigation of underlying physiological mechanisms behind differences in outcomes from SCS and MP. Conclusion(s): There are emerging animal studies suggesting that different machine perfusion temperatures could influence intrinsic cellular and tissue metabolism, reflected by a reduction in delayed graft function. More studies are needed for further investigation of underlying physiological mechanisms behind differences in outcomes from SCS and MP. (Table Presented).

19th Congress of the European Society for Organ Transplantation. Copenhagen Denmark

Branchereau, J., et al. (2017). "Hypothermic pulsatile machine perfusion for human pancreas preservation." American Journal of Transplantation **17(Supplement 3)**: 683.

Pancreas transplantation is one of the best treatment for selected insulin-dependent diabetic patients. The pancreas is high vulnerable to ischemic damage and reperfusion injury which could end into graft pancreatitis and thrombosis. Static cold storage is the only preservation method for pancreas preservation. Hypothermic machine perfusion (HMP) has proved its value in preserving kidney transplants. Whether HMP is a safe procedure for human pancreas preservation is unknown. The first pancreas pulsatile perfusion was done in canine pancreas autografts in the eighties; this was a high pressure perfusion and induced severe edema. Today, there is no clinical study designed to investigate the impact of HMP on human pancreas allografts. In our pre-clinical study, 9 human pancreas from deceased heart beating donors excluded for clinical transplantation were used and perfused with the Wave (Waters Medical Systems) perfusion machine. Pancreas were prepared identically as for human vascularized organ transplantation. The splenic artery and superior mesenteric artery were anastomosed to an iliac artery division. All pancreas were perfused with a pressure of 25 mmHg (Figur presented). Tissue biopsies were collected at baseline and after 6, 12 and 24 hours of HMP. Histology did not demonstrate edema after 6, 12 hours; some edema was observed at 24 hours. Duodenum villi were unaltered up to 24 hours of perfusion. Insulin, glucagon and somatostatin staining was invariable throughout follow-up. Marginal human pancreases preserved under hypothermic pulsatile conditions did not experience edema after 12 hours or perfusion. This procedure was feasible and safe in all cases. Our next challenge will be to transplant them.

18th Congress of the European Society for Organ Transplantation. Barcelona Spain

Branchereau, J., et al. (2017). "Pancreas perfusion with hypothermic machine: Pancreas and duodenal histology up to 24 hours." Transplant International **30(Supplement 2)**: 516.

Background: Pancreas transplantation is one of the best treatments options for insulin-dependent diabetes. Pancreas is high vulnerable to ischemic damage and ischemic-reperfusion injuries. Cold storage (CS) is the mainstay preservation method. Hypothermic machine perfusion (HMP) has proved its value on the preservation of kidney transplants. Does HMP could preventing ischemic reperfusion damages in pancreas transplants ? The first pancreas pulsatile perfusion were done on canine pancreas autograft in the eighties, but this perfusions was high pressure perfusion and induced edema. No clinical studies have investigated this impact of this HMP on human pancreas grafts. Methods/Materials: In this pre clinical study, 7 human

pancreas from dead beating donors were perfused with a Wave HPM (Waters Medical Systems) and 2 pancreas were not perfused (control group) and were preserved in a cold storage solution (control group). Pancreas were prepared as transplant for a transplantation of vascularized pancreas. The splenic artery and superior mesenteric artery were anastomosed to an iliac artery division. We perfused the pancreas with a pressure of 25 mmHg. Tissue biopsies were collected at baseline and after 6 / 12 / and 24 h of HMP. Result(s): The macroscopic aspect of the pancreas was unchanged after 24 h of perfusion (image 1). The index of resistance decreased during the first hours of perfusion (image 2). Histology did demonstrate no edema after 6 / 12 / and 24 h of HMP. Immuno-histology did demonstrate that endocrine function was still maintained after perfusion for insulin, glucagon and somatostatin. Conclusion(s): We conclude that pancreas preservation by pulsatile machine perfusion is feasible and does not induced edema after up to 24 h of HMP with a low pressure. (Figure Presented).

17th American Transplant Congress, ATC 2017. Chicago, IL United States

Brandhorst, D., et al. (2005). "Adaption of neutral protease activity for islet isolation from the long-term two-layer method-stored pig pancreas." Transplantation Proceedings **37(1)**: 458-459.

Background. Islet release from the pancreas is mediated by both collagenase and neutral protease (NP), a critical effector of islet integrity. To prove the hypothesis that adjustment of NP reduces islet damage after prolonged ischemia, adult pig pancreata were digested after 7-hour preservation by the two-layer method (TLM) using a 2-component enzyme blend consisting of collagenase NB-8 and NP. Methods. After intraductal University of Wisconsin (UW) flush resected pancreata were distended with 4.4 PZ-U/g of UW-dissolved Serva collagenase either before (TLM-preloaded, n = 7) or after (TLM-postloaded, n = 10) cold storage, or for immediate processing (n = 6). NP was adjusted after preliminary experiments to respectively 1.1, 0.2, or 0.8 DMC-U/g for unstored, TLM-preloaded, or postloaded organs. Results. Purified islet yield decreased from 3670 +/- 730 islet equivalents (IEQ)/g in unstored pancreata to 1800 +/- 180 and 2080 +/- 290 IEQ/g in TLM-preloaded or postloaded organs, respectively (P < .05). Although purity was always >90%, IEQ recovery was significantly decreased in TLM-preloaded pancreata. Quality control revealed consistently high viability as determined using trypan-blue exclusion (>95%) or formazan production. Compared with unstored organs (2.47 +/- 0.36; P < .05), glucose stimulation index was reduced in TLM-preloaded (1.48 +/- 0.15) and TLM-postloaded pancreata (1.81 +/- 0.20). Normoglycemia in diabetic nude mice transplanted with islets from TLM-preloaded pancreata was transient in contrast to sustained function in the other groups. Conclusions. Significant amounts of viable pig islets can be isolated after prolonged TLM preservation by reducing NP activity. Nevertheless, early enzyme administration prior to long-term storage deteriorates islet graft function. © 2005 by Elsevier Inc. All rights reserved.

http://dx.doi.org/10.1016/j.transproceed.2004.12.035

Cantarovich, D., et al. (2016). "Preservation of human pancreas with hypothermic machine perfusion." Transplantation **100(7 Supplement 1)**: S328.

Pancreas transplantation is one of the best treatment options for insulindependent diabetes. The pancreas is high vulnerable to ischemic damage and ischemic-reperfusion injury. Graft thrombosis is the consequence of this process and accounts for the majority of graft losses. Cold storage is the mainstay preservation method applied for pancreas preservation. Hypothermic machine perfusion (HMP) has proved its value in preserving kidney transplants. Whether HMP could prevent ischemic reperfusion damage in human pancreas transplants and therefore decrese graft failure is not known. The first pancreas pulsatile perfusion was done in canine pancreas autografts in the eighties; this was a high pressure perfusion and induced severe edema. Today, there is no clinical study designed to investigate the impact of HMP on human pancreas allografts. In our pre-clinical study, 7 human pancreas from deceased heart beating donors rejected for clinical transplantation because of advanced age and/or other comorbidities, were used and perfused with the Wave (Waters Medical Systems) perfusion machine. Pancreas were prepared identically as for human transplantation of vascularized organs. The splenic artery and superior mesenteric artery were anastomosed to an iliac artery division. We therefore perfused all pancreas with a pressure of 25 mmHg. Tissue biopsies were collected at baseline and after 6, 12 and 24 hours of HMP. Histology did not demonstrate edema after 6, 12 and 24 hours of HMP. We conclude that human pancreas preservation with a pulsatile machine perfusion is feasible and does not induce edema after 24 hours or perfusion under low pressure.

http://dx.doi.org/10.1097/01.tp.0000490147.72544.1a

26th International Congress of the Transplantation Society, TTS 2016. Hongkong Hong Kong

Codas, R., et al. (2012). "Kidney graft quality after donation from uncontrolled deceased donors after cardiac arrest." Transplant International **1)**: 20.

Introduction: Kidney grafts from uncontrolled deceased donors after cardiac arrest (uDDCA) have recently been used in France to counteract organ shortage. The quality of these kidneys remains debatable. The aim of our study was to compare the outcomes and the quality of uDDCA kidneys with that of kidneys from optimal donors such as simultaneous kidney and pancreas (SPK) donors and extended-criteria donors (ECD). Method(s): 27 kidney grafts from uDDCA (mean donor age, 41) were compared with 24 kidney grafts from SPK donors (mean donor age, 26), and 30 kidney grafts from ECD (mean donor age, 66). After kidney retrieval, preservation protocol consisted in hypothermic pulsatile perfusion (RM3, Water's medical) and the organ preservation solution was UW solution. Kidneys with intrarenal vascular resistance above 0.35 mmHg/ml/mn after 6 h of perfusion were discarded. All three patient groups were non-sensitized and received the same induction and maintenance immunosuppressive therapy. The quality of the grafts was assessed by renal function and histology. GFR was estimated by MDRD formula (eGFR) at M1 (n = 80), M3 (n = 80), M6 (n = 79), M12 (n = 74), M24 (n = 70) and M36 (n = 51) and measured by inuline clearance (mGFR) at M12 (n = 66) and M36 (n = 46). Vascular lesions were analyzed in systematic kidney biopsies at M3 (n = 54) and M12 (n = 50) with the Banff 2007 classification. Interstitial fibrosis (IF) was quantitatively measured by colour image analysis. Result(s): Mean renal blood flow increased from 42 to 83 mL/min/100 g. Mean intra-renal vascular resistance decreased from 0.85 to 0.3 mmHg/ml/min/ 100 g. The warm ischemic time in the uDDCA group was higher than the two others groups (106 min, P < 0.001). In the short term, no PNF was observed and the DGF in the uDDCA group was significantly higher than in the ECD group (81 vs. 28%, P < 0.001). In the uDDCA group renal function was initially poorer but improved during the first year. However on the long term, renal function (mGFR at M36, 41.2 vs. 33.7 ml/min/1.73 m2, P = 0.09) and interstitial fibrosis score was not different in uDDCA vs ECD group (IF score at M12, 36 vs. 34% P = 0.47). Conclusion(s): Our study suggests that the quality of kidneys from uDDCA donors is similar to that of ECD and that these kidneys should be attributed to the same recipient population.

1st International Meeting on Ischemia Reperfusion Injuries in Transplantation, IMIRT 2012. Poitiers France.

Engelse, M., et al. (2013). "Hypothermal machine perfusion of human donor pancreata prior to islet isolation: A pilot study." Transplantation **6S)**: S39.

Introduction: Because of the shortage of donor pancreata for islet transplantation using pancreas from extended criteria donors (ECD) is an option to increase the donor pool. Of the ECD donors, defined as DBD donors with age>45 yrs and/or DCD donors, especially the DCD donors are associated with high susceptibility to hypoxic damage. Optimization of organ preservation methods may ameliorate hypoxic damage. The traditional cold storage (CS) method of pancreas preservation lead to hypoxia of the pancreatic tissue.We hypothesize that oxygenated hypothermic machine perfusion (HMP) of the pancreas results in less hypoxia, resulting in a better quality of the isolated pancreatic islets. Method(s): In a pilot study 5 pancreata from DCD donors (age 53+/-15,8, BMI 24,6+/-4,2, CIT 8:30+/-3:30 hrs) were procured and transported in HTK/UWsolution. Upon arrival in the islet laboratory the pancreata were split transversally, resulting in a head and tail section. One section was preserved on ice (CS), the other was subjected to 3 hours of additional arterio-venous HMP. After prolonged CS and HMP islets were isolated separately. Pairwise, HMP tissue samples were compared with the CS tissue samples with regard to edema, oxygenation, amylase, lipase, LDH, gene expression and ATP content, islet isolation parameters (tissue retrieval, islet yield and purity) and islet quality parameters (gene expression, islet survival, glucose-induced insulin secretion). Result(s): No significant changes were observed in islet yield, survival or function. In the HMP perfusate increasing concentrations of amylase, lipase and LDH were detected, in line with the expected washout. Interstitial pO2 increased strongly from non-detectable levels to 80-90%. In general, mRNA expression was elevated in HMP tissue as compared to CS. The change in tissue ATP content after three hours HMP increased significantly. Conclusion(s): Three hours of oxygenated HMP of the pancreas increases oxygenation status but does not increase islet yield, function or viability. Earlier initiation and/or longer duration of HMP may alter these results.

14th World Congress of the International Pancreas and Islet Transplantation Association, IPITA 2013. Monterey, CA United States

Ferrer, J., et al. (2021). "Pancreas transplantation from donors declared death by circulatory criteria: Initial experience in Spain." Transplant International **34(SUPPL 1)**: 173-174.

Background: In the face of the shortage of organs for transplantation, the transplant community is increasingly considering controlled donation after the determination of death by circulatory criteria (cDCDD). There is a scarcity of studies concerning the use of Normothermic Regional Perfusion (NRP), an in situ preservation strategy well established in Spain. Aim(s): To report on the Spanish experience on the outcomes obtained from cDCDD donors. Method(s): Data from the Spanish National Transplant Organization database and from transplant centers were retrospectively analyzed (2015- 2020). Result(s): During the study period, 471 pancreas transplants were performed, including 20 combined kidney-pancreas transplants from cDCDD donors. Of these, NRP was used in 18 procedures, all with ante-mortem cannulation, and rapid recovery (RR) in 2 cases. The median donor age was 33 years, 65% were male. The median total warm ischemia time (WIT) and the functional WIT were 19 (13.2-23.7) min. and 10 (7-15.5) min., respectively. Postmortem NRP was run for a 113.5 (91.5-134.5) min. The median pancreas cold ischemia was 412.5 (330- 636.7) min. The pancreatic graft function was optimal in all cases except for three (NRP cases), for which the cause was primary nonfunction in two (one of them requiring transplantectomy of pancreatic and kidney grafts) and cardiogenic and septic shock secondary to pancreatic fistula for a fatal case. Seven patients presented with delayed kidney graft function, with five cases requiring dialysis. Pancreas related surgical complications were present in 70% cases, haemorrhage being the most common. After a median follow-up of 13.6 (5.6-36.4) months, 5-year pancreas graft survival was 85% for the whole series, kidney graft survival was 90% for and patient survival was 94.7%. Conclusion(s): To date, this is the largest series describing the use of postmortem NRP in cDCDD pancreas transplantation, displaying competitive results in terms of graft/patient survival.

20th Biennial European Society for Organ Transplantation Congress, ESOT 2021. Milan Italy

Ferrer-Fabrega, J., et al. (2021). "Controlled donation after circulatory death pancreas transplantation in Spain. Initial experience." American Journal of Transplantation **21(SUPPL 4)**: 841.

Purpose: To report on the Spanish Pancreas Transplantation experience in controlled donation after circulatory death (cDCD) by analyzing the normothermic regional perfusion (NRP) and super-rapid recovery Methods: Data from January 2015 to December 2020 were analyzed regarding pancreas transplantations using cDCD donors Results: Some 471 pancreas transplants were performed, 20 being from cDCD donors.NRP was used in 18 pancreases.The median functional WIT was 10 (7-15.5) min. NRP was run for 113.5 (91.5-134.5) minutes. Pancreas cold ischemia was 412.5 (330-636.7) minutes. Surgical complications were present in 70% of cases.Two patients of the NRP group presented with primary pancreas graft non-function. After a median follow-up of 13.6 (5.6-36.4) months, the 1 and 5-year overall pancreas survival rate was 85% for the whole series, 83.3% for NRP group and 100% for SSR group.Overall 1 and 5-year patient survival was 94.7%, with no group differences Conclusion(s): To date, this is the largest series describing the use of postmortem NRP in cDCD pancreas transplantation, providing competitive results in terms of graft/patient survival.

American Transplant Congress, ATC 2021. Virtual

Friend, P. J., et al. (2013). "Normothermic perfusion of discarded human pancreases." Transplant International **2)**: 159.

Background: Pancreas transplantation (solid organ or islets) is the most effective treatment for patients with progressive complications of diabetes, or patients with labile blood sugar control and hypoglycaemia unawareness. Increasing demand for transplants requires the use of more marginal donor organs, including those from DCD donors. However, the deleterious effects of cold ischaemia affect the viability of the organ and are exacerbated by prior injury. Currently there is no validated means to test the viability of the organ before transplantation. There is accumulating evidence of the benefits of a more physiological approach using continuous perfusion of an oxygenated, blood-based perfusate at normal body temperature. We describe here the feasibility of normothermic ex-vivo perfusion of human pancreases. Method(s): Human pancreases that were turned down by all transplant units have been utilised. A cardiopulmonary circuit consisting of oxygenator, heat exchanger, centrifugal pump, reservoir, flow probes and gate clamp is primed with time-expired packed red cells and the temperature maintained at 380C. The arterial inflow of the pancreas is cannulated and venous outflow collected and recirculated. Effluent from the duodenal segment is collected and measured. Result(s): Using this circuit, it is possible to achieve stable normal arterial

pressures and flows. During preservation, physiological flows and pressures are maintained in the splenic artery and the superior mesenteric artery by controlling the pump head speed and adjusting the proportional pinch valve on the bypass circuit. Discussion(s): This is the first demonstration of successful normothermic perfusion of the human pancreas. This has the potential to increase the viability, assessment and safety of donor organs in this expanding field.

16th Congress of the European Society for Organ Transplantation, ESOT. Vienna Austria

Kumar, R., et al. (2016). "An ex-vivo normothermic porcine pancreas physiological model and implications for islet cell transplant." HPB **2)**: e823.

Aims: Total pancreatectomy with autologous islet transplantation is a recognised treatment for chronic pancreatitis. Its success depends in large part on the islet yield following isolation. We established an ex-vivo normothermic porcine pancreas perfusion model which is physiological and facilitates detailed study of both exocrine and endocrine function. This model can be used to study the early phase of islet isolation. Method(s): Ten porcine pancreata were harvested and normothermically perfused with autologous blood. Serological parameters measured were: blood gas composition, routine biochemistry, glucose, insulin and glucagon levels. The volume of pancreatic juice secreted was also collected pre and post-secretin challenge. Result(s): All pancreata were perfused for a median of 3 hours (range 2 - 4 hours) with a baseline perfusion pressure of 50mmHg, flow rate of 0.17 L.min-1 (range 0.14 - 0.33 L.min-1) and a blood lactate of 4.2mM (range 2.7mM - 8.4mM). All pancreata demonstrated cellular viability with evidence of oxygen consumption (arterio-venous O2 differential concentration) and produced pancreatic juice at a median rate of 3mL.hr-1 (range 2 - 17mL.hr-1). Four pancreata demonstrated an increased insulin secretion following a glucose challenge. Conclusion(s): This model avoids the use of live animals and can be used to investigate the islet yield in a porcine model following a period of machine perfusion. The physiological behaviour of this ex-vivo perfused pancreas model will allow the changes that occur in harvested pancreata and the potential effect they have on islet isolation, yield and viability to be studied in detail.

11th International Congress of the European-African Hepato-Pancreato-Biliary Association. Manchester United Kingdom

Kumar, S., et al. (2016). "Novel islet culture technology significantly improves islet function and practical advantage of good transportation of islets." Diabetic Medicine **1)**: 42.

Aims: Pancreatic islet transplantation has emerged as a potential cure for Type 1 diabetes. Human pancreatic islets are routinely cultured in static plastic tissue culture flasks to ship from the isolation to the transplantation facility, which results in huge loss of islet mass and in vivo islet functionality. Our aim was to study viability and functionality of pseudo-islets cultured in a batch rotary cell culture system (B-RCCS) and a perfusion rotary cell culture system (P-RCCS). Method(s): Min6 beta cells were cultured as monolayers and then reconfigured into pseudo-islet clusters (PIs) in static dishes and BRCCS and P-RCCS bioreactors. Morphology of pseudo-islet was analysed by confocal, SEM, light microscopy. Specific functional markers, PDX1, insulin and ECM proteins, were analysed by immunocytochemistry analysis and western blotting. Pseudo-islet viability was assessed by LDH, and HPI assay. PIs were subsequently allowed to recover under static or bioreactor cell culture conditions. Result(s): Pseudo-islet cultured in batch and perfusion RCCS bioreactors showed extended viability compared to non-adherent cell culture dishes. Insulin gene expression, secretion and content were markedly increased in both batch and perfusion RCCS bioreactors compared to static cultures (dishes and plastic flask). Morphology analysis showed that PIs generated in batch and perfusion RCCS bioreactors have smoother surface and higher ECM. Fibronectin and laminin V expression was significantly increased under bioreactor compared with static conditions. Conclusion(s): Batch and perfusion RCCS bioreactors tested in this study combine an excellent method for restoration, preservation and enhancement of islet viability, functionality and the practical advantage of good transportation of islets.

Diabetes UK Professional Conference 2016. Glasgow United Kingdom.

Leemkuil, M., et al. (2015). "Preliminary results of isolated islets after hypothermic machine perfusion of human donor pancreata." Transplant International **4)**: 178.

Background: Islet transplantation is an effective treatment option for patients with type I diabetes mellitus. Due to the persistent organ shortage, pancreata from marginal donors are more frequently used for islet

transplantation. The conventional preservation method cold storage (CS), inadequately prevents ischemia prior to islet isolation. It is hypothesized that hypothermic machine perfusion (HMP) improves the quality of pancreata for islet isolation. Methods. Human donor pancreata unsuitable for clinical transplantation were connected to our modified dual arterial kidney machine system after an initial period of cold ischemia during transport. Islets of Langerhans were isolated after 6 h of oxygenated HMP. Islet viability was analyzed by fluorescein diacetate (FDA) and propidium iodide (PI) staining. Islet viability was analyzed by performing a glucose stimulated insulin secretion (GSIS) test. After 3 or 4 days of culture, the islets were transplanted under the kidney capsule in STZ-induced diabetic mice (3000 IEQ/mouse). Blood glucose levels were monitored every other day for 28 days. Result(s): So far, three DCD pancreases have been included in this study. The preliminary data show an IEQ after isolation of 336957 +/- 91019 and an IEQ/ gram of 4476 +/- 1785. Islet viability at day 1 was 98 +/- 0% and at day 3 92.6 +/- 4.6%. Static GSIS at day 1 showed an induction fold of 2.04 +/- 0.42 and at day 3 2.57 +/- 1.52. After transplantation, normoglycemia was achieved in 6 out of 10 mice. In 2 out of 10 mice, blood glucose levels normalized initially, but the graft function declined 14 days after transplantation. 2 out of 10 mice were terminated because of postoperative complications and early graft failure. Conclusion(s): The preliminary data suggests that functional, viable islets can be readily isolated from pancreata after HMP. Inclusion of additional pancreata in the HMP preserved group and in a CS preserved control group is ongoing.

17th Congress of the European Society for Organ Transplantation, ESOT. Brussels Belgium.

Leemkuil, M., et al. (2015). "Hypothermic machine perfusion improves the quality of marginal donor pancreata." Xenotransplantation **1)**: S140-S141.

Introduction: Pancreas or islet transplantation is considered the most effective treatment for patients with type 1 diabetes mellitus. Due to the persistent organ shortage, pancreata from marginal donors are more frequently used for transplantation or islet isolation. These grafts are more vulnerable to ischemic damage. The traditional preservation method, cold storage (CS), might not be sufficient to completely prevent this damage. In kidneys and livers, hypothermic machine perfusion (HMP) is shown to be superior to CS, especially for the more marginal donor organs. The aim of this project was to study the potential beneficial effect of HMP on the pancreas. Method(s): 8 human pancreata (4 from Donation after Circulatory Death (DCD) donors and 4 from Donation after Brain Death (DBD) donors) were preserved by HMP and 8 (divided in two similar groups) by CS. HMP was performed for 6 h with oxygenated Belzer Machine Perfusion Solution with a dual perfusion system to allow perfusion via the mesenteric superior artery and the splenic artery separately. The perfusion pressure was set to 25 mmHg. Tissue biopsies and samples of the preservation fluid were collected at baseline and after 6 h of preservation for histology, tissue ATP measurement and enzyme level analysis. In addition, pancreas weight was measured before and after preservation as a measurement of edema formation. Result(s): No significant difference in pancreas weight increase was seen after preservation by CS or HMP. Histological analysis of the tissue showed an intact structure of the pancreatic tissue after HMP. During HMP, amylase, lipase and LDH levels in the preservation fluid increased reaching a plateau after 4 h. No increase was found in enzyme levels after CS. At baseline, adenosine triphosphate (ATP) content in the DCD group (8.2 + - 5.6 lmol/gram protein) was significantly lower than in the DBD group (43.5 + - 16.2 lmol/gram protein). During CS, ATP content decreased in both groups. In contrast, in the HMP preserved pancreata, ATP content increased to 47.9 + - 25 (DCD) and 136.4 + - 144 (DBD) lmol/gram protein. In the DCD group, the ATP content after HMP was significant higher compared to CS and it was equivalent to the ATP content at baseline in the DBD group. Conclusion(s): HMP improves the viability of donor pancreata, demonstrated by increased ATP levels. In addition, we postulate that pancreas quality is enhanced by washout of degrading enzymes. The viability of marginal pancreata, such as DCD pancreata, could reach that of DBD pancreata using HMP. Enlargement of the pancreas donor pool is therefore likely.

IPITA-IXA-CTS 2015 Joint Congress. Melbourne, VIC Australia

Matsumoto, S., et al. (2010). "ET-kyoto solution for ductal injection and islet purification improved islet isolation for clinical allo-islet transplantation." American Journal of Transplantation **4)**: 499.

Background: Necessity of multiple donors for achieving insulin-free status is one of the issues for allo islet transplantation. Increasing of islet yield should make it possible to achieve single donor islet transplantation. Aim(s): The purpose of this study is to examine whether the ET-Kyoto solution for ductalpreservation and

purification can improve islet isolation results. Method(s): From April2006 to November 2009, 21 clinicalgrade pancreata were used for this study. Allpancreata were preserved by the two-layer method less than 6 hours. The pancreata were categorized into three groups, the standard group (Standard, N=7), the ductalinjection group (DI, N=7) and the ductalinjection plus new purification method group (DP, N=7) based on islet isolation methods. Pancreatic ductalinjections were performed in the DIand DP group using the ET-Kyoto solution. Islets were isolated by the Ricordimethod in allgroups and purified using Ficollin the standard group and DIgroup. In the DP group islets were purified using the ETKyoto solution and iodixanol. Islets were assessed by the Edmonton protocol. Result(s): There were no significant differences among three groups in donor BMI, pancreas weight, age, and maximum glucose and amylase levels. The finalislet yields in the controlgroup was 254,836+/-89,649 IE, in the DIgroup 562,373 +/- 62,205 IE, and in the DP group was 666,272+/-558,253 IE (Fig 1A). The recovery rate after purification in the standard group was 41.6+/-10.0%, in the DIgroup was 63.9+/-6.5%, and in the DP group was 85.2+/-4.1% (Fig 1B). ET-Kyoto solution significantly improved islet yield and efficacy of islet purification. Three out of seven preparations in controlgroup, and allin the DIand DP groups met transplantation criteria. (Figure presented) Conclusion(s): ET-Kyoto for ductalinjection and islet purification significantly increased islet yield from clinicalgrade pancreata. This method would help to achieve single donor islet transplantation.

American Transplant Congress 2010, ATC 2010. San Diego, CA United States.

Ogbemudia AE, Dengu F, Hakim G, El-Gilani F, Mulvey J, Prudhomme T, Lo Faro ML, Rozenberg K, et al (2021). Transplant International 34 (suppl 1):p175.

Background: Static cold storage (SCS) is the standard method for pancreas preservation but does not facilitate objective organ assessment prior to transplantation. Normothermic machine perfusion (NMP) has been used to test other solid organs' function and viability in transplantation settings. Our aim was to develop a NMP protocol specific for pancreases and then investigate its potential as an organ assessment strategy. Methods: 15 porcine pancreases were procured in conditions replicating donation after circulatory death with warm ischaemia time of 25 minutes. After 3 hours of static cold storage (SCS), the pancreases were divided into 4 experimental groups (figure 1).

1) 2 pancreases in the feasibility group, which after backbench preparation were placed directly on NMP in order to develop the NMP protocol

2) 5 pancreases in the HMP Institut Georges Lopez (IGL2) group

3) 4 pancreases in the HMP University of Wisconsin MPS (UW) group

The IGL2 and UW groups above received HMP supplemented with 21% oxygen.

4) 4 pancreases in the control (SCS) group were stored at 4 degrees in UW as is the standard practice in the United Kingdom.

The latter three experimental groups (IGL2, UW and SCS) received their intervention for 6 hours prior to assessment on NMP for 1 hour. The NMP protocol used autologous, leucodepleted blood delivered at a mean arterial pressure of 40mmHg with a temperature of 37°C. At timed intervals during HMP and NMP, perfusate and tissue samples were collected for analysis and perfusion parameters were recorded.

Results: During hypothermic preservation, lactate and LDH was noted to be highest in the SCS group. During NMP assessment the SCS group displayed a worse resistance, poorer flows and worse macroscopic appearances compared to both HMP groups. Tissue wet to dry ratio technique was used as a surrogate for oedema assessment and the highest weight gain observed in the SCS compared to both the HMP groups.

Conclusions: Our work although with small numbers suggest NMP of whole pancreases is feasible after cold preservation and is potentially useful as an assessment strategy. Furthermore, it appears to demonstrate that oxygenated HMP may be beneficial for pancreas preservation compared to SCS.

20th Biennial European Society for Organ Transplantation Congress, ESOT 2021. Milan, Italy

Figure 1 Study schema showing the experimental groups

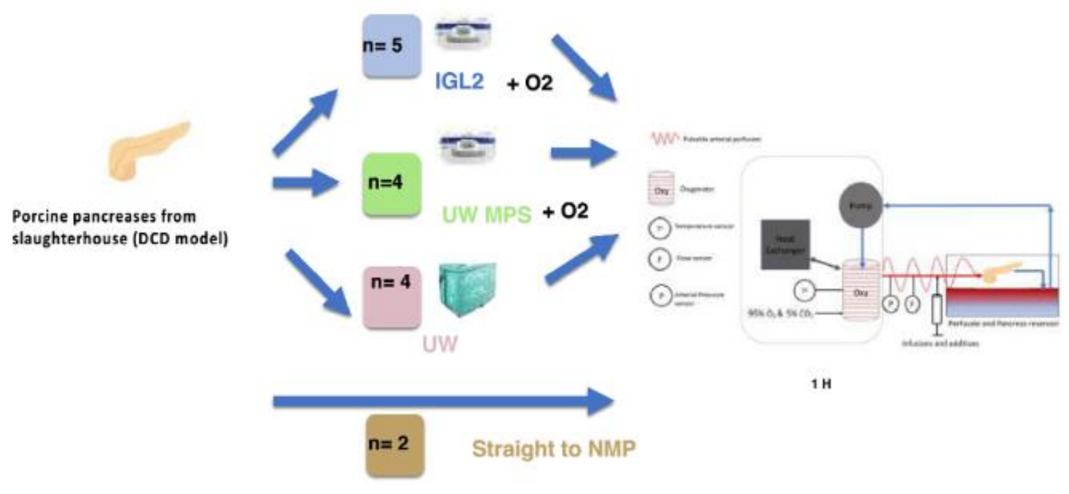

Papas, K. K. (2010). "Oxygen persufflation of the pancreas prior to islet isolation." Cryobiology **61(3)**: 382.

Islet transplantation (ITx) is emerging as an attractive treatment option for qualified patients with type-1 diabetes. The success of ITx (insulin independence) is strongly influenced by the number of transplanted viable islet equivalents (IE) per kilogram recipient body weight. Reduction in islet viability occurs at multiple pancreas and islet processing steps, especially those exposing islets to hypoxia. Islets express very low levels of LDHa, the enzyme needed to generate energy with anaerobic glycolysis and are therefore particularly sensitive to hypoxia. Current static cold preservation protocols, including the state-of-the-art two layer method (TLM), do not prevent exposure of the majority of the human pancreas to hypoxia or even anoxia. This highlights the need for the development of improved methods for pancreas oxygenation during preservation. In this context we explore arterial oxygen persufflation (PSF), as an effective method of oxygen delivery to porcine and human pancreata, and report on its ability to improve pancreas preservation and ultimately islet isolation outcomes relative to the TLM. Human pancreata and paired lobes from porcine-pancreata were preserved up to 24 h with PSF or TLM. PSF was performed by pumping 20 cc/min 40%-oxygen humidified gas to the superior mesenteric artery and celiac trunk averaging pressures of 10- 20 mmHg. Homogeneity of PSF was assessed by NMR imaging, visualizing negative contrast associated with the presence of gas in the vasculature. ATP and the ATP to inorganic phosphate ratio (ATP:Pi) were non-invasively measured by 31P-NMR spectroscopy at 1.5 T. Porcine islets were isolated following 6- or 24-hour preservation, cultured for 2 days, and assessed by islet counts, morphology, DNA content, oxygen consumption rate normalized to DNA (OCR/DNA), and the diabetic-nude mouse bioassay (2000 IE transplanted per mouse). NMR imaging demonstrated that pancreatic tissue was homogeneously persufflated. ATP was undetectable in porcine or human pancreata stored on TLM, while substantial levels of ATP were detected when porcine and human pancreata were persufflated. ATP:Pi remained elevated throughout PSF, with minimal-gradual decline over time observed. In some organs, PSF was stopped resulting in an immediate decline in ATP:Pi until ATP levels were undetectable. Restoration of PSF partially restored ATP:Pi. On average the persufflated lobes resulted in higher islet yield and post-culture recovery than paired lobes stored with the TLM, but the differences were not statistically significant. Islets isolated from persufflated porcine lobes exhibited higher morphology score ($P < 0.05$) and viability (OCR/DNA, $P < 0.05$) when compared with those isolated from paired TLM lobes. Importantly, islet yield, viability, post culture recovery and cure rates in the diabetic-nude mouse bioassay with islets isolated from lobes persufflated for 24 h were similar to those obtained with islets isolated from lobes shortly after procurement. It is concluded that PSF increases ATP levels and viable islet yield relative to TLM and may extend pancreas preservation time to 24 h without compromising viable islet yield. PSF can potentially improve the logistics and cost-effectiveness of organ allocation and preservation, while enhancing islet isolation and

transplantation outcomes.

47th Annual Meeting of the Society for Cryobiology. Bristol United Kingdom.

Papas, K. K., et al. (2015). "Pancreas oxygen gas perfusion (persufflation) during preservation improves clinical islet isolation yields and success rates." Xenotransplantation **1)**: S192-S193.

Human islet allo-transplantation is emerging as a promising treatment option for type-1 diabetes with current success rates at experienced centers approaching those of whole pancreas transplantation in terms of 5 year insulin independence rates. However, to achieve these results islets from more than one pancreas are often needed. In addition, typically more than half of the pancreata procured and processed to islets for clinical transplantation do not produce sufficient islets to meet the minimal islet dose required and therefore are not transplanted [1]. There are various reasons contributing to the reduction in viable islet yield per pancreas. Suboptimal pancreas preservation (specifically oxygen starvation) is recognized as an important factor contributing to this problem. Currently utilized methods of pancreas preservation based on static-cold-storage (SCS) including the two-layer method (TLM), are incapable of oxygenating human sized organs as they rely solely on diffusion and pancreas preservation times are typically kept to below 8-12 h in an attempt to reduce CIT induced damage. Enhanced oxygenation is believed to improve isolation outcomes and methods that can accomplish it are under investigation. One such method, oxygen gas perfusion (persufflation, PSF), delivers humidified gas through the native vasculature and may be able to improve isolation outcomes or extend the effective duration of preservation for islet isolation. We have previously shown using a porcine and human pancreas research model that PSF can extend preservation time to 24 h without compromising viable islet yield relative to that obtained from minimally preserved pancreata, and that PSF resulted in superior islet yields relative to 24 and even 8-12 h SCS with TLM. The objective of this pilot study was to establish the PSF technique in a clinical pancreas procurement and islet isolation setting (Clinical islet Transplantation Program at Edmonton) and to investigate whether PSF could improve clinical islet isolation yields and therefore islet isolation success rate relative to the current state-of-the-art. Five human pancreata were procured according to standard procedures and were preserved via persuffation following a period of static cold storage. PSF was performed by providing 40%-humidified oxygen gas via the arterial vasculature at 10-25 mmHg, utilizing an electrochemical oxygen concentrator (Giner Inc; Newton, MA). Results demonstrated that 100% of persufflated pancreata (as opposed to 42% with the current state-of-the-art [1]) resulted in transplantable clinical islet preparations. Isolation outcomes tended to be better the lower the CIT prior to the onset persufflation. Ongoing efforts focus on increasing the number of persufflated pancreata prior to isolation and to obtaining results from pancreata persufflated as close to the procurement as possible so as to confirm the findings of this pilot study. We conclude that persufflation has the potential to dramatically improve clinical pancreas preservation and islet isolation outcomes and if confirmed the implementation of this technique in the clinical setting will increase the availability, affordability, and potentially the success of this procedure.

IPITA-IXA-CTS 2015 Joint Congress. Melbourne, VIC Australia

Prudhomme, T., et al. (2019). "Ex-situ hypothermic perfusion of non-human primates pancreas: An experimental model." Transplant International **32(Supplement 2)**: 229.

Pancreas transplantation remains only definitive treatment for unstable insulin dependent diabetes mellitus. Static cold storage of pancreas transplants remained conservation reference method. The main objective was to evaluate feasibility of hypothermic perfusion of non-human primates pancreases for future potential organ transplantation. The secondary objective of this study was to evaluate quality of non-human primates pancreases after 24-h of hypothermic perfusion. Seven non-human primates pancreases, arised from baboons used in other study of the laboratory, approved by French Research Ministry, were used. Two groups, comparing static cold storage (SCS) and hypothermic perfusion (HP) of baboons pancreas, were defined: control group (CG) (n = 2) where pancreases were preserved under conventional SCS for 24-h and perfusion group (PG) (n = 5) where pancreases were perfused during 24-h at different systolic pressure (15 mmHg (n = 3), 20 mmHg (n = 1), 25 mmHg (n = 1)). Pancreatic resistance index was continuously monitored; pancreas and duodenum histology and immunohistochemical was evaluated every 6-h. In CG, after 6-h of static cold storage, focal congestion appeared in islets in one of the pancreases. After 24-h of SCS, ischemic necrosis and multifocal congestion occurred in both pancreas. In PG, at 15 mmHg perfusion pressure, focal and multifocal congestion were present in islets after 6-h of perfusion. After 24-h of perfusion, multifocal

necrosis in exocrine tissue and multifocal congestion in islets appeared in all pancreases. At 20 mmHg perfusion pressure, no ischemic necrosis was found in islets after 6-h of HP. After 12-h and 24-h of perfusion, focal congestion appeared in islets. At 25 mmHg perfusion pressure, ischemic necrosis of duodenum, and not of the pancreas, appeared after 24-h. Focal congestion of islets appeared after 12-h. Experimental non-human primates pancreas hypothermic perfusion are feasible. Compared to SCS, HP is not deleterious for pancreas.

19th Congress of the European Society for Organ Transplantation. Copenhagen Denmark

Prudhomme, T., et al. (2020). "EX-situ hypothermic perfusion of non-human primate pancreas: A feasibility study." Transplant International **33(Supplement 1)**: 16.

Background: The objective was to evaluate feasibility of hypothermic perfusion (HP) of non-human primatespancreases for potential organ transplantation. Method(s): Seven baboon pancreases were tested, animals were included in a study approved by the French Research Ministry of Health. Two groups were compared: the control group (n = 2) was preserved using conventional static cold storage (SCS) for 24-h and the perfusion group (n = 5) used HP for 24-h, with 3 different perfusion pressures (PP): 15 (n = 3), 20 (n = 1) and 25 mmHg (n = 1). Result(s): In control group, focal congestion of islets was observed after 6-h. At 24-h, ischemic necrosis and multifocal congestion occurred. In HP group, at 15 mmHg PP, multifocal congestion of islets was present at 24-h. At 20 mmHg PP, no ischemic necrosis was found after 6-h. At 12-h and 24-h, focal congestion of islets was seen. At 25 mmHg PP, focal congestion of islets appeared after 12-h. Immunostaining for insulin, glucagon and somatostatin was normal and similar in controls and perfused pancreas transplants even after 24-h. Conclusion(s): HP of non-human primate pancreas is feasible and not deleterious as far as 24-h compared to SCS. Systolic perfusion pressure between 15-25 mmHg did not cause any pathological injury of the tested organs.

19th Annual Congress of the French Speaking Transplantation Society. Bordeaux France

Reddy, M. S., et al. (2010). "Protective post conditioning effect of portal venous oxygen persufflation in pancreas grafts damaged by significant warm ischemia." Transplantation **1)**: 1012.

Introduction: The success of islet transplantation depends on the number of islets transplanted and the severity of early islet loss due to ischemia reperfusion injury. We have previously reported improved islet yields in donation after cardiac death (DCD) donors pancreases preserved with venous oxygen persufflation. We have now investigated the effect of preservation with venous oxygen persufflation on markers of ischemia reperfusion injury and the recruitment of protective inflammatory pathways in a non-heart-beating-donor rat pancreas preservation model. Method(s): Pancreases were retrieved from male Wistar rats with warm ischemia duration of 35 minutes. They were randomised to preservation by either static cold storage (CS, n=6) or venous oxygen persufflation through the portal vein (OP, n=8) for 16 hours. Pancreases then underwent one hour of warm oxygenated perfusion with Krebs Henseleit solution. Amylase, lipase and glycerol levels in venous effluent were compared between the two groups at timed intervals during reperfusion. Tissue was collected at the end of reperfusion for estimation of lipid peroxidation using Thio-barbituric acid reactive substances (TBARS)assay and RNA extraction for microarray analysis. Microarray data was analysed using GenMAPP to compare gene expression in the two preservation groups with particular reference to pro-survival inflammatory pathways i.e. Protein kinase B (PKB/Akt), Extracellular signal related kinase (ERK/MAPK) and Nuclear factor kappabeta (NFkappabeta) pathways. Control group pancreatic tissue with no warm ischemia was included (n=3) for estimation of lipid peroxidation and for RNA microarray analysis. Result(s): Duodenal peristalsis was seen in 3 pancreases of the OP group but none in the CS group. There was no significant difference in the levels of amylase, lipase and glycerol levels in the portal venous effluents of two preservation groups. TBARS levels were significantly higher in the DCD pancreases as compared to the control group. There was no difference in TBARS levels between the two DCD groups. However microarray analysis revealed an over-representation of the promoters of the PI3K-AKT, NFkappabeta and the ERK/MAPK pathways in the venous oxygen persufflation group. Genes related to cellular respiration, cellular transport and biosynthesis were over-expressed in the venous oxygen persufflation group. Conclusion(s): Venous oxygen persufflation of DCD pancreases is associated with a significant recruitment of pro-survival inflammatory pathways and pathways related to increased metabolic activity. We propose that venous oxygen persufflation produces a protective post-conditioning effect in pancreases damaged by a period of warm ischemia. Further studies into its relevance to clinical pancreas and

islet transplantation are required.

23rd International Congress of the Transplantation Society, TTS 2010. Vancouver, BC Canada.

Richards, J., et al. (2021). "Comparable outcomes for circulatory death and brain-stem death pancreas transplantation irrespective of the use of normothermic regional perfusion." Transplant International **34(SUPPL 1)**: 28.

Background: Simultaneous pancreas and kidney transplantation is the optimum treatment for patients with type 1 diabetes and renal failure, providing survival benefit over deceased donor kidney transplant alone. Method(s): We performed a retrospective analysis of prospectively collected outcomes of the first 10 years of our Donation after Circulatory Death (DCD) pancreas transplant program, including DCD donors undergoing Normothermic Regional Perfusion (NRP). Result(s): 211 patients (139 donation after brainstem death (DBD), 72 DCD (59 conventional DCD and 13 NRP retrieval)) were included in the study. Patient survival at 1, 3, 5, and 10 years was 99.0%, 96.6%, 93.4% and 84.3%, respectively, with no significant difference in patient survival between those recipients receiving grafts from DBD or conventional DCD donors. Death-censored pancreas and kidney graft survival at 5 years was 83.9% and 93.2%, respectively, with no significant difference between DCD and DBD cohorts. For those receiving a DCD graft, patient survival, and pancreas and kidney transplant outcomes were comparable, irrespective of whether the organs were procured conventionally or following NRP. Conclusions In conclusion, utilisation of DCD pancreases is a safe approach to expanding the donor pool with equivalent results to DBD transplantation. Pancreas transplantation following NRP appears to be feasible, but warrants further study.

20th Biennial European Society for Organ Transplantation Congress, ESOT 2021. Milan Italy

Richards, J., et al. (2021). "The impact of normothermic regional perfusion on simultaneous kidney and pancreas transplantation." Transplant International **34(SUPPL 1)**: 205.

Background: Simultaneous pancreas and kidney (SPK) transplantation is the optimum treatment for patients with type 1 diabetes and renal failure and provides survival benefit over deceased donor kidney transplantation alone. Donation after circulatory death (DCD) SPK transplantation has equivalent long-term results to organs from brainstem dead donors (DBD), but is associated with increased rates of ischemia reperfusion injury and delayed graft function. Normothermic Regional Perfusion (NRP) has emerged as a promising technique to minimise or reverse the additional ischemic insult associated with conventional DCD (sDCD) donation by placing the donor on a modified extra-corporeal membrane oxygenator circuit. To date, little has been published on the outcomes of pancreas transplantation following NRP beyond case reports. Method(s): We performed a retrospective analysis of prospectively collected outcomes our DCD pancreas transplant program and comparing the outcomes of recipients receiving SPK grafts following sDCD and NRP procurement. Result(s): 266 patients were included in the study (171 DBD, 77 sDCD, 18 NRP). There was no significant difference between cohorts in terms of serum creatinine, eGFR at 1 year or HbA1c. There were no significant differences in the potential biochemical markers of graft pancreatitis (CRP, White Blood Count, Neutrophil Count, Albumin, Platelet Count, Amylase, Lipase). There was a significantly lower rate of renal delayed graft function (DGF) in the DBD 41/171 (24.0%) and NRP cohorts 5/18 (27.8%) compared to sDCD cohort 42/77 (54.5%). No differences were seen in the rates of pancreas DGF in DBD 4/171 (2.3%), sDCD cohort 3/77 (3.9%) and NRP cohorts 0/18 (0%). Conclusion(s): While there is increasingly strong evidence showing benefit in the setting of liver transplantation, the benefit in the setting of pancreas transplantation is less clear. We believe this paper represents the largest single centre DCD and NRP series in the setting of SPK presented to date and in it demonstrated that.

20th Biennial European Society for Organ Transplantation Congress, ESOT 2021. Milan Italy

Scott, I. W. E., et al. (2010). "Oxygen persufflation increases pancreatic atp levels and viable islet yield following 24 hours preservation compared with the two-layer method (Tlm)." Transplantation **1)**: 229.

Introduction: Islet transplantation (ITx) is a promising treatment for type-1 diabetes. The success rate of ITx is highly dependent on the number of transplanted viable islet equivalents per recipient body weight. The current state-of-the-art in pancreas preservation, the Two-Layer Method (TLM), is suboptimal and can oxygenate only a small portion of large (i.e., porcine, human) pancreata. The stress of anoxic preservation reduces viable islet yields, especially at preservation times longer than 6-8 hours. This necessitates the development of improved methods of oxygenation during preservation prior to islet isolation. Here, we report on arterial oxygen persufflation (PSF), an alternative method of oxygen delivery during preservation.

Method(s): Human pancreata and paired porcine pancreatic lobes were preserved up to 24 hours with PSF or TLM. PSF was performed by pumping 20 cc/min 40%-oxygen humidified gas to the superior mesenteric artery and celiac trunk averaging pressures of 10-20 mmHg. Homogeneity of PSF was assessed by magnetic resonance imaging (MRI), visualizing negative contrast associated with gas in the vasculature. ATP to inorganic phosphate ratio (ATP:Pi) was non-invasively measured by 31P-NMR spectroscopy at 1.5 T. Porcine islets were isolated following 6- or 24-hour preservation, cultured for 2 days, and assessed by counts, morphology, DNA content, oxygen consumption rate normalized to DNA (OCR/DNA), and the diabetic nude mouse bioassay (2,000 islet equivalents per mouse). Result(s): MRI demonstrated that pancreatic tissue was homogeneously persufflated. ATP:Pi remained elevated throughout PSF, with minimal gradual decline observed. In some organs, PSF was stopped resulting in an immediate decline in ATP:Pi until ATP levels were undetectable. Restoration of PSF partially restored ATP:Pi. Persufflated porcine lobes exhibited elevated ATP:Pi compared with paired TLM lobes, higher islet morphology score (P<0.05), islet yield, viability (OCR/DNA, P<0.05), and post-culture recovery. Persufflated islets also exhibited similar cure rates in diabetic nude mice compared with historical data from freshly procured pancreata. (Figure presented).

23rd International Congress of the Transplantation Society, TTS 2010. Vancouver, BC Canada.

Scott, W. E., et al. (2010). "Pancreas oxygen persufflation increases ATP-levels and improves islet viability compared with two-layer method (TLM)." American Journal of Transplantation **1)**: 74.

Background: Islet transplantation is a promising treatment for type-1 diabetes. Many pancreata are exposed to >6-8 hours of cold ischemia reducing viable-islet yields. TLM's efficacy is disputed, since adequate diffusion-based oxygenation of large organs is impossible. Oxygen persufflation offers an alternative oxygenation method. Method(s): Human pancreata and paired porcine pancreatic lobes were preserved by persufflation or TLM. Persufflation was performed by pumping 20 cc/min 40%-oxygen humidified gas to the superior mesenteric artery and celiac trunk. ATP:P<inf>i</inf> was non-invasively determined by ³¹P-NMR spectroscopy at 1.5 T. Islets were isolated following 6-or 24-hour preservation and cultured. Result(s): Persufflated human organs showed elevated ATP:Pi that decreased when persufflation was stopped until AT P was undetectable. Restoring persufflation, partially restored ATP:P<inf>i</inf>. Persufflated porcine lobes exhibited elevated ATP:P<inf>i</inf> compared with paired TLM lobes, better morphology (P<0.05), and higher islet yields, superior viability (oxygen consumption rate/DNA, P<0.05), and post-culture recovery. Persufflated islets also exhibited high cure rates in diabetic nude mice compared with historical data. Conclusion(s): Persufflation homogeneously delivers oxygen throughout the organ improving tissue survival and viable-islet yields. Persufflation shows promise to extend preservation.

American Society of Transplant Surgeons 10th Annual State of the Art Winter Symposium. Fort Lauderdale, FL United States.

Taylor, M. J., et al. (2009). "Islet isolation from juvenile pig pancreas after 24 h hypothermic machine perfusion preservation: Effect of preservation solution and warm ischemia." Xenotransplantation **16(5)**: 320.

Objectives: Perfusion preservation technology has recently been reported to improve the recovery of islets after 24 h hypothermic machine perfusion (HMP) of porcine pancreas (Px) prior to islet isolation. This technology is now applied to juvenile pig Px, which is generally considered to be the species of choice for xenogeneic islet transplantation (Tx). Method(s): Pancreata in young (<6 m) Domestic Yorkshire pigs (25-30 kg) were surgically removed, either before or after 30 min warm ischemia time (WIT), and the superior mesenteric artery and celiac trunk were cannulated. Px were assigned to one of 6 preservation groups: Fresh controls: cold ischemia <1 h [G1, n =7], Static Cold Storage: flushed &stored in UW-Viaspan at 2-4degreeC for 24 h with no prior WIT [G2, n=9], HMP-perfused on a clinical LifePort machine at 4-6degreeC and low pressure (10 mmHg) for 24 h with either KPS1 solution (G3, n=7) or Unisol-UHK (G4, n=7). Additional treatment groups to evaluate the effects of prior WIT examined islet isolation after 30 min WIT in situ without [G5, n=6], or with subsequent 24 h HMP with KPS1 (G6, n=7). Islet isolation was then accomplished by ductal distension with liberase enzyme, normothermic digestion and density gradient purification. Standard assessment criteria were used including islet quantification, function using the glucose stimulated insulin secretion assay, and histology. Result(s): Perfusion induced glandular edema was G3=138 +/- 19%; G4=160 +/-16 and G6=127 +/- 22%. Islet yields (IEQ/g) were: G1=1425 +/- 610; G2=1002 +/- 262; G3=2242 +/- 449 (p < 0.05 vs G2); G4=1901 +/- 420 (p < 0.05 vs G2); G5=1756 +/- 329 and G6= 1396 +/- 243. Islet stimulation indices were equivalent between the groups and similar to controls (G1). A consistently more

uniform digestion of the perfused organs was observed, with greater separation of the tissue and less entrapped islets, higher islet yield and purity. Conclusion(s): HMP (24 h) using 2 alternative perfusates is well tolerated, even after 30 min WIT, leading to moderate edema but no loss of function of the harvested islets. The edema appears to aid digestion producing a greater yield and purity of islets compared with Px subjected to 24 h of static cold storage.
2009 Joint Meeting of the International Pancreas and Islet Transplant Association (IPITA) and the International Xenotransplantation Association (IXA). Venice Italy.

Witkowski, P., et al. (2005). "Two-layer method in short-term pancreas preservation for successful islet isolation." Transplantation Proceedings **37(8)**: 3398-3401.
Background. We sought to determine whether the two-layer method (TLM) offers advantages over UW storage solution for locally procured pancreata with cold ischemia time of <8 hours for successful islet isolation. Methods. From October 2003 through February 2005, 22 human pancreata were procured locally from cadaveric donors and preserved using UW solution (n = 11) or TLM (n = 11). Results. Donor characteristics were similar in the two groups, with no statistical difference. Cold ischemia time was 4.5 +/- 0.6 (2.5 to 8) hours in the UW and 5.1 +/- 0.5 (3 to 8) hours in TLM group (P > .05). Organs preserved with TLM were exposed to PFC for 4 +/- 0.5 (2 to 7.5) hours. After TLM preservation, 8 of 11 (72%) pancreata yielded >300,000 IEQ pancreatic islets, which met all criteria for clinical transplantation; after UW cold storage, only 3 of 11 isolations were equally successful (27%) (P < .05). Mean IEQ was higher in the TLM than in the UW group: 349,000 +/- 37,000 vs 277,800 +/- 34,000; IEQ/g was also higher at 5100 +/- 760 vs 3000 +/- 570, respectively (P < .05). Islet quality, characterized by purity, viability, and insulin SI, did not differ statistically in the two groups: 67 +/- 4 vs 74 +/- 4%, 87 +/- 2 vs 83 +/- 4%, and 4 +/- 0.7 vs 4.8 +/- 1, respectively (P > .05). Conclusions. The Two Layer Method for locally procured human pancreata with cold ischemia time lower than 8 hours offers significant advantage over UW cold storage increasing the pancreatic islet isolation yield and the isolation success rate.
http://dx.doi.org/10.1016/j.transproceed.2005.09.050
