# Supplementary material for: European Society for Organ Transplantation (ESOT) Consensus Statement on the Role of Pancreas Machine Perfusion to Increase the Donor Pool for Beta Cell Replacement Therapy
Source: Transpl Int. 2023 Jul 19;36:11374. doi: 10.3389/ti.2023.11374 (PMC10402633; doi:10.3389/ti.2023.11374)
Supplement: Supplementary file 2 [file DataSheet4.pdf]

Role of Pancreas Machine Perfusion to Increase the Donor Pool for beta cell replacement

Chair: Joana Ferrer
Secretary: Julien Branchereau
Evidence Review Coo. (YPT): Benoît Mesnard
Scientific Committee: Marten A Engelse, Trevor Reichman, Vassilios Papalois, Cinthia Drachenberg, Fabio Vistoli, Steve White, Paul Johnson, Henri G. D. Leuvenink, Ann Etohan Ogbemudia, Franka Messner, Jason Doppenberg

INTRODUCTION AND RATIONALE FOR CONSENSUS

Due the shortage of eligible deceased donors to support whole pancreas transplantation more extended criteria donors (ECD) are being considered, with characteristics of increased age, BMI or donation after circulatory death (DCD). The impact of this donor organ scarcity extends to islet transplantation with islet transplant and isolation units also considering ECD pancreases.
The advent of Machine Perfusion (MP) as a method of organ preservation and assessment has proven particularly advantageous compared to static cold storage for high-risk kidney and liver donor organs. The positive impact of MP technologies has therefore inspired research in the field of pancreas and islet transplantation.
This consensus meeting explores whether all known forms of MP, when applied to the pancreas, can increase the recruitment of donor organs for both pancreas and islet transplantation.
Recent experimental animal and human models of ex-vivo pancreas MP appear promising, however application of MP to the pancreas requires standardization that considers the unique characteristics of the pancreas and includes the highest researched evidence to inform MP protocols.

In the current era of MP technology, a consensus report is now necessary regarding the role of pancreas machine perfusion with a view to increasing the donor pool for beta cell replacement in transplantation.

PICO QUESTIONS. REFERENCES. LITERATURE ANALYSIS AND EVIDENCE REPORT. DRAFT STATEMENT AND EXPERT RECOMMENDATIONS.

Population:
Pancreatic transplantation (whole organ transplantation)
Islet transplantation
Intervention: Hypothermic machine perfusion (HMP), normothermic machine perfusion (NMP), persufflation or in-situ normothermic regional perfusion
Comparator: Preservation in static cold storage
Study design: Any study design, clinical or pre-clinical
Exclusion criteria: Any language other than English

1- PANCREATIC TRANSPLANTATION (WHOLE ORGAN TRANSPLANTATION)

EX- SITU HYPOTHERMIC MACHINE PERFUSION (HMP)

PRELIMINARY DRAFT

1. In pancreatic transplantation (whole organ transplantation), should hypothermic machine perfusion be performed at a pressure inferior to 30 mmHg?
2. In pancreatic transplantation (whole organ transplantation), should hypothermic machine perfusion be beneficial if the duration is more than 1 hour and less than 6 hours?
3. In pancreatic transplantation (whole organ transplantation), should hypothermic machine perfusion be performed at a temperature between 4 and 12°C?
4. In pancreatic transplantation (whole organ transplantation), should hypothermic machine perfusion be performed with Belzer-MPS or IGL-1?
5. In pancreatic transplantation (whole organ transplantation), could hypothermic machine perfusion be performed by continuous or pulsatile perfusion?
6. In pancreatic transplantation (whole organ transplantation), could hypothermic machine perfusion be performed either by dual of both the superior mesenteric artery and the splenic artery or by combined single perfusion of both arteries?
7. In pancreatic transplantation (whole organ transplantation), should hypothermic machine perfusion be performed after a complete surgical excision to reduce leakage (from the parenchymal & pancreatic capsule and from arterial vascular reconstruction)?
8. Does hypothermic perfusion for less than 6 hours alter the outcome of pancreatic transplantation?
9. Does the decrease in resistance indexes during hypothermic perfusion correlate with a higher tissue oxygenation of the pancreas transplant?
10. Does the decrease in resistance indexes during hypothermic perfusion correlate with better reperfusion of the pancreas transplant?

PRELIMINARY DRAFT

PICO 1: In pancreatic transplantation (whole organ transplantation), should hypothermic machine perfusion be performed at a pressure inferior to 30 mmHg?

RECOMMENDATION/STATEMENT: In pancreatic transplantation (whole organ transplantation), hypothermic perfusion should be performed at a pressure inferior to 30 mmHg.

Quality of evidence: Low – Expert opinion

Strength of recommendation: Strong for

Analysis of the literature:
No direct comparison between hypothermic perfusion at a pressure below 30 mmHG and above 30 mmHG is available. Furthermore, few indirect comparisons are possible due to the heterogeneity of the criteria of interest (Anatomopathology versus survival after auto-allotransplantation)

Brynger et al. performed a 24-hour perfusion at 40 mmHG on 7 canine pancreases. This perfusion was associated with a transplant weight gain of 135% to 275%. SCS was not associated with transplant weight gain. After allo-transplantation in the canine model, there were 4 deaths on 7 animals in HMP group and 4 deaths on 11 in SCS group.

Since 2010, all hypothermic perfusion protocols in pre-clinical conditions use perfusions lower than or equal to 30 mmHG.

Hamouai et al. performed a 5H perfusion at 30 mmHG (n=4) and 20 mmHG (n=3) in a porcine model. Perfusion at 30mmHG was associated with a weight gain of 46% to 140%. Perfusion at 20mmHG was associated with a weight gain of 15% to 27%.
Branchereau et al. performed a 24H perfusion at 25mmHG (n=9) in a discarded human model. They showed that hypothermic perfusion was associated with moderate oedema at 24H.

Prudhomme et al. performed a 24H perfusion at 15 mmHG (n=1), 20 mmHG (n=1), 25 mmHG (n=1) in a primate model. They showed no differences between the groups based on histology (edema, acinar necrosis, congestion).

References:
Prudhomme T, Renaudin K, Lo Faro ML, Cantarovich D, Kervella D, Minault D, et al. Ex situ hypothermic perfusion of nonhuman primate pancreas: A feasibility study. Artif Organs. 2020 Jul;44(7):736-743. doi: 10.1111/aor.13655.

Branchereau J, Renaudin K, Kervella D, Bernadet S, Karam G, Blancho G, et al. Hypothermic pulsatile perfusion of human pancreas: Preliminary technical feasibility study based on histology. Cryobiology. 2018 Dec;85:56-62. doi: 10.1016/j.cryobiol.2018.10.002.

Hamaoui K, Gowers S, Sandhu B, Vallant N, Cook T, Boutelle M, et al. Development of pancreatic machine perfusion: translational steps from porcine to human models. J Surg Res. 2018 Mar;223:263-274. doi: 10.1016/j.jss.2017.11.052.

Brynger H. Twenty-four-hour preservation of the duct-ligated canine pancreatic allograft. Eur Surg Res. 1975;7(6):341-54. doi: 10.1159/000127819.

PRELIMINARY DRAFT

PICO 2: In pancreatic transplantation (whole organ transplantation), should hypothermic machine perfusion be beneficial if the duration is more than 1 hour and less than 6 hours?

RECOMMENDATION/STATEMENT: In pancreatic transplantation (whole organ transplantation), hypothermic perfusion should be beneficial if the duration is more than 1 hour and less than 6 hours.

Quality of evidence: Low – Expert opinion

Strength of recommendation: Low for

Analysis of the literature:
No direct comparison between a hypothermic perfusion of less than 6 hours and > 6 hours is available in the literature.

Ogbemudia et al. performed a 6H perfusion at 15mmHG on 9 porcine pancreases. They demonstrated that pancreases after HMP had lower reperfusion indices and higher reperfusion rates compared to SCS.

Prudhomme et al. performed a 24H perfusion at 15mmHG on 3 porcine pancreases. They demonstrated a decrease in perfusion indexes during the first 6 hours. Perfusion after 12 hours was associated with the appearance of moderate and then severe interlobular oedema at 24 hours.

Prudhomme et al. performed a 24H perfusion at 15mmHG in a primate model (n=3). They demonstrated the appearance of interacinar edema, congestion and exocrine tissue coagulation necrosis lesions after 12 hours of perfusion.

Branchereau et al. performed a 24H perfusion at 25mmHG (n=9) in a discarded human model. They demonstrated a decrease in perfusion indexes during the first 12 hours.

Karcz et al. performed a 24H perfusion at 20mmHG on 15 porcine pancreases. They found a decrease in perfusion indexes between 3 and 65 minutes

References:
Ogbemudia AE, Hakim G, Dengu F, El-Gilani F, Dumbill R, Mulvey J, et al. Development of ex situ normothermic reperfusion as an innovative method to assess pancreases after preservation. Transpl Int. 2021 Sep;34(9):1630-1642. doi: 10.1111/tri.13990.

Prudhomme T, Kervella D, Ogbemudia AE, Gauttier V, Le Bas-Bernardet S, Minault D, et al. Successful pancreas allotransplantations after hypothermic machine perfusion in a novel diabetic porcine model: a controlled study. Transpl Int. 2021 Feb;34(2):353-364. doi: 10.1111/tri.13797.

Prudhomme T, Renaudin K, Lo Faro ML, Cantarovich D, Kervella D, Minault D, et al. Ex situ hypothermic perfusion of nonhuman primate pancreas: A feasibility study. Artif Organs. 2020 Jul;44(7):736-743. doi: 10.1111/aor.13655.

PRELIMINARY DRAFT

Branchereau J, Renaudin K, Kervella D, Bernadet S, Karam G, Blancho G, et al. Hypothermic pulsatile perfusion of human pancreas: Preliminary technical feasibility study based on histology. Cryobiology. 2018 Dec;85:56-62. doi: 10.1016/j.cryobiol.2018.10.002.

Karcz M, Cook HT, Sibbons P, Gray C, Dorling A, Papalois V. An ex-vivo model for hypothermic pulsatile perfusion of porcine pancreata: hemodynamic and morphologic characteristics. Exp Clin Transplant. 2010 Mar;8(1):55-60.

Brynger H. Twenty-four-hour preservation of the duct-ligated canine pancreatic allograft. Eur Surg Res. 1975;7(6):341-54. doi: 10.1159/000127819.

PRELIMINARY DRAFT

PICO 3: In pancreatic transplantation (whole organ transplantation), should hypothermic machine perfusion be performed at a temperature between 4 and 12°C?

RECOMMENDATION/STATEMENT: In pancreatic transplantation (whole organ transplantation), hypothermic perfusion should be performed at a temperature between 4 and 12ºC.

Quality of evidence: Low – Expert opinion

Strength of recommendation: Strong for

Analysis of the literature:
No study in the literature has evaluated hypothermic perfusion outside the 0-12°C temperature range. All available studies used a temperature range of 4-7°C.

References:
Ogbemudia AE, Hakim G, Dengu F, El-Gilani F, Dumbill R, Mulvey J, et al. Development of ex situ normothermic reperfusion as an innovative method to assess pancreases after preservation. Transpl Int. 2021 Sep;34(9):1630-1642. doi: 10.1111/tri.13990.

Prudhomme T, Kervella D, Ogbemudia AE, Gauttier V, Le Bas-Bernardet S, Minault D, et al. Successful pancreas allotransplantations after hypothermic machine perfusion in a novel diabetic porcine model: a controlled study. Transpl Int. 2021 Feb;34(2):353-364. doi: 10.1111/tri.13797.

Prudhomme T, Renaudin K, Lo Faro ML, Cantarovich D, Kervella D, Minault D, et al. Ex situ hypothermic perfusion of nonhuman primate pancreas: A feasibility study. Artif Organs. 2020 Jul;44(7):736-743. doi: 10.1111/aor.13655.

Branchereau J, Renaudin K, Kervella D, Bernadet S, Karam G, Blancho G, et al. Hypothermic pulsatile perfusion of human pancreas: Preliminary technical feasibility study based on histology. Cryobiology. 2018 Dec;85:56-62. doi: 10.1016/j.cryobiol.2018.10.002.

Hamaoui K, Gowers S, Sandhu B, Vallant N, Cook T, Boutelle M, et al. Development of pancreatic machine perfusion: translational steps from porcine to human models. J Surg Res. 2018 Mar;223:263-274. doi: 10.1016/j.jss.2017.11.052.

Karcz M, Cook HT, Sibbons P, Gray C, Dorling A, Papalois V. An ex-vivo model for hypothermic pulsatile perfusion of porcine pancreata: hemodynamic and morphologic characteristics. Exp Clin Transplant. 2010 Mar;8(1):55-60.

Brynger H. Twenty-four-hour preservation of the duct-ligated canine pancreatic allograft. Eur Surg Res. 1975;7(6):341-54. doi: 10.1159/000127819.

Florack G, Sutherland DE, Heil J, Squifflet JP, Najarian JS. Preservation of canine segmental pancreatic autografts: cold storage versus pulsatile machine perfusion. J Surg Res. 1983 May;34(5):493-504.

Tersigni R, Toledo-Pereyra LH, Pinkham J, Najarian JS. Pancreaticoduodenal preservation by hypothermic pulsatile perfusion for twenty-four hours. Ann Surg. 1975 Dec;182(6):743-8. doi: 10.1097/00000658-197512000-00016.

PRELIMINARY DRAFT

De Gruyl J, Westbroek DL, Macdicken I, Ridderhof E, Verschoor L, van Strik R. Cryoprecipitated plasma perfusion preservation and cold storage preservation of duct-ligated pancreatic allografts. Br J Surg. 1977 Jul;64(7):490-3. doi: 10.1002/bjs.1800640711.

Kenmochi T, Asano T, Nakagouri T, Enomoto K, Isono K, Horie H. Prediction of viability of ischemically damaged canine pancreatic grafts by tissue flow rate with machine perfusion. Transplantation. 1992 Apr;53(4):745-50. doi: 10.1097/00007890-199204000-00007.

PRELIMINARY DRAFT

PICO 4: In pancreatic transplantation (whole organ transplantation), should hypothermic machine perfusion be performed with Belzer-MPS or IGL-1?

RECOMMENDATION/STATEMENT: In pancreatic transplantation (whole organ transplantation), hypothermic perfusion should be performed with Belzer-MPS or IGL-1.

Quality of evidence: Low – Expert opinion

Strength of recommendation: Low for

Analysis of the literature:
Only one recent study has compared the use of 2 preservation solutions during hypothermic perfusion.

Ogbemudia et al. performed a 6H perfusion at 15 mmHG with IGL-2 (n=5) or UW-MPS (n=4). During the 6 hours of cold preservation, amylase and lipase levels were highest in the UWHMP group. After revascularisation using an ex-situ normothermic perfusion model, a rise in perfusate glucose concentration above baseline was observed at 12 minutes after the intraarterial glucose bolus only in the UWHMP group.

All of the other studies carried out after 2010 used IGL-1 or Belzer's solution for perfusion.

References:
Ogbemudia AE, Hakim G, Dengu F, El-Gilani F, Dumbill R, Mulvey J, et al. Development of ex situ normothermic reperfusion as an innovative method to assess pancreases after preservation. Transpl Int. 2021 Sep;34(9):1630-1642. doi: 10.1111/tri.13990.

Prudhomme T, Kervella D, Ogbemudia AE, Gauttier V, Le Bas-Bernardet S, Minault D, et al. Successful pancreas allotransplantations after hypothermic machine perfusion in a novel diabetic porcine model: a controlled study. Transpl Int. 2021 Feb;34(2):353-364. doi: 10.1111/tri.13797.

Prudhomme T, Renaudin K, Lo Faro ML, Cantarovich D, Kervella D, Minault D, et al. Ex situ hypothermic perfusion of nonhuman primate pancreas: A feasibility study. Artif Organs. 2020 Jul;44(7):736-743. doi: 10.1111/aor.13655.

Branchereau J, Renaudin K, Kervella D, Bernadet S, Karam G, Blancho G, et al. Hypothermic pulsatile perfusion of human pancreas: Preliminary technical feasibility study based on histology. Cryobiology. 2018 Dec; 85:56-62. doi: 10.1016/j.cryobiol.2018.10.002.

Hamaoui K, Gowers S, Sandhu B, Vallant N, Cook T, Boutelle M, et al. Development of pancreatic machine perfusion: translational steps from porcine to human models. J Surg Res. 2018 Mar; 223:263-274. doi: 10.1016/j.jss.2017.11.052.

Karcz M, Cook HT, Sibbons P, Gray C, Dorling A, Papalois V. An ex-vivo model for hypothermic pulsatile perfusion of porcine pancreata: hemodynamic and morphologic characteristics. Exp Clin Transplant. 2010 Mar;8(1):55-60.

PRELIMINARY DRAFT

PICO 5: In pancreatic transplantation (whole organ transplantation), could hypothermic machine perfusion be performed by continuous or pulsatile perfusion?

RECOMMENDATION/STATEMENT: In pancreatic transplantation (whole organ transplantation), hypothermic perfusion can be performed by continuous or pulsatile perfusion.

Quality of evidence: Low – Expert opinion

Strength of recommendation: Low for

Analysis of the literature:
There is no data in the literature to support the use of continuous or pulsatile perfusion. All recent data have been acquired with a pulsatile perfusion system (Wavesâ. Waters Medical RM3â).

References:

Ogbemudia AE, Hakim G, Dengu F, El-Gilani F, Dumbill R, Mulvey J, et al. Development of ex situ normothermic reperfusion as an innovative method to assess pancreases after preservation. Transpl Int. 2021 Sep;34(9):1630-1642. doi: 10.1111/tri.13990.

Prudhomme T, Kervella D, Ogbemudia AE, Gauttier V, Le Bas-Bernardet S, Minault D, et al. Successful pancreas allotransplantations after hypothermic machine perfusion in a novel diabetic porcine model: a controlled study. Transpl Int. 2021 Feb;34(2):353-364. doi: 10.1111/tri.13797.

Prudhomme T, Renaudin K, Lo Faro ML, Cantarovich D, Kervella D, Minault D, et al. Ex situ hypothermic perfusion of nonhuman primate pancreas: A feasibility study. Artif Organs. 2020 Jul;44(7):736-743. doi: 10.1111/aor.13655.

Branchereau J, Renaudin K, Kervella D, Bernadet S, Karam G, Blancho G, et al. Hypothermic pulsatile perfusion of human pancreas: Preliminary technical feasibility study based on histology. Cryobiology. 2018 Dec;85:56-62. doi: 10.1016/j.cryobiol.2018.10.002.

Hamaoui K, Gowers S, Sandhu B, Vallant N, Cook T, Boutelle M, et al. Development of pancreatic machine perfusion: translational steps from porcine to human models. J Surg Res. 2018 Mar;223:263-274. doi: 10.1016/j.jss.2017.11.052.

Karcz M, Cook HT, Sibbons P, Gray C, Dorling A, Papalois V. An ex-vivo model for hypothermic pulsatile perfusion of porcine pancreata: hemodynamic and morphologic characteristics. Exp Clin Transplant. 2010 Mar;8(1):55-60.

PRELIMINARY DRAFT

PICO 6: In pancreatic transplantation (whole organ transplantation), could hypothermic machine perfusion be performed either by dual of both the superior mesenteric artery and the splenic artery or by combined single perfusion of both arteries?

RECOMMENDATION/STATEMENT: In pancreatic transplantation (whole organ transplantation), hypothermic perfusion can be performed by continuous or pulsatile perfusion.

Quality of evidence: Low – Expert opinion

Strength of recommendation: Strong for

Analysis of the literature:
No study evaluated isolated perfusion of a pancreatic segment. All studies were performed either by dual of both the superior mesenteric artery and the splenic artery or by combined single perfusion of both arteries.

References:
Ogbemudia AE, Hakim G, Dengu F, El-Gilani F, Dumbill R, Mulvey J, et al. Development of ex situ normothermic reperfusion as an innovative method to assess pancreases after preservation. Transpl Int. 2021 Sep;34(9):1630-1642. doi: 10.1111/tri.13990.

Prudhomme T, Kervella D, Ogbemudia AE, Gauttier V, Le Bas-Bernardet S, Minault D, et al. Successful pancreas allotransplantations after hypothermic machine perfusion in a novel diabetic porcine model: a controlled study. Transpl Int. 2021 Feb;34(2):353-364. doi: 10.1111/tri.13797.

Prudhomme T, Renaudin K, Lo Faro ML, Cantarovich D, Kervella D, Minault D, et al. Ex situ hypothermic perfusion of nonhuman primate pancreas: A feasibility study. Artif Organs. 2020 Jul;44(7):736-743. doi: 10.1111/aor.13655.

Branchereau J, Renaudin K, Kervella D, Bernadet S, Karam G, Blancho G, et al. Hypothermic pulsatile perfusion of human pancreas: Preliminary technical feasibility study based on histology. Cryobiology. 2018 Dec; 85:56-62. doi: 10.1016/j.cryobiol.2018.10.002.

Hamaoui K, Gowers S, Sandhu B, Vallant N, Cook T, Boutelle M, et al. Development of pancreatic machine perfusion: translational steps from porcine to human models. J Surg Res. 2018 Mar; 223:263-274. doi: 10.1016/j.jss.2017.11.052.

Karcz M, Cook HT, Sibbons P, Gray C, Dorling A, Papalois V. An ex-vivo model for hypothermic pulsatile perfusion of porcine pancreata: hemodynamic and morphologic characteristics. Exp Clin Transplant. 2010 Mar;8(1):55-60.

Brynger H. Twenty-four-hour preservation of the duct-ligated canine pancreatic allograft. Eur Surg Res. 1975;7(6):341-54. doi: 10.1159/000127819.

Florack G, Sutherland DE, Heil J, Squifflet JP, Najarian JS. Preservation of canine segmental pancreatic autografts: cold storage versus pulsatile machine perfusion. J Surg Res. 1983 May;34(5):493-504. doi: 10.1016/0022-4804(83)90101-4.

PRELIMINARY DRAFT

Tersigni R, Toledo-Pereyra LH, Pinkham J, Najarian JS. Pancreaticoduodenal preservation by hypothermic pulsatile perfusion for twenty-four hours. Ann Surg. 1975 Dec;182(6):743-8. doi: 10.1097/00000658-197512000-00016.

De Gruyl J, Westbroek DL, Macdicken I, Ridderhof E, Verschoor L, van Strik R. Cryoprecipitated plasma perfusion preservation and cold storage preservation of duct-ligated pancreatic allografts. Br J Surg. 1977 Jul;64(7):490-3. doi: 10.1002/bjs.1800640711.

Kenmochi T, Asano T, Nakagouri T, Enomoto K, Isono K, Horie H. Prediction of viability of ischemically damaged canine pancreatic grafts by tissue flow rate with machine perfusion. Transplantation. 1992 Apr;53(4):745-50. doi: 10.1097/00007890-199204000-00007.

PRELIMINARY DRAFT

PICO 7: In pancreatic transplantation (whole organ transplantation), should hypothermic machine perfusion be performed after a complete surgical excision to reduce leakage (from the parenchymal & pancreatic capsule and from arterial vascular reconstruction)?

RECOMMENDATION/STATEMENT: In pancreatic transplantation (whole organ transplantation), hypothermic perfusion should be performed after a complete surgical excision to reduce leakage.

Quality of evidence: Low – Expert opinion

Strength of recommendation: Strong for

Analysis of the literature:
No studies have compared hypothermic perfusion before or after revision surgery. The results of recent studies (Branchereau et al., Prudhomme et al., Ogbemudi et al.) were obtained with a surgical revision before the perfusion.

References:
0

PRELIMINARY DRAFT

PICO 8: Does hypothermic perfusion for less than 6 hours alter the outcome of pancreatic transplantation?

RECOMMENDATION/STATEMENT: Hypothermic perfusion for less than 6 hours does not alter the outcome of pancreatic transplantation.

Quality of evidence: Low – Expert opinion

Strength of recommendation: Low for

Analysis of the literature:
No direct comparison between a hypothermic perfusion of less than 6 hours and > 6 hours is available in the literature.

Prudhomme et al. performed a 24H perfusion at 15mmHG on 3 porcine pancreases. They demonstrated a decrease in perfusion indexes during the first 6 hours. Perfusion after 12 hours was associated with the appearance of moderate and then severe interlobular oedema at 24 hours.

Prudhomme et al. performed a 24H perfusion at 15mmHG in a primate model (n=3). They demonstrated the appearance of interacinar edema, congestion and exocrine tissue coagulation necrosis lesions after 12 hours of perfusion.

References:
Prudhomme T, Kervella D, Ogbemudia AE, Gauttier V, Le Bas-Bernardet S, Minault D, et al. Successful pancreas allotransplantations after hypothermic machine perfusion in a novel diabetic porcine model: a controlled study. Transpl Int. 2021 Feb;34(2):353-364. doi: 10.1111/tri.13797.

Prudhomme T, Renaudin K, Lo Faro ML, Cantarovich D, Kervella D, Minault D, et al. Ex situ hypothermic perfusion of nonhuman primate pancreas: A feasibility study. Artif Organs. 2020 Jul;44(7):736-743. doi: 10.1111/aor.13655.

PRELIMINARY DRAFT

PICO 9: Does the decrease in resistance indexes during hypothermic perfusion correlate with a higher tissue oxygenation of the pancreas transplant?

RECOMMENDATION/STATEMENT: During hypothermic perfusion, a decrease in resistance indexes is correlated with a higher tissue oxygenation of the pancreas transplant.

Quality of evidence: Low – Expert opinion

Strength of recommendation: Strong for

Analysis of the literature:
No published data have evaluated the relationship and interaction between resistance index and pancreatic parenchymal oxygenation.

References:
0

PRELIMINARY DRAFT

PICO 10: Does the decrease in resistance indexes during hypothermic perfusion correlate with better reperfusion of the pancreas transplant?

RECOMMENDATION/STATEMENT: During hypothermic perfusion, a decrease in resistance indexes is correlated with better reperfusion of the pancreas transplants.

Quality of evidence: Low – Expert opinion

Strength of recommendation: Strong for

Analysis of the literature:
No published data have evaluated the relationship and interaction between resistance index during hypothermic preservation and flow, resistance during reperfusion

References:
0

PRELIMINARY DRAFT

2- ISLET TRANSPLANTATION

EX- SITU HYPOTHERMIC MACHINE PERFUSION (HMP)

1. Should hypothermic perfusion of the pancreas for islet isolation be performed in the same manner as for vascularized pancreas transplantation with regards to: temperature, pressure, perfusate composition, oxygenation, duration, and timing?
2. Could hypothermic perfusion be used to avoid night-time islet isolations?
3. In islet transplantation, could hypothermic perfusion be used to increase cellular energy reserves, especially in donation after circulatory death procedures?

PRELIMINARY DRAFT

PICO 1: Should hypothermic perfusion of the pancreas for islet isolation be performed in the same manner as for vascularized pancreas transplantation with regards to: temperature, pressure, perfusate composition, oxygenation, duration, and timing?

RECOMMENDATION/STATEMENT: Hypothermic perfusion of the pancreas for islet isolation should be performed in the same manner as for vascularized pancreas transplantation with regards to: temperature, pressure, perfusate composition, oxygenation, duration, and timing.

Quality of evidence: Low – Expert opinion

Strength of recommendation: Strong for

Analysis of the literature:
No study has directly compared perfusion protocols for islet isolation. Furthermore, few indirect comparisons are possible due to the heterogeneity of the criteria of interest.

Doppenberg et al. and Leemkuil et al. performed a 6H perfusion at 25 mmHG with UW-MPS in a discarded human model (n=10)

Reddy et al. performed a 6H to 10 mmHG perfusion with UW in a rat model (n=11)

Weegman et al. performed a 24H perfusion at 10 mmHG with KPS-1 in a porcine model (n=4)

Taylor et al. performed a 24H perfusion at 10 mmHG with KPS-1 in a porcine model (n=14).

Leeser et al. performed a 4H perfusion at 10 mmHG with KPS-1 in a discarded human model of end ischemic perfusion (n=4).

Toledo-Pereyra et al. performed a 4H perfusion at 10 mmHG in a discarded human model of end ischemic perfusion (n=4).

The protocols used for islet isolation were identical in the literature to those applied to the pancreas-organ (temperature, perfusion time, perfusion pressure, perfusion solution)

References:
Doppenberg JB, Leemkuil M, Engelse MA, Krikke C, de Koning EJP, Leuvenink HGD. Hypothermic oxygenated machine perfusion of the human pancreas for clinical islet isolation: a prospective feasibility study. Transpl Int. 2021 Aug;34(8):1397-1407. doi: 10.1111/tri.13927.

Leemkuil M, Lier G, Engelse MA, Ploeg RJ, de Koning EJP, 't Hart NA, et al. Hypothermic Oxygenated Machine Perfusion of the Human Donor Pancreas. Transplant Direct. 2018 Sep 7;4(10):e388. doi: 10.1097/TXD.0000000000000829.

Reddy MS, Carter N, Cunningham A, Shaw J, Talbot D. Portal Venous Oxygen Persufflation of the Donation after Cardiac Death pancreas in a rat model is superior to static cold storage and hypothermic machine perfusion. Transpl Int. 2014 Jun;27(6):634-9. doi: 10.1111/tri.12313.

Weegman BP, Taylor MJ, Baicu SC, Scott WE 3rd, Mueller KR, Kitzmann JD, et al. Hypothermic Perfusion Preservation of Pancreas for Islet Grafts: Validation Using a Split Lobe Porcine Model. Cell Med. 2012 Jan 1;2(3):105-110. doi: 10.3727/215517911X617897.

PRELIMINARY DRAFT

Taylor MJ, Baicu S, Greene E, Vazquez A, Brassil J. Islet isolation from juvenile porcine pancreas after 24-h hypothermic machine perfusion preservation. Cell Transplant. 2010;19(5):613-28. doi: 10.3727/096368910X486316.

Leeser DB, Bingaman AW, Poliakova L, Shi Q, Gage F, Bartlett ST, et al. Pulsatile pump perfusion of pancreata before human islet cell isolation. Transplant Proc. 2004 May;36(4):1050-1. doi: 10.1016/j.transproceed.2004.04.041.

Toledo-Pereyra LH, Valgee KD, Castellanos J, Chee M. Hypothermic pulsatile perfusion: its use in the preservation of pancreases for 24 to 48 hours before islet cell transplantation. Arch Surg. 1980 Jan;115(1):95-8. doi: 10.1001/archsurg.1980.01380010081022.

PRELIMINARY DRAFT

PICO 2: Could hypothermic perfusion be used to avoid night-time islet isolations?

RECOMMENDATION/STATEMENT: Hypothermic perfusion could be used to avoid night-time islet isolations.

Quality of evidence: Low – Expert opinion

Strength of recommendation: Low for

Analysis of the literature:
Taylor et al. performed a 24H perfusion at 10 mmHG with KPS-1 in a porcine model (n=14). They compared outcomes with isolation from fresh pancreas (CIT < 2H) and from pancreas preserved by 24H SCS. Islet isolation after 24H perfusion resulted in significantly higher islet mass than after 24H SCS. Islet mass was however significantly lower than after fresh extraction. Mean insulin content of islets isolated from perfused pancreata was significantly higher than that of 24H SCS but not different to the fresh tissue.

Leeser et al. performed a 4H perfusion at 10 mmHG with KPS-1 in a discarded human model of end ischemic perfusion (n=4) (Mean previous CIT: 13 Hours). They compared outcomes with isolation from pancreas by SCS <8H and from pancreas by SCS >8H. The islet yield for the four pumped pancreata was 3435 (+/-1951) islet equivalents/gram pancreas tissue (IEQ/g), compared with a mean yield of 5134 (+/-2700) IEQ/g and 2640 (+/-1000) IEQ/g from pancreas with <8 hours and >8 hours CIT, respectively. The mean viability after machine pulsatile perfusion was 86% (vs 74% and 74% for the <8 hour and >8 hour CIT groups). The mean viable yield (total yield x viability) was 2937 IEQ/g for machine perfusion, compared with 3799 IEQ/g and 1937 IEQ/g from pancreata with <8 hours and >8 hours CIT, respectively. The insulin secretion index of islets after machine perfusion was 6.4, compared with indices of 1.9 and 1.8 for the <8 hour and >8 hour CIT groups.

References:
Taylor MJ, Baicu S, Greene E, Vazquez A, Brassil J. Islet isolation from juvenile porcine pancreas after 24-h hypothermic machine perfusion preservation. Cell Transplant. 2010;19(5):613-28. doi: 10.3727/096368910X486316.

Leeser DB, Bingaman AW, Poliakova L, Shi Q, Gage F, Bartlett ST, et al. Pulsatile pump perfusion of pancreata before human islet cell isolation. Transplant Proc. 2004 May;36(4):1050-1. doi: 10.1016/j.transproceed.2004.04.041.

PRELIMINARY DRAFT

PICO 3: In islet transplantation, could hypothermic perfusion be used to increase cellular energy reserves, especially in donation after circulatory death procedures?

RECOMMENDATION/STATEMENT: In islet transplantation, hypothermic perfusion could be used to increase cellular energy reserves, especially in donation after circulatory death procedures.

Quality of evidence: Moderate

Strength of recommendation: Strong for

Analysis of the literature:
Leemkuil et al. performed a 6H perfusion at 25 mmHG with UW-MPS in a discarded human model (n=10, 5 from DBD, 5 from DCD). The comparative group consisted of pancreas preserved in SCS (n=10, 5 from DBD, 5 from DCD). After prolonged SCS, ATP concentration decreased further to 2.9 (IQR, 2.8-5.7) μmol/g protein in DCD pancreata and 22.8 (IQR, 20.6-46) μmol/g protein in DBD pancreata ($P < 0.05$). After HMP ATP concentration in both DCD and DBD pancreata increased significantly ($P < 0.05$). In the DCD group a 6.8-fold increase and in the DBD group a 2.6-fold increase was observed. Interestingly, ATP concentration in DCD pancreata reached the same level of DBD pancreata: 100.5 (IQR, 49.4-169.8) and 109.3 (IQR, 65.2-301.5) μmol/gprotein in DCD and DBD pancreata, respectively.

Taylor et al. performed a 24H perfusion at 10 mmHG with KPS-1 in a porcine model (n=14). They compared outcomes with isolation from fresh pancreas (CIT < 2H) and from pancreas preserved by 24H SCS. They found that the energy status of the isolated islets, in terms of ATP content, was preserved during the 24-h perfusion

References:
Leemkuil M, Lier G, Engelse MA, Ploeg RJ, de Koning EJP, 't Hart NA, et al. Hypothermic Oxygenated Machine Perfusion of the Human Donor Pancreas. Transplant Direct. 2018 Sep 7;4(10):e388. doi: 10.1097/TXD.0000000000000829.

Taylor MJ, Baicu S, Greene E, Vazquez A, Brassil J. Islet isolation from juvenile porcine pancreas after 24-h hypothermic machine perfusion preservation. Cell Transplant. 2010;19(5):613-28. doi: 10.3727/096368910X486316.

PRELIMINARY DRAFT

2- ISLET TRANSPLANTATION
PERSUFFLATION

PICO QUESTIONS

1. In islet transplantation, should persufflation be performed using a humidified gaseous flow of 40% oxygen and 60% nitrogen?
2. In islet transplantation, should persufflation be performed at a temperature of 4-6°C in an organ preservation solution?
3. In islet transplantation, should persufflation be performed using a gaseous flow rate of 25 ml/hr?
4. In islet transplantation, could persufflation be performed by canulation of the superior mesenteric artery and the splenic artery and optionally the pancreaticoduodenal artery?
5. In islet transplantation, should arterial leakages be closed until the gaseous outflow is mainly venous when starting persufflation?
6. In islet transplantation, could persufflation be prevent further cold ischemic damage for up to 24 hours?
7. In islet transplantation, could persufflation be performed during organ transport as an end-ischemic model?
8. In islet transplantation, could persufflation attenuate pro-inflammatory signalling in isolated islets?

PRELIMINARY DRAFT

PICO 1: In islet transplantation, should persufflation be performed using a humidified gaseous flow of 40% oxygen and 60% nitrogen?

RECOMMENDATION/STATEMENT: In islet transplantation, persufflation should be performed using a humidified gaseous flow of 40% oxygen and 60% nitrogen.

Quality of evidence: Low – Expert opinion

Strength of recommendation: Strong for

Analysis of the literature:
No study has directly compared persufflation protocols for islet isolation. Furthermore, few indirect comparisons are possible due to the heterogeneity of the criteria of interest.

Reddy et al. performed venous persufflation with pure oxygen at a pressure of 10 mmHG in a rat model (n=11).

Kelly et al. performed arterial persufflation with 40% humidified oxygen in a discarded human model (n=13).

Scott et al. performed arterial persufflation with 40% humidified oxygen in a porcine model.

References:
Reddy MS, Carter N, Cunningham A, Shaw J, Talbot D. Portal Venous Oxygen Persufflation of the Donation after Cardiac Death pancreas in a rat model is superior to static cold storage and hypothermic machine perfusion. Transpl Int. 2014 Jun;27(6):634-9. doi: 10.1111/tri.12313.

Kelly AC, Smith KE, Purvis WG, Min CG, Weber CS, Cooksey AM, et al. Oxygen Perfusion (Persufflation) of Human Pancreata Enhances Insulin Secretion and Attenuates Islet Proinflammatory Signaling. Transplantation. 2019 Jan;103(1):160-167. doi: 10.1097/TP.0000000000002400.

Scott WE 3rd, O'Brien TD, Ferrer-Fabrega J, Avgoustiniatos ES, Weegman BP, Anazawa T, et al. Persufflation improves pancreas preservation when compared with the two-layer method. Transplant Proc. 2010 Jul-Aug;42(6):2016-9. doi: 10.1016/j.transproceed.2010.05.092.

PRELIMINARY DRAFT

PICO 2: In islet transplantation, should persufflation be performed at a temperature of 4-6°C in an organ preservation solution?

RECOMMENDATION/STATEMENT: In islet transplantation, persufflation should be performed at a temperature of 4-6°C in an organ preservation solution.

Quality of evidence: Low – Expert opinion

Strength of recommendation: Strong for

Analysis of the literature:
No study in the literature has evaluated persufflation outside the 0-12°C temperature range. All available studies used a temperature range of 4-6°C.

References:
Reddy MS, Carter N, Cunningham A, Shaw J, Talbot D. Portal Venous Oxygen Persufflation of the Donation after Cardiac Death pancreas in a rat model is superior to static cold storage and hypothermic machine perfusion. Transpl Int. 2014 Jun;27(6):634-9. doi: 10.1111/tri.12313.

Kelly AC, Smith KE, Purvis WG, Min CG, Weber CS, Cooksey AM, et al. Oxygen Perfusion (Persufflation) of Human Pancreata Enhances Insulin Secretion and Attenuates Islet Proinflammatory Signaling. Transplantation. 2019 Jan;103(1):160-167. doi: 10.1097/TP.0000000000002400.

Scott WE 3rd, O'Brien TD, Ferrer-Fabrega J, Avgoustiniatos ES, Weegman BP, Anazawa T, et al. Persufflation improves pancreas preservation when compared with the two-layer method. Transplant Proc. 2010 Jul-Aug;42(6):2016-9. doi: 10.1016/j.transproceed.2010.05.092.

PRELIMINARY DRAFT

PICO 3: In islet transplantation, should persufflation be performed using a gaseous flow rate of 25 ml/hr?

RECOMMENDATION/STATEMENT: In islet transplantation, persufflation should be performed using a gaseous flow rate of 25 ml/hr.

Quality of evidence: Low – Expert opinion

Strength of recommendation: Low for

Analysis of the literature:
No studies have evaluated the optimal oxygen flow rate during perfusion.

Reddy et al. performed venous persufflation with pure oxygen at a pressure of 10 mmHG in a rat model (n=11).

Scott et al. performed arterial persufflation with 40% humidified oxygen in a porcine model with oxygen flow at 20mL/min.

References:
Reddy MS, Carter N, Cunningham A, Shaw J, Talbot D. Portal Venous Oxygen Persufflation of the Donation after Cardiac Death pancreas in a rat model is superior to static cold storage and hypothermic machine perfusion. Transpl Int. 2014 Jun;27(6):634-9. doi: 10.1111/tri.12313.

Scott WE 3rd, O'Brien TD, Ferrer-Fabrega J, Avgoustiniatos ES, Weegman BP, Anazawa T, et al. Persufflation improves pancreas preservation when compared with the two-layer method. Transplant Proc. 2010 Jul-Aug;42(6):2016-9. doi: 10.1016/j.transproceed.2010.05.092.

PRELIMINARY DRAFT

PICO 4: In islet transplantation, could persufflation be performed by canulation of the superior mesenteric artery and the splenic artery and optionally the pancreaticoduodenal artery?

RECOMMENDATION/STATEMENT: In islet transplantation, persufflation can be performed by canulation of the superior mesenteric artery and the splenic artery and optionally the pancreaticoduodenal artery.

Quality of evidence: Low – Expert opinion

Strength of recommendation: Strong for

Analysis of the literature:
No study evaluated isolated perfusion of a pancreatic segment.

Kelly et al. and Scoot and et. performed the persufflation by canulation of the superior mesenteric artery and the splenic artery.

Reddy et al. performed the persufflation by canulation of the portal vein

References:
Reddy MS, Carter N, Cunningham A, Shaw J, Talbot D. Portal Venous Oxygen Persufflation of the Donation after Cardiac Death pancreas in a rat model is superior to static cold storage and hypothermic machine perfusion. Transpl Int. 2014 Jun;27(6):634-9. doi: 10.1111/tri.12313.

Kelly AC, Smith KE, Purvis WG, Min CG, Weber CS, Cooksey AM, et al. Oxygen Perfusion (Persufflation) of Human Pancreata Enhances Insulin Secretion and Attenuates Islet Proinflammatory Signaling. Transplantation. 2019 Jan;103(1):160-167. doi: 10.1097/TP.0000000000002400.

Scott WE 3rd, O'Brien TD, Ferrer-Fabrega J, Avgoustiniatos ES, Weegman BP, Anazawa T, et al. Persufflation improves pancreas preservation when compared with the two-layer method. Transplant Proc. 2010 Jul-Aug;42(6):2016-9. doi: 10.1016/j.transproceed.2010.05.092.

PRELIMINARY DRAFT

PICO 5: In islet transplantation, should arterial leakages be closed until the gaseous outflow is mainly venous when starting persufflation?

RECOMMENDATION/STATEMENT: When starting persufflation, arterial leakages must be closed, until the gaseous outflow is mainly venous.

Quality of evidence: Low – Expert opinion

Strength of recommendation: Strong for

Analysis of the literature:
No study has evaluated the results of persufflation according to whether or not a revision surgery was performed

In Kelly et al. study, leaks were identified and ligated with surgical silk and portal-venous outflow was observed to ensure that oxygen was dispersed throughout the organ.

References:
Kelly AC, Smith KE, Purvis WG, Min CG, Weber CS, Cooksey AM, et al. Oxygen Perfusion (Persufflation) of Human Pancreata Enhances Insulin Secretion and Attenuates Islet Proinflammatory Signaling. Transplantation. 2019 Jan;103(1):160-167. doi: 10.1097/TP.0000000000002400.

PRELIMINARY DRAFT

PICO 6: In islet transplantation, could persufflation be prevent further cold ischemic damage for up to 24 hours?

RECOMMENDATION/STATEMENT: In islet transplantation, persufflation can be prevent further cold ischemic damage for up to 24 hours.

Quality of evidence: Low – Expert opinion

Strength of recommendation: Low for

Analysis of the literature:
Scott et al. performed arterial persufflation with 40% humidified oxygen in a porcine model for 24 hours. They compared the results with those obtained in TLM on a segment of the same pancreas. Histological analyses were performed. Biopsies taken from organs preserved for 24 hours with PSF exhibited distended capillaries and less autolysis/necrosis as compared to tissues preserved by TLM for 24 hours. Pyknotic nuclei in TLM samples, which suggest that irreversible damage has occurred, were observed. Occasionally, clots were observed in PSF organs, preventing gas flow to these tissues. Histologically, these regions exhibited increased autolysis correlating with the presence of red blood cells and pyknotic nuclei. When biopsies were compared from two of these organs, there was significantly greater autolysis present in nonpersufflated regions.

References:
Scott WE 3rd, O'Brien TD, Ferrer-Fabrega J, Avgoustiniatos ES, Weegman BP, Anazawa T, et al. Persufflation improves pancreas preservation when compared with the two-layer method. Transplant Proc. 2010 Jul-Aug;42(6):2016-9. doi: 10.1016/j.transproceed.2010.05.092.

PRELIMINARY DRAFT

PICO 7: In islet transplantation, could persufflation be performed during organ transport as an end-ischemic model?

RECOMMENDATION/STATEMENT: In islet transplantation, persufflation can be performed during organ transport as an end-ischemic model.

Quality of evidence: Low – Expert opinion

Strength of recommendation: Low for

Analysis of the literature:
Kelly et al. performed arterial persufflation with 40% humidified oxygen in a discarded human model (n=13). Persufflation was performed after a period of SCS. The persufflation group was compared to preservation by SCS alone with equivalent total cold ischemia times.

Islet purity and viability were no different between SCS and SCS + PSF groups. When total ischemia time was matched between SCS + PSF and SCS groups, PSF improved insulin secretion ($P < 0.05$, Figure 3B), demonstrated by stimulation indices of 7.3 ± 2.1 and 2.5 ± 0.4, respectively.

References:
Kelly AC, Smith KE, Purvis WG, Min CG, Weber CS, Cooksey AM, et al. Oxygen Perfusion (Persufflation) of Human Pancreata Enhances Insulin Secretion and Attenuates Islet Proinflammatory Signaling. Transplantation. 2019 Jan;103(1):160-167. doi: 10.1097/TP.0000000000002400.

PRELIMINARY DRAFT

PICO 8: In islet transplantation, could persufflation attenuate pro-inflammatory signalling in isolated islets?

RECOMMENDATION/STATEMENT: In islet transplantation, persufflation may attenuate pro-inflammatory signaling in isolated islets.

Quality of evidence: Moderate

Strength of recommendation: Low for

Analysis of the literature:
Kelly et al. performed arterial persufflation with 40% humidified oxygen in a discarded human model (n=13). Persufflation was performed after a period of SCS. The persufflation group was compared to preservation by SCS alone with equivalent total cold ischaemia times. They evaluated the differential gene expression between the 2 groups after islet isolation by RNAseq (Reverse transcriptase and qPCR).

In the 2 groups, the most abundant genes were insulin (INS), insulin like growth factor 2 (IGF2), and the INS-IGF2 read-through transcript variant. Differential expression was not analyzed for these genes or 4 abundant protein-coding genes: glucagon, pancreatic enzyme elongation factor 1 complex, transthyretin, and pancreatic carboxypeptidase A1.

One hundred transcripts (0.5% of total) were differentially expressed in SCS + PSF islets versus SCS islets.
The most downregulated genes in SCS + PSF compared to SCS islets included: chemokine ligand 20 (CCL20), interleukin 1, beta (IL1B), and chemokine ligand 5 (CXCL5). Genes downstream of IL1β were also lower; matrix metallopeptidase 1 (MMP1) and caspase recruitment domain family member 11 (CARD11). Functional analysis of genes lower in SCS + PSF islets identified inflammation.
The most upregulated genes in SCS + PSF islets are enriched for cellular metabolism, pancreatic secretion, and ion transport.

References:
Kelly AC, Smith KE, Purvis WG, Min CG, Weber CS, Cooksey AM, et al. Oxygen Perfusion (Persufflation) of Human Pancreata Enhances Insulin Secretion and Attenuates Islet Proinflammatory Signaling. Transplantation. 2019 Jan;103(1):160-167. doi: 10.1097/TP.0000000000002400.

PRELIMINARY DRAFT

1- PANCREATIC TRANSPLANTATION (WHOLE ORGAN TRANSPLANTATION)
EX-SITU NORMOTHERMIC MACHINE PERFUSION (NMP)

PICO QUESTIONS

1. Could ex situ normothermic machine perfusion be a reliable method for evaluating (functional assessment) explanted pancreas after static cold preservation in pancreatic transplantation (whole organ transplantation)?
2. In pancreatic transplantation (whole organ transplantation), should ex situ normothermic machine perfusion be performed at physiologic temperature, with perfusate solution containing an oxygen carrier to sustain metabolic activities of the cells?
3. In pancreatic transplantation (whole organ transplantation), should ex situ normothermic machine perfusion be performed at a pressure range from 25 to 50 mmHg?
4. In pancreatic transplantation (whole organ transplantation), does ex situ normothermic machine perfusion require a balance of pressure and flow to ensure minimal damage to the endothelium?
5. In ex situ normothermic machine perfusion for pancreatic transplantation, does the addition of an oncotic factor to the perfusate regulate the oncotic pressure and minimize edema formation (thus allowing higher pressure perfusions)?
6. In ex situ normothermic machine perfusion for pancreatic transplantation, does the addition of enzymatic inhibitor in the perfusate (i.e. aprotinin) reduce graft necrosis and allow for longer perfusions?
7. In pancreatic transplantation (whole organ transplantation), should ex situ normothermic machine perfusion be beneficial if the duration is more than 1 hour and less than 6 hours?
8. In pancreatic transplantation (whole organ transplantation), could ex situ normothermic machine perfusion be performed by continuous or pulsatile perfusion?
9. In case of prolonged perfusion, does ex situ normothermic machine perfusion require the management of exocrine secretions (dialysis membrane, duodenal diversion) to prevent the development of graft edema?
10. Are pancreatic grafts metabolically active during perfusion as evidenced by measuring oxygen extraction?
11. During ex situ normothermic machine perfusion for pancreatic transplantation, could the endocrine function of the pancreas graft be assessed by insulin secretion tests (under glucose or arginine stimulation)?
12. During ex situ normothermic machine perfusion for pancreatic transplantation, could preservation of pancreatic exocrine function be assessed by amylase and lipase levels in the perfusate/pancreatic juice?
13. Should ex situ normothermic machine perfusion for pancreatic transplantation be performed simultaneously through the superior mesenteric artery and the splenic artery?

PRELIMINARY DRAFT

PICO 1: Could ex situ normothermic machine perfusion be a reliable method for evaluating (functional assessment) explanted pancreas after static cold preservation in pancreatic transplantation (whole organ transplantation)?

RECOMMENDATION/STATEMENT: Ex situ normothermic machine perfusion can be a reliable method for evaluating (functional assessment) explanted pancreas after static cold preservation in pancreatic transplantation (whole organ transplantation).

Quality of evidence: Low – Expert opinion

Strength of recommendation: Strong for

Analysis of the literature:
Preservation of organs under almost physiologic conditions (NMP) prior to transplantation offers an important advantage for functional assessment of the organ.
The three referenced studies (i.e Mazilescu LI, Barlow AD, Nassar A) have demonstrated the safety of pancreas transplantation after NMP in experimental porcine models and human discarded pancreas.
However, a number of studies have been reported, either experimental or pre-clinical, and the vast majority used a deficient number of pancreas and are mainly based on histological findings and poor biochemical analysis. Different NMP systems were subsequently developed for different animal models to assess viability and function after various durations and storage conditions
At present, the ex situ NMP technique allows organ monitoring and estimation of its quality before pancreas transplantation.

References:
M R Eloy, J Kachelhoffer, A Pousse, J Dauchel, J F Grenier. Ex vivo vascular perfusion of the isolated canine pancreas. Experimental procedure, haemodynamic data and experimental applications. *Eur Surg Res*. **(1974)** 6(6):341-53. doi: 10.1159/000127741.

Eckhauser F, Knol JA, Porter-Fink V, Lockery D, Edgcomb L, Strodel WE, Webb D, Simmons J. Ex vivo normothermic hemoperfusion of the canine pancreas: applications and limitations of a modified experimental preparation. *J Surg Res*. **(1981)** 31(1):22-37. doi: 10.1016/0022-4804(81)90026-3.

Pegg DE, Klempnauer J, Diaper MP, Taylor MJ. Assessment of hypothermic preservation of the pancreas in the rat by a normothermic perfusion assay. *J Surg Res*. **(1982)** 33(3):194-200.

Wahlberg J, Southard JH, Belzer FO. Preservation-induced pancreatitis in an isolated perfused pancreas model in the dog. *Transpl Int.* **(1989)** 2(3):165-7. doi: 10.1007/BF02414603.

Kuan KG, Wee MN, Chung WY, Kumar R, Mees ST, Dennison A, et al. A Study of Normothermic Hemoperfusion of the Porcine Pancreas and Kidney. *Artif Organs*. **(2017)** 41(5):490-495. doi: 10.1111/aor.12770.

Kumar R, Chung WY, Runau F, Isherwood JD, Kuan KG, West K, et al. Ex vivo normothermic porcine pancreas: A physiological model for preservation and transplant study. *Int J Surg*. **(2018)** 54(Pt A):206-215. doi: 10.1016/j.ijsu.2018.04.057.

Mazilescu LI, Parmentier C, Kalimuthu SN, Ganesh S, Kawamura M, Goto T, et al. Normothermic ex situ pancreas perfusion for the preservation of porcine pancreas grafts. *Am J Transplant.* **(2022)** 22(5):1339-1349. doi: 10.1111/ajt.17019.

PRELIMINARY DRAFT

Barlow AD, Hamed MO, Mallon DH, Brais RJ, Gribble FM, Scott MA, et al. Use of Ex Vivo Normothermic Perfusion for Quality Assessment of Discarded Human Donor Pancreases. *Am J Transplant.* **(2015)** 15(9):2475-82. doi: 10.1111/ajt.13303.

Nassar A, Liu Q, Walsh M, Quintini C. Normothermic Ex Vivo Perfusion of Discarded Human Pancreas. *Artif Organs*. **(2018)** 42(3):334-335. doi: 10.1111/aor.12985.

PRELIMINARY DRAFT

PICO 2: In pancreatic transplantation (whole organ transplantation), should ex situ normothermic machine perfusion be performed at physiologic temperature, with perfusate solution containing an oxygen carrier to sustain metabolic activities of the cells?

RECOMMENDATION/STATEMENT: In pancreatic transplantation (whole organ transplantation), ex situ normothermic machine perfusion should be performed at physiologic temperature (preferable 37ºC, range 34-38ºC), with perfusate solution containing an oxygen carrier to sustain metabolic activities of the cells

Quality of evidence: Low – Expert opinion

Strength of recommendation: Strong for

Analysis of the literature:
No study in the literature has evaluated normothermic perfusion outside the 34-38°C temperature range. All available studies used a temperature range of 37°C, except for Mazilescu LI et al (36ºC) and Pegg DE et al (38ºC).

References:
M R Eloy, J Kachelhoffer, A Pousse, J Dauchel, J F Grenier. Ex vivo vascular perfusion of the isolated canine pancreas. Experimental procedure, haemodynamic data and experimental applications. *Eur Surg Res*. **(1974)** 6(6):341-53. doi: 10.1159/000127741.

Eckhauser F, Knol JA, Porter-Fink V, Lockery D, Edgcomb L, Strodel WE, Webb D, Simmons J. Ex vivo normothermic hemoperfusion of the canine pancreas: applications and limitations of a modified experimental preparation. *J Surg Res*. **(1981)** 31(1):22-37. doi: 10.1016/0022-4804(81)90026-3.

Pegg DE, Klempnauer J, Diaper MP, Taylor MJ. Assessment of hypothermic preservation of the pancreas in the rat by a normothermic perfusion assay. *J Surg Res*. **(1982)** 33(3):194-200. doi: 10.1016/0022-4804(82)90029-4.

Wahlberg J, Southard JH, Belzer FO. Preservation-induced pancreatitis in an isolated perfused pancreas model in the dog. *Transpl Int.* **(1989)** 2(3):165-7. doi: 10.1007/BF02414603.

Kuan KG, Wee MN, Chung WY, Kumar R, Mees ST, Dennison A, et al. A Study of Normothermic Hemoperfusion of the Porcine Pancreas and Kidney. *Artif Organs*. **(2017)** 41(5):490-495. doi: 10.1111/aor.12770.

Kumar R, Chung WY, Runau F, Isherwood JD, Kuan KG, West K, et al. Ex vivo normothermic porcine pancreas: A physiological model for preservation and transplant study. *Int J Surg*. **(2018)** 54(Pt A):206-215. doi: 10.1016/j.ijsu.2018.04.057.

Mazilescu LI, Parmentier C, Kalimuthu SN, Ganesh S, Kawamura M, Goto T, et al. Normothermic ex situ pancreas perfusion for the preservation of porcine pancreas grafts. *Am J Transplant*. **(2022)** 22(5):1339-1349. doi: 10.1111/ajt.17019.

Barlow AD, Hamed MO, Mallon DH, Brais RJ, Gribble FM, Scott MA, et al. Use of Ex Vivo Normothermic Perfusion for Quality Assessment of Discarded Human Donor Pancreases. *Am J Transplant.* **(2015)** 15(9):2475-82. doi: 10.1111/ajt.13303.

Nassar A, Liu Q, Walsh M, Quintini C. Normothermic Ex Vivo Perfusion of Discarded Human Pancreas. *Artif Organs*. **(2018)** 42(3):334-335. doi: 10.1111/aor.12985.

PICO 3: In pancreatic transplantation (whole organ transplantation), should ex situ normothermic machine perfusion be performed at a pressure range from 25 to 50 mmHg?

PRELIMINARY DRAFT

RECOMMENDATION/STATEMENT: In pancreatic transplantation (whole organ transplantation), ex situ normothermic machine perfusion should be performed at a pressure range from 25 to 50 mmHg

Quality of evidence: Low – Expert opinion

Strength of recommendation: Strong for

Analysis of the literature:
Animal model
The perfusion pressure in the study of Kuan KG et al was 70–80 mmHg for pancreas alone, 90 -100 mmHg for pancreas + kidney, which could be considered too high for the pancreatic organ and thus a contributor to the acinar injury.

Kumar R et al: 50 mmHg (control group; high), 20 mmHg (low pressure group). From other group studies (data not shown at 80, 70 and 60 mmHg) they stablished that 50 mmHg of pressure was optimal to prevent edema in the graft. The same group directly studied the effects of ex situ NMP testing two different pressure perfusions in a porcine DCD pancreata model (Kumar R et al). In this study, an oxygenated non-pulsatile pump was used with a whole blood-based perfusate to which heparin was added to reduce coagulation. The control group was perfused at 50 mmHg and was compared to an experimental group of 'low pressure' grafts perfused at 20 mmHg. Both pancreatic blood flow and pressure remained stable, but control grafts achieved a mean blood flow of 140 ml/min, while the low-pressure group achieved a blood flow of only 40 ml/min, indicating a worse perfusion. All grafts showed evidence of oxygen absorption and cellular viability, as corroborated with immunohistochemistry. Both endocrine and exocrine functionality were preserved, but amylase levels were significantly lower at all time points in the low-pressure group compared with controls. Notwithstanding, *cell viability and ATP synthetase stain showed an improved score grade in the low-pressure group*. This is the only study on ex situ NMP in porcine pancreas that measures ATPase activity, a marker of viability which has been related to better transplantation outcome (Bellini MI). Despite some controversy in the results, the authors suggest *low pressure perfusion* as an improved method for graft preservation during ex situ NMP.

Mazilescu LI et al: Normothermic ex situ pancreas perfusion was initiated with an arterial pressure set to 25 mm Hg and was maintained at 25 mm Hg throughout the whole perfusion. Venous pressure was maintained at around −1 mm Hg by regulation of the height of the venous reservoir. Arterial blood flow rate was initially around 120 ± 21 ml/min. During the perfusion there was a slight decrease in the flow, and towards the end of perfusion arterial flow was around 101 ± 15 ml/ min

Eckhauser F et al: 90-110 initially; then 30-50 mmHg. No fixed perfusion pressure.

Wahlberg J et al: 30 mmHg

Pegg DE et al: 60 mmHg

Human model

PRELIMINARY DRAFT

Barlow AD et al: Arterial pressure was maintained at 50-55 mmHg, which could be considered high. Furthermore, the incorporation of a dialysis unit to the system or the addition of antiproteases to the perfusate are two potential factors that could help reduce pancreas injury.

Nassar A et al: 60 mmHg

References:

Eckhauser F, Knol JA, Porter-Fink V, Lockery D, Edgcomb L, Strodel WE, Webb D, Simmons J. Ex vivo normothermic hemoperfusion of the canine pancreas: applications and limitations of a modified experimental preparation. *J Surg Res*. **(1981)** 31(1):22-37. doi: 10.1016/0022-4804(81)90026-3.

Pegg DE, Klempnauer J, Diaper MP, Taylor MJ. Assessment of hypothermic preservation of the pancreas in the rat by a normothermic perfusion assay. *J Surg Res*. **(1982)** 33(3):194-200. doi: 10.1016/0022-4804(82)90029-4.

Wahlberg J, Southard JH, Belzer FO. Preservation-induced pancreatitis in an isolated perfused pancreas model in the dog. *Transpl Int.* **(1989)** 2(3):165-7. doi: 10.1007/BF02414603.

Kuan KG, Wee MN, Chung WY, Kumar R, Mees ST, Dennison A, et al. A Study of Normothermic Hemoperfusion of the Porcine Pancreas and Kidney. *Artif Organs*. **(2017)** 41(5):490-495. doi: 10.1111/aor.12770.

Kumar R, Chung WY, Runau F, Isherwood JD, Kuan KG, West K, et al. Ex vivo normothermic porcine pancreas: A physiological model for preservation and transplant study. *Int J Surg*. **(2018)** 54(Pt A):206-215. doi: 10.1016/j.ijsu.2018.04.057.

Mazilescu LI, Parmentier C, Kalimuthu SN, Ganesh S, Kawamura M, Goto T, et al. Normothermic ex situ pancreas perfusion for the preservation of porcine pancreas grafts. *Am J Transplant.* **(2022)** 22(5):1339-1349. doi: 10.1111/ajt.17019.

Barlow AD, Hamed MO, Mallon DH, Brais RJ, Gribble FM, Scott MA, et al. Use of Ex Vivo Normothermic Perfusion for Quality Assessment of Discarded Human Donor Pancreases. *Am J Transplant.* **(2015)** 15(9):2475-82. doi: 10.1111/ajt.13303.

Nassar A, Liu Q, Walsh M, Quintini C. Normothermic Ex Vivo Perfusion of Discarded Human Pancreas. *Artif Organs*. **(2018)** 42(3):334-335. doi: 10.1111/aor.12985.

PICO 4: In pancreatic transplantation (whole organ transplantation), does ex situ normothermic machine perfusion require a balance of pressure and flow to ensure minimal damage to the endothelium?

RECOMMENDATION/STATEMENT: In pancreatic transplantation (whole organ transplantation), ex situ normothermic machine perfusion requires a balance of pressure and flow to ensure minimal damage to the endothelium

Quality of evidence: Low – Expert opinion

Strength of recommendation: Strong for

PRELIMINARY DRAFT

Analysis of the literature:
Kumar and colleagues described a model of ex situ normothermic porcine pancreas perfusion. Porcine pancreata were perfused at high (50 mmHg; control group) and low (20 mmHg; "low pressure" group) pressure and graft viability was compared between the two groups. Grafts in the control group achieved a mean blood flow of 140ml/min, while the ones in the low pressure group had a blood flow of only 40 ml/min. Grafts from both groups showed comparable oxygen consumption rates and pancreatic juice consumption rates. Amylase levels were lower in the low pressure group, and immunohistochemistry showed less cellular death in the low pressure group.

In Mazilescu study, despite using a low pressure of 25 mmHg, they achieved higher blood flow rates (90–160 ml/min) while still maintaining pancreas tissue integrity. Kumar et al also failed to demonstrate functionality of the grafts after perfusion (at 20 mmHg and low flow).
In the study of Mazilescu, despite using a lower pressure of 25 mmHg, they achieved a higher flow, which allowed for a better perfusion of the graft.

Despite the difficulty of making firm conclusions from the research findings on pancreas ex situ NMP, which are based strictly on published data, it seems at this point feasible to recommend the use of lower arterial pressure to avoid endothelial injury while achieving higher flow rates to allow a better perfusion. A balance of pressure and flow in NMP is crucial to ensure minimal damage to the vascular endothelium avoiding platelet activation and vascular thrombosis.

References:
Kumar R, Chung WY, Runau F, Isherwood JD, Kuan KG, West K, et al. Ex vivo normothermic porcine pancreas: A physiological model for preservation and transplant study. *Int J Surg*. **(2018)** 54(Pt A):206-215. doi: 10.1016/j.ijsu.2018.04.057.

Mazilescu LI, Parmentier C, Kalimuthu SN, Ganesh S, Kawamura M, Goto T, et al. Normothermic ex situ pancreas perfusion for the preservation of porcine pancreas grafts. *Am J Transplant.* **(2022)** 22(5):1339-1349. doi: 10.1111/ajt.17019.

PICO 5: In ex situ normothermic machine perfusion for pancreatic transplantation, does the addition of an oncotic factor to the perfusate regulate the oncotic pressure and minimize edema formation (thus allowing higher pressure perfusions)?

RECOMMENDATION/STATEMENT: In ex situ normothermic machine perfusion for pancreatic transplantation, the addition of an oncotic factor to the perfusate regulate the oncotic pressure and minimize edema formation (thus allowing higher pressure perfusions).

Quality of evidence: Low – Expert opinion

Strength of recommendation: Strong for

Analysis of the literature:
M R Eloy et al: Homologous erythrocytes + culture medium + 1 g/L Earles' salts and glucose + 40 g/L bovine **albumin** + 50 g/L dextran.

PRELIMINARY DRAFT

Eckhauser F et al: Balanced electrolyte solution + **4 g/dL bovine serum albumin** + 150 mg/dL glucose + 12.5% **mannitol** + 1000 IU heparin + autologous red blood cells

Pegg DE et al: **Gelatin** polypeptide Haemaccel + 5.4 mM calcium + 5 mM glucose + 3 M glucose at one hour of perfusion Perfusion solution singlepass method: same solution with 5 mM glucose the first hour and solution with 30 mM for the remaining hour

Wahlberg J et al: UW solution + 5 g **dextran,** no hg

Mazilescu et al: Ringer's lactate, STEEN Solution (XVIVO Perfusion AB, Goteborg, Sweden), washed leukocyte-filtered erythrocytes, double reverse osmosis water, sodium bicarbonate, calcium gluconate, heparin, aprotinin.
(aprotinin, albumin)

Barlow et al. adapted their kidney ex situ NMP technique to initiate ex situ NMP of four non-transplantable pancreases. A red blood cell-based perfusate mixture was diluted with Gelofusine as colloid plus additives (sodium bicarbonate, mannitol, glucose, and heparin). All organs demonstrated adequate perfusion, pancreatic exocrine function, and insulin production. Histopathology demonstrated varying degrees of damage, reflective of the heterogeneous features of pancreas donor, ranging from focal to extended acinar necrosis.
Perfusate-containing packed red blood cells improve outcomes when compared to whole blood perfusate. Thus, a red blood cell-based perfusate solution with adequate oxygenation (95%) and supplemented with antiprotease, colloid, antibiotics and heparin may provide a prolonged preservation of the pancreas grafts. More studies are needed to assess the usefulness of other red blood cell-based perfusates combined with artificial acellular solutions.

Kuan and Kumar et al: No

Nassar: NS

References:
M R Eloy, J Kachelhoffer, A Pousse, J Dauchel, J F Grenier. Ex vivo vascular perfusion of the isolated canine pancreas. Experimental procedure, haemodynamic data and experimental applications. *Eur Surg Res*. **(1974)** 6(6):341-53. doi: 10.1159/000127741.

Eckhauser F, Knol JA, Porter-Fink V, Lockery D, Edgcomb L, Strodel WE, Webb D, Simmons J. Ex vivo normothermic hemoperfusion of the canine pancreas: applications and limitations of a modified experimental preparation. *J Surg Res*. **(1981)** 31(1):22-37. doi: 10.1016/0022-4804(81)90026-3.

Pegg DE, Klempnauer J, Diaper MP, Taylor MJ. Assessment of hypothermic preservation of the pancreas in the rat by a normothermic perfusion assay. *J Surg Res*. **(1982)** 33(3):194-200. doi: 10.1016/0022-4804(82)90029-4.

Wahlberg J, Southard JH, Belzer FO. Preservation-induced pancreatitis in an isolated perfused pancreas model in the dog. *Transpl Int.* **(1989)** 2(3):165-7. doi: 10.1007/BF02414603.

Kuan KG, Wee MN, Chung WY, Kumar R, Mees ST, Dennison A, et al. A Study of Normothermic Hemoperfusion of the Porcine Pancreas and Kidney. *Artif Organs*. **(2017)** 41(5):490-495. doi: 10.1111/aor.12770.

PRELIMINARY DRAFT

Kumar R, Chung WY, Runau F, Isherwood JD, Kuan KG, West K, et al. Ex vivo normothermic porcine pancreas: A physiological model for preservation and transplant study. *Int J Surg*. **(2018)** 54(Pt A):206-215. doi: 10.1016/j.ijsu.2018.04.057.

Mazilescu LI, Parmentier C, Kalimuthu SN, Ganesh S, Kawamura M, Goto T, et al. Normothermic ex situ pancreas perfusion for the preservation of porcine pancreas grafts. *Am J Transplant.* **(2022)** 22(5):1339-1349. doi: 10.1111/ajt.17019.

Barlow AD, Hamed MO, Mallon DH, Brais RJ, Gribble FM, Scott MA, et al. Use of Ex Vivo Normothermic Perfusion for Quality Assessment of Discarded Human Donor Pancreases. *Am J Transplant.* **(2015)** 15(9):2475-82. doi: 10.1111/ajt.13303.

Nassar A, Liu Q, Walsh M, Quintini C. Normothermic Ex Vivo Perfusion of Discarded Human Pancreas. *Artif Organs*. **(2018)** 42(3):334-335. doi: 10.1111/aor.12985.

PICO 6: In ex situ normothermic machine perfusion for pancreatic transplantation, does the addition of enzymatic inhibitor in the perfusate (i.e. aprotinin) reduce graft necrosis and allow for longer perfusions?

RECOMMENDATION/STATEMENT: In ex situ normothermic machine perfusion for pancreatic transplantation, the addition of enzymatic inhibitor in the perfusate (i.e. aprotinin) reduces graft necrosis and allow for longer perfusions

Quality of evidence: Low – Expert opinion

Strength of recommendation: Strong for

Analysis of the literature:
Mazilescu LI et al: The perfusate consisted of STEEN solution. The solution included human serum albumin which provides an optimal colloid osmotic pressure and dextran to preserve the endothelium from excessive leukocyte interaction. In addition, washed-leukocytes-filtered erythrocytes, sodium bicarbonate, heparin and aprotinin were added. Seven out of ten pancreas were subjected **to 6 hours** of ex situ NMP. The grafts showed stable perfusion parameters, active metabolism, homeostasis, and only mild graft injury assessed through histology.

M R Eloy et al: No

PRELIMINARY DRAFT

Eckhauser F et al: No
Pegg DE et al: No
Wahlberg J et al: No
Kuan and Kumar et al: No
Barlow et al: NS
Nassar et al: NS

References:

Mazilescu LI, Parmentier C, Kalimuthu SN, Ganesh S, Kawamura M, Goto T, et al. Normothermic ex situ pancreas perfusion for the preservation of porcine pancreas grafts. *Am J Transplant.* **(2022)** 22(5):1339-1349. doi: 10.1111/ajt.17019.

PRELIMINARY DRAFT

PICO 7: In pancreatic transplantation (whole organ transplantation), should ex situ normothermic machine perfusion be beneficial if the duration is more than 1 hour and less than 6 hours?

RECOMMENDATION/STATEMENT: In pancreatic transplantation (whole organ transplantation), ex situ normothermic machine perfusion should be beneficial if the duration is more than 1 hour and less than 6 hours.

Quality of evidence: Low – Expert opinion

Strength of recommendation: Strong for

Analysis of the literature:
M R Eloy et al: 90 min

Eckhauser F et al: 4h-5h

Pegg DE et al: 2h. Recirculation method: (n = 5) Pancreases preserved under recirculation method for two hours Single-pass method: (n = 40) Pancreases preserved under single-pass method for two hours

Wahlberg J et al: 2 Hours Group I (controls; n = 6): NP immediately after procurement Group IIA (n = 4): 24 hours of SCS and NP without allopurinol Group IIB (n = 6): 24 hours of SCS

and NP with allopurinol Group IIIA (n = 4): 48 hours of SCS and NP without allopurinol Group IIIB (n = 6): 48 hours of SCS and NP with allopurinol.

Kuan KG et al: 4 h for pancreas alone, 2 h for pancreas + kidney

Kumar R et al: 2-4 h (control group), 4 h (low pressure group)
Mazilescu LI et al: Seven out of ten pancreas were subjected **to 6 hours** of ex situ NMP. 6 h EVNMP, or 3h EVNMP +Tx

Barlow AD et al: 1-2 h

Nassar et al.: 6h (n=2) and 12 h (n=1), utilized a modified liver perfusion machine for three DBD donor human pancreases. Perfusion times were longer (6-12h) compared to Barlow et al. (1-2h) but CIT was much lower (4h vs 13h in the study of Barlow et al.). Results revealed endocrine function at both 6- and 12-hours through the determination of C-peptide production and by chromogranin staining. At the end of perfusion, histology showed well-preserved cell architecture without necrosis or hemorrhage in 2 out of 3 pancreases.

References:
M R Eloy, J Kachelhoffer, A Pousse, J Dauchel, J F Grenier. Ex vivo vascular perfusion of the isolated canine pancreas. Experimental procedure, haemodynamic data and experimental applications. *Eur Surg Res*. (1974) 6(6):341-53. doi: 10.1159/000127741.

Eckhauser F, Knol JA, Porter-Fink V, Lockery D, Edgcomb L, Strodel WE, Webb D, Simmons J. Ex vivo normothermic hemoperfusion of the canine pancreas: applications and limitations of a modified experimental preparation. *J Surg Res*. (1981) 31(1):22-37. doi: 10.1016/0022-4804(81)90026-3.

Pegg DE, Klempnauer J, Diaper MP, Taylor MJ. Assessment of hypothermic preservation of the pancreas in the rat by a normothermic perfusion assay. *J Surg Res*. (1982) 33(3):194-200. doi: 10.1016/0022-4804(82)90029-4.

Wahlberg J, Southard JH, Belzer FO. Preservation-induced pancreatitis in an isolated perfused pancreas model in the dog. *Transpl Int.* (1989) 2(3):165-7. doi: 10.1007/BF02414603.

Kuan KG, Wee MN, Chung WY, Kumar R, Mees ST, Dennison A, et al. A Study of Normothermic Hemoperfusion of the Porcine Pancreas and Kidney. *Artif Organs*. (2017) 41(5):490-495. doi: 10.1111/aor.12770.

Kumar R, Chung WY, Runau F, Isherwood JD, Kuan KG, West K, et al. Ex vivo normothermic porcine pancreas: A physiological model for preservation and transplant study. *Int J Surg*. (2018) 54(Pt A):206-215. doi: 10.1016/j.ijsu.2018.04.057.

Mazilescu LI, Parmentier C, Kalimuthu SN, Ganesh S, Kawamura M, Goto T, et al. Normothermic ex situ pancreas perfusion for the preservation of porcine pancreas grafts. *Am J Transplant.* (2022) 22(5):1339-1349. doi: 10.1111/ajt.17019.

Barlow AD, Hamed MO, Mallon DH, Brais RJ, Gribble FM, Scott MA, et al. Use of Ex Vivo Normothermic Perfusion for Quality Assessment of Discarded Human Donor Pancreases. *Am J Transplant.* (2015) 15(9):2475-82. doi: 10.1111/ajt.13303.

PRELIMINARY DRAFT

Nassar A, Liu Q, Walsh M, Quintini C. Normothermic Ex Vivo Perfusion of Discarded Human Pancreas. *Artif Organs*. (2018) 42(3):334-335. doi: 10.1111/aor.12985.

PRELIMINARY DRAFT

PICO 8: In pancreatic transplantation (whole organ transplantation), could ex situ normothermic machine perfusion be performed by continuous or pulsatile perfusion?

RECOMMENDATION/STATEMENT: In pancreatic transplantation (whole organ transplantation), ex situ normothermic machine perfusion can be performed by continuous or pulsatile perfusion

Quality of evidence: Low – Expert opinion

Strength of recommendation: Strong for

Analysis of the literature:
There are no studies comparing the use of pulsating or non-pulsating machines.

M R Eloy et al: pulsatile

Eckhauser F et al: pulsatile

Pegg DE et al: NS

Wahlberg J et al: NS

Kuan KG et al: SARNS 8000 extracorporeal roller pump (3M, St. Paul, MN, USA), Baby-RX venous reservoir and membrane oxygenator (Terumo, Ann Arbor, MI, USA), and water bath temperature regulator (pulsatile)

Kumar R et al: Affinity CP Centrifugal pump and Minimax Plus oxygenation System (Medtronic Inc., (Minneapolis, MN, USA) with a thermostatic water-based heat exchanger unit (non-pulsatile)

Mazilescu LI et al: S3 heart-lung machine and neonatal cardiopulmonary bypass equipment consisting of a centrifugal pump, an oxygenator (Sorin Group Inc., Markham, Canada) with an addition of heat exchanger (non-pulsatile)

Barlow AD et al: Customized pediatric cardiopulmonary bypass technology developed for kidney EVNMP (Medtronic Inc.) with an organ chamber, venous reservoir, Biopump 560 centrifugal blood pump, oxygenator, and a heat exchanger (Chalice Medical Ltd, United Kingdom) (non-pulsatile)
Nassar et al: Customized liver perfusion device. Centrifugal pump, oxygenator, and heater exchanger (Medtronic Inc.) with an added Sarns 8000 Roller Pump (Terumo, Ann Arbor, MI, USA) (non-pulsatile)

References:

M R Eloy, J Kachelhoffer, A Pousse, J Dauchel, J F Grenier. Ex vivo vascular perfusion of the isolated canine pancreas. Experimental procedure, haemodynamic data and experimental applications. *Eur Surg Res*. (1974) 6(6):341-53. doi: 10.1159/000127741.

Eckhauser F, Knol JA, Porter-Fink V, Lockery D, Edgcomb L, Strodel WE, Webb D, Simmons J. Ex vivo normothermic hemoperfusion of the canine pancreas: applications and limitations of a modified experimental preparation. *J Surg Res*. (1981) 31(1):22-37. doi: 10.1016/0022-4804(81)90026-3.

Pegg DE, Klempnauer J, Diaper MP, Taylor MJ. Assessment of hypothermic preservation of the pancreas in the rat by a normothermic perfusion assay. *J Surg Res*. (1982) 33(3):194-200. doi: 10.1016/0022-4804(82)90029-4.

Wahlberg J, Southard JH, Belzer FO. Preservation-induced pancreatitis in an isolated perfused pancreas model in the dog. *Transpl Int.* (1989) 2(3):165-7. doi: 10.1007/BF02414603.

Kuan KG, Wee MN, Chung WY, Kumar R, Mees ST, Dennison A, et al. A Study of Normothermic Hemoperfusion of the Porcine Pancreas and Kidney. *Artif Organs*. (2017) 41(5):490-495. doi: 10.1111/aor.12770.

Kumar R, Chung WY, Runau F, Isherwood JD, Kuan KG, West K, et al. Ex vivo normothermic porcine pancreas: A physiological model for preservation and transplant study. *Int J Surg*. (2018) 54(Pt A):206-215. doi: 10.1016/j.ijsu.2018.04.057.

Mazilescu LI, Parmentier C, Kalimuthu SN, Ganesh S, Kawamura M, Goto T, et al. Normothermic ex situ pancreas perfusion for the preservation of porcine pancreas grafts. *Am J Transplant.* (2022) 22(5):1339-1349. doi: 10.1111/ajt.17019.

Barlow AD, Hamed MO, Mallon DH, Brais RJ, Gribble FM, Scott MA, et al. Use of Ex Vivo Normothermic Perfusion for Quality Assessment of Discarded Human Donor Pancreases. *Am J Transplant.* (2015) 15(9):2475-82. doi: 10.1111/ajt.13303.

PRELIMINARY DRAFT

Nassar A, Liu Q, Walsh M, Quintini C. Normothermic Ex Vivo Perfusion of Discarded Human Pancreas. *Artif Organs*. (2018) 42(3):334-335. doi: 10.1111/aor.12985.

PRELIMINARY DRAFT

PICO 9: In case of prolonged perfusion, does ex situ normothermic machine perfusion require the management of exocrine secretions (dialysis membrane, duodenal diversion) to prevent the development of graft edema?

RECOMMENDATION/STATEMENT: In case of prolonged perfusion, ex situ normothermic machine perfusion requires the management of exocrine secretions (dialysis membrane, duodenal diversion) to prevent the development of graft edema.

Quality of evidence: Low – Expert opinion

Strength of recommendation: Strong for

Analysis of the literature:
M R Eloy et al: NS

Eckhauser F et al: Modified dialysis unit

Pegg DE et al: NS

Wahlberg J et al: NS

Kuan et al: Yes (addition of the porcine kidney in 2 of the animals)

Mazilescu LI et al A dialysis system was built in the circuit. Coming from the main reservoir as the starting and end point, the perfusion solution is driven by a centrifugal pump through an oxygenator. After oxygenation of the solution, the perfusate runs through an arterial filter for removal of any emboli or other debris and then the circuit splits into a 60 cm long line running to a dialyzer unit and a 72 cm long line going to the aorta of the graft. The solution that runs

through the dialyzer returns to the main reservoir through a 50 cm long tubing. The perfusate is drained through the portal vein back through a 37cm long tubing to the main reservoir.

Barlow AD et al: The small bowel mesentery was over-sewn and excess peri-pancreatic tissue removed. A wide-bore catheter was inserted into the distal duodenum and secured with a purse-string suture. The pancreas was weighed on completion of back-bench preparation. Prior to EVNP, the pancreas was flushed with cold Gelofusine (B. Braun, Sheffield, UK) and any obvious leaking vessels ligated with absorbable ligatures. The portal vein was left to drain freely back into the reservoir.

Nassar A et al: A 10 french catheter was placed in the duodenum to collect pancreatic and duodenal secretions.

Regarding the time of perfusion, it is challenging to set a range but one may consider that, when using longer periods of perfusion, the use of a dialysis circuit may help to reduce edema formation and recirculation of metabolic toxins.

References:
M R Eloy, J Kachelhoffer, A Pousse, J Dauchel, J F Grenier. Ex vivo vascular perfusion of the isolated canine pancreas. Experimental procedure, haemodynamic data and experimental applications. *Eur Surg Res*. (1974) 6(6):341-53. doi: 10.1159/000127741.

Eckhauser F, Knol JA, Porter-Fink V, Lockery D, Edgcomb L, Strodel WE, Webb D, Simmons J. Ex vivo normothermic hemoperfusion of the canine pancreas: applications and limitations of a modified experimental preparation. *J Surg Res*. (1981) 31(1):22-37. doi: 10.1016/0022-4804(81)90026-3.

Pegg DE, Klempnauer J, Diaper MP, Taylor MJ. Assessment of hypothermic preservation of the pancreas in the rat by a normothermic perfusion assay. *J Surg Res*. (1982) 33(3):194-200. doi: 10.1016/0022-4804(82)90029-4.

Wahlberg J, Southard JH, Belzer FO. Preservation-induced pancreatitis in an isolated perfused pancreas model in the dog. *Transpl Int.* (1989) 2(3):165-7. doi: 10.1007/BF02414603.

Kuan KG, Wee MN, Chung WY, Kumar R, Mees ST, Dennison A, et al. A Study of Normothermic Hemoperfusion of the Porcine Pancreas and Kidney. *Artif Organs*. (2017) 41(5):490-495. doi: 10.1111/aor.12770.

Kumar R, Chung WY, Runau F, Isherwood JD, Kuan KG, West K, et al. Ex vivo normothermic porcine pancreas: A physiological model for preservation and transplant study. *Int J Surg*. (2018) 54(Pt A):206-215. doi: 10.1016/j.ijsu.2018.04.057.

Mazilescu LI, Parmentier C, Kalimuthu SN, Ganesh S, Kawamura M, Goto T, et al. Normothermic ex situ pancreas perfusion for the preservation of porcine pancreas grafts. *Am J Transplant.* (2022) 22(5):1339-1349. doi: 10.1111/ajt.17019.

Barlow AD, Hamed MO, Mallon DH, Brais RJ, Gribble FM, Scott MA, et al. Use of Ex Vivo Normothermic Perfusion for Quality Assessment of Discarded Human Donor Pancreases. *Am J Transplant.* (2015) 15(9):2475-82. doi: 10.1111/ajt.13303.

PRELIMINARY DRAFT

Nassar A, Liu Q, Walsh M, Quintini C. Normothermic Ex Vivo Perfusion of Discarded Human Pancreas. *Artif Organs*. (2018) 42(3):334-335. doi: 10.1111/aor.12985.

PRELIMINARY DRAFT

PICO 10: Are pancreatic grafts metabolically active during perfusion as evidenced by measuring oxygen extraction?

RECOMMENDATION/STATEMENT: Pancreatic grafts are metabolically active during perfusion as evident by measuring oxygen extraction.

Quality of evidence: Low – Expert opinion

Strength of recommendation: Strong for

Analysis of the literature:
M R Eloy et al: Stable oxygen consumption during perfusion time. 0,5-0,6 mL O2/min/100g

Eckhauser F et al: Stable oxygen consumption during perfusion time. 0,05 mL O2/min O2 consumption appeared to be a reliable measure of organ viability.

Wahlberg J et al: No oxygen measures

Pegg DE et al: No oxygen measures

Kuan KG et al: No oxygen measures

Kumar R et al: All grafts demonstrated an arterial to venous oxygen differential and in the presence of positive cells stained for active form of an enzyme involved with ATP production it is reasonable to argue that the oxygen uptake by grafts was being utilized at a cellular level, adding further support to the belief that this is a viable and physiological graft. Measurements: partial pressures of Oxygen sampled from the arterial inflow at the aortic segment (PaO2) and the venous outflow, sampled at the portal vein (PvO2).

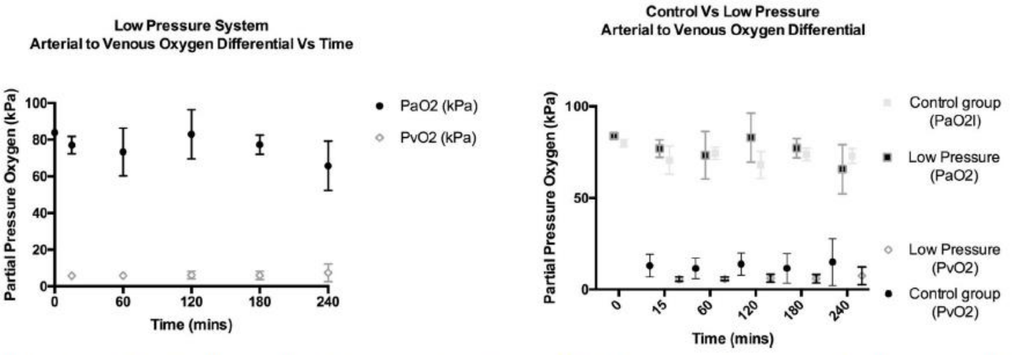

Fig. 4. Partial pressures of Oxygen sampled from the arterial inflow at the aortic segment (PaO_2) and the venous outflow, sampled at the portal vein (PvO_2). Error bars represent the 95% confidence interval at each time point during the perfusion. The low pressure system demonstrated a significant arterial to venous oxygen differential ($p < 0.0001$). The arterial to venous differential did not significantly differ when the control group was compared with the low pressure system (right).

Mazilescu LI et al: Oxygen consumption was constant over the course of perfusion, suggesting metabolic activity of the pancreas. Measurement: arterial, partial pressure of oxygen (aprox 600 pO2 mmHg) and venous, partial pressure of oxygen (aprox 400 pO2 mmHg).

1344 AJT MAZILESCU ET AL.

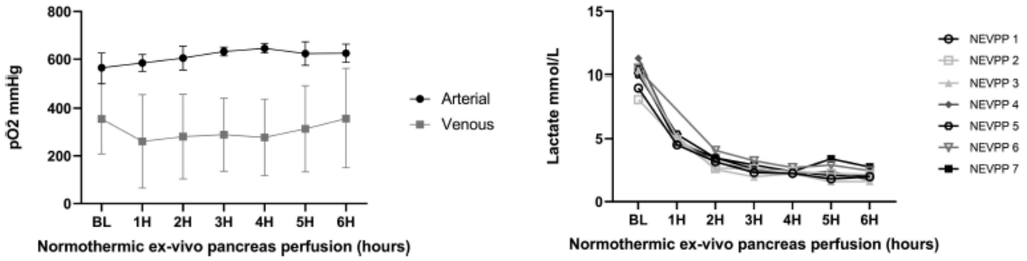

FIGURE 3 Oxygen partial pressure and lactate levels over the course of perfusion. BL, baseline; Arterial, partial pressure of oxygen in the arterial blood gas analysis; Venous, partial pressure of oxygen in the venous blood gas analysis; NESPP 1 – HNESPP 7, individual curves for each of the 7 cases. Oxygen partial pressure values are presented as mean ± SD

Barlow AD et al: No oxygen measures

Nassar A et al: No oxygen measures

References:

M R Eloy, J Kachelhoffer, A Pousse, J Dauchel, J F Grenier. Ex vivo vascular perfusion of the isolated canine pancreas. Experimental procedure, haemodynamic data and experimental applications. *Eur Surg Res*. (1974) 6(6):341-53. doi: 10.1159/000127741.

Eckhauser F, Knol JA, Porter-Fink V, Lockery D, Edgcomb L, Strodel WE, Webb D, Simmons J. Ex vivo normothermic hemoperfusion of the canine pancreas: applications and limitations of a modified experimental preparation. *J Surg Res*. (1981) 31(1):22-37. doi: 10.1016/0022-4804(81)90026-3.

Pegg DE, Klempnauer J, Diaper MP, Taylor MJ. Assessment of hypothermic preservation of the pancreas in the rat by a normothermic perfusion assay. *J Surg Res*. (1982) 33(3):194-200. doi: 10.1016/0022-4804(82)90029-4.

Wahlberg J, Southard JH, Belzer FO. Preservation-induced pancreatitis in an isolated perfused pancreas model in the dog. *Transpl Int.* (1989) 2(3):165-7. doi: 10.1007/BF02414603.

Kuan KG, Wee MN, Chung WY, Kumar R, Mees ST, Dennison A, et al. A Study of Normothermic Hemoperfusion of the Porcine Pancreas and Kidney. *Artif Organs*. (2017) 41(5):490-495. doi: 10.1111/aor.12770.

Kumar R, Chung WY, Runau F, Isherwood JD, Kuan KG, West K, et al. Ex vivo normothermic porcine pancreas: A physiological model for preservation and transplant study. *Int J Surg*. (2018) 54(Pt A):206-215. doi: 10.1016/j.ijsu.2018.04.057.

Mazilescu LI, Parmentier C, Kalimuthu SN, Ganesh S, Kawamura M, Goto T, et al. Normothermic ex situ pancreas perfusion for the preservation of porcine pancreas grafts. *Am J Transplant.* (2022) 22(5):1339-1349. doi: 10.1111/ajt.17019.

Barlow AD, Hamed MO, Mallon DH, Brais RJ, Gribble FM, Scott MA, et al. Use of Ex Vivo Normothermic Perfusion for Quality Assessment of Discarded Human Donor Pancreases. *Am J Transplant.* (2015) 15(9):2475-82. doi: 10.1111/ajt.13303.

Nassar A, Liu Q, Walsh M, Quintini C. Normothermic Ex Vivo Perfusion of Discarded Human Pancreas. *Artif Organs*. (2018) 42(3):334-335. doi: 10.1111/aor.12985.

PICO 11: During ex situ normothermic machine perfusion for pancreatic transplantation, could the endocrine function of the pancreas graft be assessed by insulin secretion tests (under glucose or arginine stimulation)?

RECOMMENDATION/STATEMENT: During ex situ normothermic machine perfusion for pancreatic transplantation, the endocrine function of the pancreas graft can be assessed by insulin secretion tests (under glucose or arginine stimulation)

Quality of evidence: Low – Expert opinion

Strength of recommendation: Strong for

Analysis of the literature:
M R Eloy et al: Endocrine function under glucose stimulation

Eckhauser F et al: No

Wahlberg J et al: NS

Pegg DE et al: Endocrine function under glucose stimulation
Gelatin polypeptide Haemaccel + 5.4 mM calcium + 5 mM glucose + 3 M glucose at one hour of perfusion Perfusion solution singlepass method: same solution with 5 mM glucose the first hour and solution with 30 mM for the remaining hour
95% oxygen, 5%carbon dioxide
Recirculation method: WG: wet/dry weight ratio = 8.2 ± 0.8 Single-pass method: WG: wet/dry weight ratio = 6.6 ± 0.3. ded crease of resistance during the first 2 hours.

PRELIMINARY DRAFT

Insuline respond to high glucose but not respond to mannitol injection. e addition of 50 mM mannitol to the basic preservation solution significantly reduced insulin release during the first hour (PPI vs PP2, P = 0.05) although it had no significant effect on the responseto a glucose challenge.

Kuan KG et al: No

Kumar R et al: Endocrine function under glucose stimulation
Measure of hemoglobin, lactate, glucose, potassium, sodium and chloride

Mazilescu LI et al: LDH, Glucose, insulin, C-peptide and bicarbonate concentration
Inflammatory cytokines
Mild hemolysis but possible assessment of amylase
Endocrine function under glucose stimulation

Barlow AD et al: Endocrine function under glucose stimulation
Gross appearance
Hematoxylin and eosin stain in biopsies

Nassar A et al: C-peptide production
Hematoxylin and eosin stain of pancreas acinus and islets

References:
M R Eloy, J Kachelhoffer, A Pousse, J Dauchel, J F Grenier. Ex vivo vascular perfusion of the isolated canine pancreas. Experimental procedure, haemodynamic data and experimental applications. *Eur Surg Res*. (1974) 6(6):341-53. doi: 10.1159/000127741.

Eckhauser F, Knol JA, Porter-Fink V, Lockery D, Edgcomb L, Strodel WE, Webb D, Simmons J. Ex vivo normothermic hemoperfusion of the canine pancreas: applications and limitations of a modified experimental preparation. *J Surg Res*. (1981) 31(1):22-37. doi: 10.1016/0022-4804(81)90026-3.

Pegg DE, Klempnauer J, Diaper MP, Taylor MJ. Assessment of hypothermic preservation of the pancreas in the rat by a normothermic perfusion assay. *J Surg Res*. (1982) 33(3):194-200. doi: 10.1016/0022-4804(82)90029-4.

Wahlberg J, Southard JH, Belzer FO. Preservation-induced pancreatitis in an isolated perfused pancreas model in the dog. *Transpl Int.* (1989) 2(3):165-7. doi: 10.1007/BF02414603.

Kuan KG, Wee MN, Chung WY, Kumar R, Mees ST, Dennison A, et al. A Study of Normothermic Hemoperfusion of the Porcine Pancreas and Kidney. *Artif Organs*. (2017) 41(5):490-495. doi: 10.1111/aor.12770.

Kumar R, Chung WY, Runau F, Isherwood JD, Kuan KG, West K, et al. Ex vivo normothermic porcine pancreas: A physiological model for preservation and transplant study. *Int J Surg*. (2018) 54(Pt A):206-215. doi: 10.1016/j.ijsu.2018.04.057.

Mazilescu LI, Parmentier C, Kalimuthu SN, Ganesh S, Kawamura M, Goto T, et al. Normothermic ex situ pancreas perfusion for the preservation of porcine pancreas grafts. *Am J Transplant.* (2022) 22(5):1339-1349. doi: 10.1111/ajt.17019.

PRELIMINARY DRAFT

Barlow AD, Hamed MO, Mallon DH, Brais RJ, Gribble FM, Scott MA, et al. Use of Ex Vivo Normothermic Perfusion for Quality Assessment of Discarded Human Donor Pancreases. *Am J Transplant.* (2015) 15(9):2475-82. doi: 10.1111/ajt.13303.

Nassar A, Liu Q, Walsh M, Quintini C. Normothermic Ex Vivo Perfusion of Discarded Human Pancreas. *Artif Organs*. (2018) 42(3):334-335. doi: 10.1111/aor.12985.

PICO 12: During ex situ normothermic machine perfusion for pancreatic transplantation, could preservation of pancreatic exocrine function be assessed by amylase and lipase levels in the perfusate/pancreatic juice?

RECOMMENDATION/STATEMENT: During ex situ normothermic machine perfusion for pancreatic transplantation, preservation of pancreatic exocrine function can be assessed by amylase and lipase levels in the perfusate/pancreatic juice

Quality of evidence: Low – Expert opinion

Strength of recommendation: Strong for

Analysis of the literature:
M R Eloy et al: Glucose, insuline, secretin, cholescystokinina. No amylase and lipase

Eckhauser F et al: amylase and lipase in pancreatic juice

Wahlberg J et al: Amylase in perfusate

Pegg DE et al: insuline. No amylase and lipase

Kuan KG et al: No

Kumar R et al: Exocrine function in perfusate through amylase and lipase levels and production of pancreatic juice. Measure of hemoglobin, lactate, glucose, potassium, sodium and chloride

Mazilescu et al: Sample collection: Blood gas analyses of the perfusate were performed hourly during graft perfusion. Additionally, blood gas analyses of the donor were taken before retrieval and blood gas analyses of the recipient were taken before pancreas retrieval, before and after transplantation and every day during postoperative care. Samples were also analyzed using a point-of- care comprehensive metabolic blood chemistry analyzer (Piccolo Xpress, Union City, Canada) and part of each sample was stored at −80°C for later analysis. For amylase, lipase and lactate dehydrogenase (LDH) measurements, samples were sent to the Toronto General Hospital Core Laboratory for analysis with the Abbott Architect Chemistry Analyzer using the manufacturer's reagents. For the grafts that only received NESPP, after 4 hours of perfusion, a glucose tolerance test was performed by adding 1ml of Dextrose 50% (Baxter Corporation, Mississauga, Canada) to the perfusate. Glucose, insulin, and c-peptide

PRELIMINARY DRAFT

levels were measured at 1, 5, and 10 min after the glucose administration. For measurement of insulin and c-peptide enzyme linked immunosorbent assays kit (R&D Systems, Toronto, Canada and Mercodia, Winston Salem, United States) were used according to manufacturer's instructions. For the grafts that were transplanted, no glucose test was performed during NESPP. A glucose test was performed at 48–72 h after transplantation, by administrating 50 ml of Dextrose 50% (Baxter Corporation, Mississauga, Canada) to the recipient animals. Glucose levels were monitored for two hours and samples were taken at multiple timepoints.

Barlow AD et al: amylase and lipase in perfusate

Nassar A et al: Exocrine evaluation through bicarbonate levels in the duodenum.

References:

M R Eloy, J Kachelhoffer, A Pousse, J Dauchel, J F Grenier. Ex vivo vascular perfusion of the isolated canine pancreas. Experimental procedure, haemodynamic data and experimental applications. *Eur Surg Res*. (1974) 6(6):341-53. doi: 10.1159/000127741.

Eckhauser F, Knol JA, Porter-Fink V, Lockery D, Edgcomb L, Strodel WE, Webb D, Simmons J. Ex vivo normothermic hemoperfusion of the canine pancreas: applications and limitations of a modified experimental preparation. *J Surg Res*. (1981) 31(1):22-37. doi: 10.1016/0022-4804(81)90026-3.

Pegg DE, Klempnauer J, Diaper MP, Taylor MJ. Assessment of hypothermic preservation of the pancreas in the rat by a normothermic perfusion assay. *J Surg Res*. (1982) 33(3):194-200. doi: 10.1016/0022-4804(82)90029-4.

Wahlberg J, Southard JH, Belzer FO. Preservation-induced pancreatitis in an isolated perfused pancreas model in the dog. *Transpl Int.* (1989) 2(3):165-7. doi: 10.1007/BF02414603.

Kuan KG, Wee MN, Chung WY, Kumar R, Mees ST, Dennison A, et al. A Study of Normothermic Hemoperfusion of the Porcine Pancreas and Kidney. *Artif Organs*. (2017) 41(5):490-495. doi: 10.1111/aor.12770.

Kumar R, Chung WY, Runau F, Isherwood JD, Kuan KG, West K, et al. Ex vivo normothermic porcine pancreas: A physiological model for preservation and transplant study. *Int J Surg*. (2018) 54(Pt A):206-215. doi: 10.1016/j.ijsu.2018.04.057.

Mazilescu LI, Parmentier C, Kalimuthu SN, Ganesh S, Kawamura M, Goto T, et al. Normothermic ex situ pancreas perfusion for the preservation of porcine pancreas grafts. *Am J Transplant.* (2022) 22(5):1339-1349. doi: 10.1111/ajt.17019.

Barlow AD, Hamed MO, Mallon DH, Brais RJ, Gribble FM, Scott MA, et al. Use of Ex Vivo Normothermic Perfusion for Quality Assessment of Discarded Human Donor Pancreases. *Am J Transplant.* (2015) 15(9):2475-82. doi: 10.1111/ajt.13303.

Nassar A, Liu Q, Walsh M, Quintini C. Normothermic Ex Vivo Perfusion of Discarded Human Pancreas. *Artif Organs*. (2018) 42(3):334-335. doi: 10.1111/aor.12985.

PRELIMINARY DRAFT

PRELIMINARY DRAFT

PICO 13: Should ex situ normothermic machine perfusion for pancreatic transplantation be performed simultaneously through the superior mesenteric artery and the splenic artery?

RECOMMENDATION/STATEMENT: Ex situ normothermic machine perfusion for pancreatic transplantation must be performed simultaneously through the superior mesenteric artery and the splenic artery

Quality of evidence: Low – Expert opinion

Strength of recommendation: Strong for

Analysis of the literature:
M R Eloy et al: Cannulation of the aorta (CT and SMA)

Eckhauser F et al: Cannulation of the aorta

Wahlberg J et al: Splenic artery and portal vein cannulated

Pegg DE et al: NS

Kuan KG et al: The aorta and portal vein were then cannulated with 24-Fr catheters and secured. A separate cannula was inserted proximal to the cannulated portion of the abdominal aorta for collection of blood.

Kumar R et al: Cannulation of the aorta

Mazilescu LI et al: On the back-table, all arterial branches were tied off and spleen was removed. Next, the portal vein and aorta were cannulated using 3/8" x 1/4" and 1/4" x 3/8" cannulas. The pancreatic duct and duodenum were also cannulated to allow for output measurements during perfusion. The pancreas was then placed in an organ bag and stored on ice until the perfusion was started. Shortly prior to connecting the pancreas to the ex situ system, the organ was flushed with 500ml Albumin.

Barlow AD et al: Arterial reconstruction was performed with a donor iliac Y graft to the splenic and superior mesenteric arteries using 5-0 polypropylene sutures. An arterial cannula was secured in the common iliac portion of the Y graft.

Nassar A et al: The superior mesenteric artery (SMA) and celiac trunk were cannulated for the arterial inflow. The portal vein drained freely into the organ receptacle.

References:

M R Eloy, J Kachelhoffer, A Pousse, J Dauchel, J F Grenier. Ex vivo vascular perfusion of the isolated canine pancreas. Experimental procedure, haemodynamic data and experimental applications. *Eur Surg Res*. (1974) 6(6):341-53. doi: 10.1159/000127741.

Eckhauser F, Knol JA, Porter-Fink V, Lockery D, Edgcomb L, Strodel WE, Webb D, Simmons J. Ex vivo normothermic hemoperfusion of the canine pancreas: applications and limitations of a modified experimental preparation. *J Surg Res*. (1981) 31(1):22-37. doi: 10.1016/0022-4804(81)90026-3.

Pegg DE, Klempnauer J, Diaper MP, Taylor MJ. Assessment of hypothermic preservation of the pancreas in the rat by a normothermic perfusion assay. *J Surg Res*. (1982) 33(3):194-200. doi: 10.1016/0022-4804(82)90029-4.

Wahlberg J, Southard JH, Belzer FO. Preservation-induced pancreatitis in an isolated perfused pancreas model in the dog. *Transpl Int.* (1989) 2(3):165-7. doi: 10.1007/BF02414603.

Kuan KG, Wee MN, Chung WY, Kumar R, Mees ST, Dennison A, et al. A Study of Normothermic Hemoperfusion of the Porcine Pancreas and Kidney. *Artif Organs*. (2017) 41(5):490-495. doi: 10.1111/aor.12770.

Kumar R, Chung WY, Runau F, Isherwood JD, Kuan KG, West K, et al. Ex vivo normothermic porcine pancreas: A physiological model for preservation and transplant study. *Int J Surg*. (2018) 54(Pt A):206-215. doi: 10.1016/j.ijsu.2018.04.057.

Mazilescu LI, Parmentier C, Kalimuthu SN, Ganesh S, Kawamura M, Goto T, et al. Normothermic ex situ pancreas perfusion for the preservation of porcine pancreas grafts. *Am J Transplant.* (2022) 22(5):1339-1349. doi: 10.1111/ajt.17019.

Barlow AD, Hamed MO, Mallon DH, Brais RJ, Gribble FM, Scott MA, et al. Use of Ex Vivo Normothermic Perfusion for Quality Assessment of Discarded Human Donor Pancreases. *Am J Transplant.* (2015) 15(9):2475-82. doi: 10.1111/ajt.13303.

Nassar A, Liu Q, Walsh M, Quintini C. Normothermic Ex Vivo Perfusion of Discarded Human Pancreas. *Artif Organs*. (2018) 42(3):334-335. doi: 10.1111/aor.12985.

Mazilescu LI, Parmentier C, Kalimuthu SN, Ganesh S, Kawamura M, Goto T, et al. Normothermic ex situ pancreas perfusion for the preservation of porcine pancreas grafts. *Am J Transplant.* (2022) 22(5):1339-1349. doi: 10.1111/ajt.17019.

PRELIMINARY DRAFT

2- ISLET TRANSPLANTATION

EX-SITU NORMOTHERMIC MACHINE PERFUSION (NMP)

Given the absence of references to the ex situ use of NMP in islet transplantation, the same PICO questions have been raised as in whole pancreas transplantation. In this sense, we consider that the recommendations can be extrapolated but with a very low level of evidence.

Quality of evidence: very low

Strength of recommendation: strong for

PICO QUESTIONS

1. Could ex situ normothermic machine perfusion be a reliable method for evaluating (functional assessment) explanted pancreas after static cold preservation in islet transplantation?
2. In islet transplantation, should ex situ normothermic machine perfusion be performed at physiologic temperature, with perfusate solution containing an oxygen carrier to sustain metabolic activities of the cells?
3. In islet transplantation, should ex situ normothermic machine perfusion be performed at a pressure range from 25 to 50 mmHg?
4. In islet transplantation, does ex situ normothermic machine perfusion require a balance of pressure and flow to ensure minimal damage to the endothelium?
5. In ex situ normothermic machine perfusion for islet transplantation, does the addition of an oncotic factor to the perfusate regulate the oncotic pressure and minimize edema formation (thus allowing higher pressure perfusions)?
6. In ex situ normothermic machine perfusion for islet transplantation, does the addition of enzymatic inhibitor in the perfusate (i.e. aprotinin) reduce graft necrosis and allow for longer perfusions?
7. In islet transplantation, should ex situ normothermic machine perfusion be beneficial if the duration is more than 1 hour and less than 6 hours?
8. In islet transplantation, could ex situ normothermic machine perfusion be performed by continuous or pulsatile perfusion?
9. In case of prolonged perfusion, does ex situ normothermic machine perfusion require the management of exocrine secretions (dialysis membrane, duodenal diversion) to prevent the development of graft edema?
10. Are pancreatic grafts metabolically active during perfusion as evidenced by measuring oxygen extraction?
11. During ex situ normothermic machine perfusion for islet transplantation, could the endocrine function of the pancreas graft be assessed by insulin secretion tests (under glucose or arginine stimulation)?
12. During ex situ normothermic machine perfusion for islet transplantation, could preservation of pancreatic exocrine function be assessed by amylase and lipase levels in the perfusate/pancreatic juice?
13. Should ex situ normothermic machine perfusion for islet transplantation be performed simultaneously through the superior mesenteric artery and the splenic artery?

PRELIMINARY DRAFT

1- PANCREATIC TRANSPLANTATION (WHOLE ORGAN TRANSPLANTATION)
IN-SITU NORMOTHERMIC REGIONAL PERFUSION (NRP)

PICO QUESTIONS

1. Is normothermic regional perfusion a reliable and reproducible method for donation after controlled circulatory death in the scenario of whole pancreas transplantation?
2. For whole pancreas transplantation, is normothermic regional perfusion compatible with the procurement of other abdominal (kidneys, liver) organs?
3. For whole pancreas transplantation, is normothermic regional perfusion compatible with the procurement of other thoracic (heart, lungs) organs?
4. Should post-mortem normothermic regional perfusion be run for a maximum of 3 hours in the context of whole pancreas transplantation?
5. Should valid parameters (machine perfusion -monitoring flow and temperature-, analytical/biochemical parameters and functional warm ischemia time) be defined to assess the quality of the pancreatic graft before deciding the suitability/validity of the organ for whole pancreas transplant?
6. Could normothermic regional perfusion in donation after circulatory death improve graft and patient outcomes compared with in situ cooling and rapid procurement in pancreas transplantation?
7. Does the normothermic regional perfusion have the potential to expand the donor pool for whole pancreas transplantation?

PRELIMINARY DRAFT

PICO 1: Is normothermic regional perfusion a reliable and reproducible method for donation after controlled circulatory death in the scenario of whole pancreas transplantation?

RECOMMENDATION/STATEMENT: Normothermic regional perfusion is a reliable and reproducible method for donation after controlled circulatory death in the scenario of whole pancreas transplantation.

Quality of evidence: Moderate

Strength of recommendation: Strong for

Analysis of the literature:
A total of nine studies reporting outcome after cDCD pancreas transplantation using normothermic regional perfusion have been published so far.
Other series using NRP in cDCD pancreas transplantation were presented as an abstract*, as shown below.

Oniscu GC et al, 2022 (CASE SERIES, n= 31, 28 SPK)
Oniscu et al published a retrospective analysis of UK adult cDCD donors' where at least 1 abdominal organ was accepted for transplantation between January 1, 2011, and December 31, 2019.
There were 1906 donors who had their pancreas offered, of which 413 (22%) proceeded to transplant. Thirty-one NRP pancreases were transplanted (28 as SPK, 1 as kidney and pancreas islets, and 2 as pancreas islets). Three-hundred eighty-two non-NRP pancreases were transplanted (312 as SKP, 3 as kidney and pancreas islet, 41 as pancreas alone, and 26 as pancreas islets).
The use of NRP influenced the probability of pancreas transplantation (SPK) with a 1.6 odds ratio for donors undergoing NRP compared with those who did not (LRT P = 0.0611).

Richards JA et al, 2021 (CASE SERIES, n=13)
A total of 211 patients (139 DBD and 72 DCD, of which 59 were sDCD and 13 normothermic regional perfusion were included.
In this series of DCD simultaneous pancreas and kidney transplantation, long-term follow-up data demonstrate that patient and graft survival are equivalent for sDCD and DBD organs with no difference in graft function at 1 year. The findings indicate that pancreas transplantation following normothermic regional perfusion is both feasible and offers comparable outcomes.

Miñambres E et al, 2017 (CASE SERIES, n=1)
This is a single-center retrospective review of all cDCD procedures from the start of the program in September 2014 to September 2016. A total of 27 cDCD donors underwent abdominal nRP. As controls, 51 DBD donors were evaluated. A total of 37 kidneys, 11 livers, six bilateral lungs, and one pancreas obtained from cDCD donors were finally transplanted.
Simultaneous kidney–pancreas transplantation was performed with appropriate graft function after 6-month follow-up.

Oniscu GC et al, 2014 (CASE SERIES, n=5, 2 SPK).
Five pancreata were recovered from these donors. Two simultaneous pancreas–kidney transplants were performed in type I diabetic patients aged 52 and 34 years old, with primary renal and pancreatic function.. Two pancreata were sent for islet isolation. One achieved a good islet yield and was transplanted while the second one was fibrotic and achieved a lower

PRELIMINARY DRAFT

yield than required for transplantation. The fifth pancreas was initially accepted for transplantation as a solid organ by two centers but later turned down on logistic reasons and was eventually sent for research. The remainder of the pancreata were not recovered (three citing a long WIT, eight donor age and five donor history as the reasons for non use).

Butler AJ et al, 2014 (CASE SERIES, n=2)
Eight controlled DCD donors underwent NRP from which 3 livers, 2 pancreases, and 14 kidneys were transplanted. Four livers were not used because of biochemical evidence of hepatocellular damage and one because of cirrhosis. Two kidneys were lost from venous thrombosis before function returned and two developed delayed graft function; all transplanted livers and pancreases had primary function.
Conclusions: Cannulation and heparinization after circulatory arrest does not prevent successful normothermic regional perfusion. The technique permits evaluation of donor organs before implantation and may improve short-term outcomes.
This paper describes our early experience with normothermic regional perfusion. It enabled assessment of donor livers and has the potential to improve the early outcomes of other organs retrieved from controlled DCD donors without the need for prior heparinization or cannulation.

Magliocca JF et al (CASE SERIES, n=1)
Twenty patients were enrolled in DCD protocol and underwent attempted organ donation. Fifteen patients completed the protocol; 3 maintained cardiac function throughout the prescribed 60 minutes after withdrawal of life support, and two patients' organs were deemed unsuitable for transplantation. Fourteen (70%) of the DCD donor patients originated on the trauma service and six (30%) were from other clinical services. The DCD program increased the potential donor pool by 33% (61 versus 81 patients) and the number of kidneys transplanted by 24% (100 versus 124). A total of 24 kidney, 5 liver, and 1 pancreas transplants were performed with these organs. Two of 24 (8.3%) DCD kidneys had delayed graft function. There were no perioperative rejection episodes or deaths.

Farney AC et al. (CASE SERIES; n=4)
From April 1, 2003, to October 3, 2007, 53 kidney transplantations and 4 simultaneous kidney-pancreas transplantations from DCD donors were performed.
All DCD donor kidneys were managed with pulsatile perfusion preservation, and all simultaneous kidney-pancreas transplantation donors were managed with extracorporeal support.
Patient- and graft-survival rates were 100% in the 4 simultaneous kidney-pancreas transplantations.

Rojas-Peña A, et al. (CASE SERIES, n=1)
A retrospective review of all potential controlled-DCDD cases between October 1, 2000 and July 31, 2013 was performed.
Extracorporeal support (ECS) was used for organ procurement in 37 controlled DCDD. The number of organs procured per donor was 2.59, and the number of organs transplanted per donor was 1.68. Delayed graft function occurred in 31% of renal grafts. In three donors (8%), organ donation was not completed because of surgeon judgment. Forty-eight renal grafts (65.8%), thirteen livers (61.9%), and one pancreas (50%) were successfully transplanted.

Mesnard B et al (CASE SERIES, n=1) No abstract. French
Perfusion duration: 3 h. Getinge. 37ºC. Whole blood
Splenic thrombosis

PRELIMINARY DRAFT

References:

Oniscu GC, Mehew J, Butler AJ, Sutherland A, Gaurav R, Hogg R, Currie I, Jones M, Watson CJE. Improved Organ Utilization and Better Transplant Outcomes with In Situ Normothermic Regional Perfusion in Controlled Donation After Circulatory Death. Transplantation. 2022 Aug 22. doi: 10.1097/TP.0000000000004280. Epub ahead of print. PMID: 35993664.

Richards JA, Roberts JL, Fedotovs A, Paul S, Cottee S, Defries G, et al. Outcomes for circulatory death and brainstem death pancreas transplantation with or without use of normothermic regional perfusion. British Journal of Surgery. 2021;108(12):1406-8.

Minambres E, Suberviola B, Dominguez-Gil B, Rodrigo E, Ruiz-San Millan JC, Rodriguez-San Juan JC, et al. Improving the Outcomes of Organs Obtained From Controlled Donation After Circulatory Death Donors Using Abdominal Normothermic Regional Perfusion. American Journal of Transplantation. 2017;17(8):2165-72.

Oniscu GC, Randle LV, Muiesan P, Butler AJ, Currie IS, Perera MT, et al. In situ normothermic regional perfusion for controlled donation after circulatory death--the United Kingdom experience. American Journal of Transplantation. 2014;14(12):2846-54.

Butler AJ, Randle LV, Watson CJ. Normothermic regional perfusion for donation after circulatory death without prior heparinization. Transplantation. 2014;97(12):1272-8.

Magliocca JF, Magee JC, Rowe SA, Gravel MT, Chenault RH, 2nd, Merion RM, et al. Extracorporeal support for organ donation after cardiac death effectively expands the donor pool. J Trauma. 2005;58(6):1095-101; discussion 101-2.

Farney AC, Singh RP, Hines MH, Rogers J, Hartmann EL, Reeves-Daniel A, Gautreaux MD, Iskandar SS, Adams PL, Stratta RJ. Experience in renal and extrarenal transplantation with donation after cardiac death donors with selective use of extracorporeal support. J Am Coll Surg. 2008 May;206(5):1028-37; discussion 1037. doi: 10.1016/j.jamcollsurg.2007.12.029. Epub 2008 Mar 17. PMID: 18471749.

Rojas-Peña A, Sall LE, Gravel MT, et al. Donation after circulatory determination of death: the university of Michigan experience with extracorporeal support. Transplantation. 2014;98:328–334.

Mesnard B, Cantarovich D, Martin-Lefevre L, Rigaud J, Blancho G, Karam G, Badet L, Antoine C, Branchereau J. First French combined kidney/pancreas transplantation from controlled donation after circulatory arrest (Maastricht III). Prog Urol. 2022 Jan;32(1):1-2. doi: 10.1016/j.purol.2021.10.001. Epub 2021 Nov 10. PMID: 34772617.

***ABSTRACT**

Ferrer, J., et al. (2021). "Pancreas transplantation from donors declared death by circulatory criteria: Initial experience in Spain." Transplant International 34(SUPPL 1): 173-174.

> Background: In the face of the shortage of organs for transplantation, the transplant community is increasingly considering controlled donation after the determination of death by circulatory criteria (cDCDD). There is a scarcity of studies concerning the use of Normothermic Regional Perfusion (NRP), an in situ preservation strategy well established in Spain. Aim(s): To report on the Spanish experience on the outcomes obtained from cDCDD donors. Method(s): Data from the Spanish National Transplant Organization database and from transplant centers were

PRELIMINARY DRAFT

retrospectively analyzed (2015- 2020). Result(s): During the study period, 471 pancreas transplants were performed, including 20 combined kidney-pancreas transplants from cDCDD donors. Of these, NRP was used in 18 procedures, all with ante-mortem cannulation, and rapid recovery (RR) in 2 cases. The median donor age was 33 years, 65% were male. The median total warm ischemia time (WIT) and the functional WIT were 19 (13.2-23.7) min. and 10 (7-15.5) min., respectively. Postmortem NRP was run for a 113.5 (91.5-134.5) min. The median pancreas cold ischemia was 412.5 (330- 636.7) min. The pancreatic graft function was optimal in all cases except for three (NRP cases), for which the cause was primary nonfunction in two (one of them requiring transplantectomy of pancreatic and kidney grafts) and cardiogenic and septic shock secondary to pancreatic fistula for a fatal case. Seven patients presented with delayed kidney graft function, with five cases requiring dialysis. Pancreas related surgical complications were present in 70% cases, haemorrhage being the most common. After a median follow-up of 13.6 (5.6-36.4) months, 5-year pancreas graft survival was 85% for the whole series, kidney graft survival was 90% for and patient survival was 94.7%. Conclusion(s): To date, this is the largest series describing the use of postmortem NRP in cDCDD pancreas transplantation, displaying competitive results in terms of graft/patient survival.
20th Biennial European Society for Organ Transplantation Congress, ESOT 2021. Milan Italy

Ferrer-Fabrega, J., et al. (2021). "Controlled donation after circulatory death pancreas transplantation in Spain. Initial experience." American Journal of Transplantation 21(SUPPL 4): 841.
Purpose: To report on the Spanish Pancreas Transplantation experience in controlled donation after circulatory death (cDCD) by analyzing the normothermic regional perfusion (NRP) and super-rapid recovery Methods: Data from January 2015 to December 2020 were analyzed regarding pancreas transplantations using cDCD donors Results: Some 471 pancreas transplants were performed, 20 being from cDCD donors. NRP was used in 18 pancreases. The median functional WIT was 10 (7-15.5) min. NRP was run for 113.5 (91.5-134.5) minutes. Pancreas cold ischemia was 412.5 (330-636.7) minutes. Surgical complications were present in 70% of cases.Two patients of the NRP group presented with primary pancreas graft non-function. After a median follow-up of 13.6 (5.6-36.4) months, the 1 and 5-year overall pancreas survival rate was 85% for the whole series, 83.3% for NRP group and 100% for SSR group.Overall 1 and 5-year patient survival was 94.7%, with no group differences Conclusion(s): To date, this is the largest series describing the use of postmortem NRP in cDCD pancreas transplantation, providing competitive results in terms of graft/patient survival.
American Transplant Congress, ATC 2021. Virtual

Richards, J., et al. (2021). "Comparable outcomes for circulatory death and brain-stem death pancreas transplantation irrespective of the use of normothermic regional perfusion." Transplant International 34(SUPPL 1): 28.
Background: Simultaneous pancreas and kidney transplantation is the optimum treatment for patients with type 1 diabetes and renal failure, providing survival benefit over deceased donor kidney transplant alone. Method(s): We performed a retrospective analysis of prospectively collected outcomes of the first 10 years of our Donation after Circulatory Death (DCD) pancreas transplant program, including DCD donors undergoing Normothermic Regional Perfusion (NRP). Result(s): 211 patients (139 donation after brainstem death (DBD), 72 DCD (59 conventional DCD and 13 NRP retrieval)) were included in the study. Patient survival at 1, 3, 5, and 10 years was 99.0%, 96.6%, 93.4% and 84.3%, respectively, with no significant difference in patient survival between those recipients receiving grafts from DBD or conventional DCD donors. Death-censored pancreas and kidney graft survival at 5 years was 83.9% and 93.2%, respectively, with no significant difference between DCD and DBD cohorts. For those receiving a DCD graft, patient survival, and pancreas and kidney transplant outcomes were comparable, irrespective of whether the organs were procured conventionally or following NRP. Conclusions In conclusion, utilisation of DCD pancreases is a safe approach to expanding the donor pool with equivalent results to DBD transplantation. Pancreas transplantation following NRP appears to be feasible, but warrants further study.
20th Biennial European Society for Organ Transplantation Congress, ESOT 2021. Milan Italy

Richards, J., et al. (2021). "The impact of normothermic regional perfusion on simultaneous kidney and

PRELIMINARY DRAFT

pancreas transplantation." Transplant International 34(SUPPL 1): 205.
Background: Simultaneous pancreas and kidney (SPK) transplantation is the optimum treatment for patients with type 1 diabetes and renal failure and provides survival benefit over deceased donor kidney transplantation alone. Donation after circulatory death (DCD) SPK transplantation has equivalent long-term results to organs from brainstem dead donors (DBD), but is associated with increased rates of ischemia reperfusion injury and delayed graft function. Normothermic Regional Perfusion (NRP) has emerged as a promising technique to minimise or reverse the additional ischemic insult associated with conventional DCD (sDCD) donation by placing the donor on a modified extra-corporeal membrane oxygenator circuit. To date, little has been published on the outcomes of pancreas transplantation following NRP beyond case reports. Method(s): We performed a retrospective analysis of prospectively collected outcomes our DCD pancreas transplant program and comparing the outcomes of recipients receiving SPK grafts following sDCD and NRP procurement. Result(s): 266 patients were included in the study (171 DBD, 77 sDCD, 18 NRP). There was no significant difference between cohorts in terms of serum creatinine, eGFR at 1 year or HbA1c. There were no significant differences in the potential biochemical markers of graft pancreatitis (CRP, White Blood Count, Neutrophil Count, Albumin, Platelet Count, Amylase, Lipase). There was a significantly lower rate of renal delayed graft function (DGF) in the DBD 41/171 (24.0%) and NRP cohorts 5/18 (27.8%) compared to sDCD cohort 42/77 (54.5%). No differences were seen in the rates of pancreas DGF in DBD 4/171 (2.3%), sDCD cohort 3/77 (3.9%) and NRP cohorts 0/18 (0%). Conclusion(s): While there is increasingly strong evidence showing benefit in the setting of liver transplantation, the benefit in the setting of pancreas transplantation is less clear. We believe this paper represents the largest single centre DCD and NRP series in the setting of SPK presented to date and in it demonstrated that.
20th Biennial European Society for Organ Transplantation Congress, ESOT 2021. Milan Italy

http://www.ont.es/infesp/Paginas/DocumentosdeConsenso.aspx
The initial dose of sodium heparin recommended to establish PRN is 300 IU/Kg in bolus IV, to which a dose of 1000 IU would be added for priming the system. Periodic re- heparinization of the system may be necessary.

PICO 2: For whole pancreas transplantation, is normothermic regional perfusion compatible with the procurement of other abdominal (kidneys, liver) organs?

RECOMMENDATION/STATEMENT: For whole pancreas transplantation, normothermic regional perfusion is compatible with the procurement of other abdominal (kidneys, liver) organs.

Quality of evidence: Moderate

Strength of recommendation: Strong for

Analysis of the literature:
All published studies describing the use of normothermic regional perfusion in the context of cDCD pancreas transplant reported recovery and utilization of the liver, kidney or both organs for transplantation.

Oniscu GC et al, 2022
Impact of NRP on Organ Utilization: Nine-thousand nine-hundred one adult DCD donors consented for donation in the period considered, of which 2587 (26%) were excluded because of prolonged time to asystole, and 12 NRP cases were removed, giving 7302 donors. Of these 7302 donors, 6440 had at least 1 organ (abdominal or cardiothoracic) offered for

PRELIMINARY DRAFT

transplantation and 4716 had at least 1 abdominal organ accepted for transplantation before retrieval and were considered in the subsequent analyses.
When applying organ-specific criteria, 4276 donors had the liver offered, 1906 donors had the pancreas offered, and 4622 donors had at least 1 kidney offered for transplantation before retrieval (9223 kidneys offered in total).

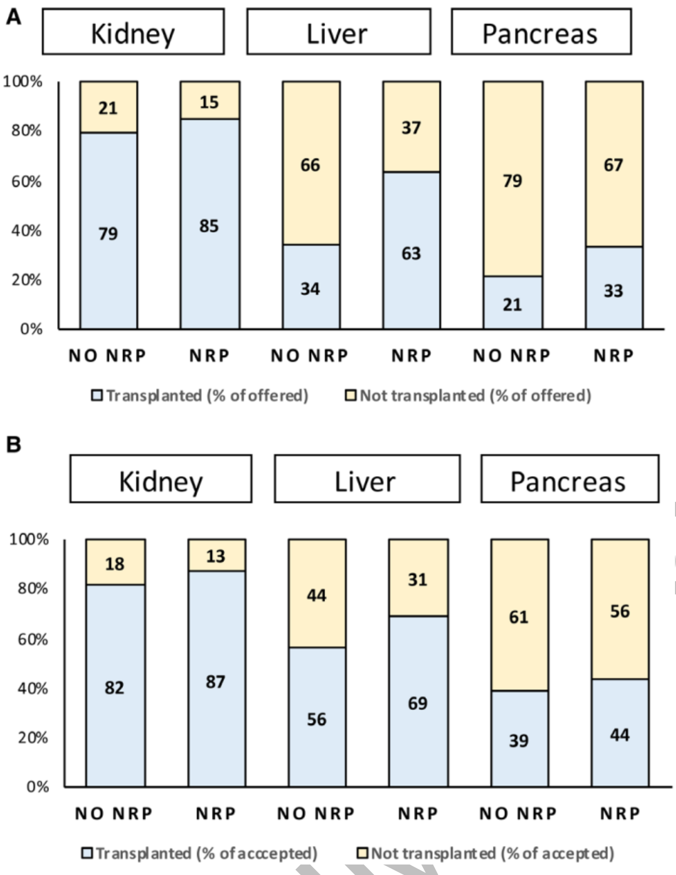

FIGURE. Individual organ utilization according to the use of NRP. A, Percentage of organs transplanted of those offered. B, Percentage of organs transplanted of those retrieved. NRP, normothermic regional perfusion.

The use of NRP (not adjusted for differences in donor risk profile) was associated with improved organ utilization rates for liver, kidney, and pancreas from the total number of organ offers as illustrated in Figure A. We also undertook a comparison of the number of organs accepted that were retrieved and transplanted (Figure B) and found a significant increase in transplantation rates for livers (P = 0.006, chi-square) and kidneys (P = 0.03, chi-square) if NRP was used.

Richards JA et al, 2021
A total of 13 simultaneous pancreas and kidney were performed. *No information regarding liver retrieval is present in the article.*
UK organ retrieval protocol: A rapid procurement technique via a midline laparotomy is utilised, with dual aortic and portal venous perfusion with University of Wisconsin solution (Belzer UW, Bridge to Life Ltd., Columbia, USA) containing 25,000 units of heparin per litre in the first two litres with additional topical cooling with crushed frozen saline. The liver-pancreas block is

PRELIMINARY DRAFT

then either split in situ or on the backtable according to the preference of the retrieving surgeon. Our DCD pancreas transplant programme started in August 2008.

Miñambres E et al, 2017
A total of 37 kidneys, 11 livers, six bilateral lungs, and one pancreas obtained from cDCD donors were finally transplanted.

Oniscu GC et al, 2014
Sixty-three organs were recovered achieving an organ recovery rate of three organs/donor compared to the national DCD average of 2.6 organs/donor. Forty-nine patients received a transplant. The abdominal organ transplant activity is detailed in Table 2.
Two combined pancreas– kidney transplants, one islet transplant and three double lung transplants were performed with primary function. Eleven livers were recovered and transplanted.

Table 2: Individual center normothermic regional perfusion retrieval and organ transplant activity

	Number of			
Transplant center	Donors	Livers	Kidneys	Pancreata
Birmingham	3	2	5[1]	–
Cambridge	9	4	16[2]	2
Edinburgh	9	5	17[3]	1[4]
All	21	11	38	3

[1]One donor had a previous nephrectomy.
[2]Three double kidney transplants, two discarded, two combined pancreas and kidney transplants.
[3]One double kidney transplant and one discarded.
[4]One pancreas used for research, one pancreas for islet isolation with insufficient yield and one pancreas used for islets.

Butler AJ et al, 2014
Eight controlled DCD donors underwent NRP from which 3 livers, 2 pancreases, and 14 kidneys were
transplanted. Four livers were not used because of biochemical evidence of hepatocellular damage and one because of cirrhosis. Two kidneys were lost from venous thrombosis before function returned and two developed delayed graft function; all transplanted livers and pancreases had primary function.

Magliocca JF et al, 2005
Magliocca et al published a single case within a multiorgan DCD series. A total of 24 kidney, 5 liver, and 1 pancreas transplants were performed with these organs.

Farney AC et al, 2008
From April 1, 2003, to October 3, 2007, 53 kidney transplantations and 4 simultaneous kidney-pancreas transplantations from DCD donors were performed. All DCD donor kidneys were managed with pulsatile perfusion preservation, and all simultaneous kidney-pancreas transplantation donors were managed with extracorporeal support.
No information regarding liver retrieval is present in the article.

Rojas-Peña A, et al, 2014
A retrospective review of all potential controlled-DCDD cases between October 1, 2000 and July 31, 2013 was performed.

PRELIMINARY DRAFT

Forty-eight renal grafts (65.8%), thirteen livers (61.9%), and one pancreas (50%) were successfully transplanted.

Mesnard B et al, 2022
Simultaneous pancreas-kidney transplantation was performed

References:

Oniscu GC, Mehew J, Butler AJ, Sutherland A, Gaurav R, Hogg R, Currie I, Jones M, Watson CJE. Improved Organ Utilization and Better Transplant Outcomes With In Situ Normothermic Regional Perfusion in Controlled Donation After Circulatory Death. Transplantation. 2022 Aug 22. doi: 10.1097/TP.0000000000004280. Epub ahead of print. PMID: 35993664.

Richards JA, Roberts JL, Fedotovs A, Paul S, Cottee S, Defries G, et al. Outcomes for circulatory death and brainstem death pancreas transplantation with or without use of normothermic regional perfusion. British Journal of Surgery. 2021;108(12):1406-8.

Minambres E, Suberviola B, Dominguez-Gil B, Rodrigo E, Ruiz-San Millan JC, Rodriguez-San Juan JC, et al. Improving the Outcomes of Organs Obtained From Controlled Donation After Circulatory Death Donors Using Abdominal Normothermic Regional Perfusion. American Journal of Transplantation. 2017;17(8):2165-72.

Oniscu GC, Randle LV, Muiesan P, Butler AJ, Currie IS, Perera MT, et al. In situ normothermic regional perfusion for controlled donation after circulatory death--the United Kingdom experience. American Journal of Transplantation. 2014;14(12):2846-54.

Butler AJ, Randle LV, Watson CJ. Normothermic regional perfusion for donation after circulatory death without prior heparinization. Transplantation. 2014;97(12):1272-8.

Magliocca JF, Magee JC, Rowe SA, Gravel MT, Chenault RH, 2nd, Merion RM, et al. Extracorporeal support for organ donation after cardiac death effectively expands the donor pool. J Trauma. 2005;58(6):1095-101; discussion 101-2.

Farney AC, Singh RP, Hines MH, Rogers J, Hartmann EL, Reeves-Daniel A, Gautreaux MD, Iskandar SS, Adams PL, Stratta RJ. Experience in renal and extrarenal transplantation with donation after cardiac death donors with selective use of extracorporeal support. J Am Coll Surg. 2008 May;206(5):1028-37; discussion 1037. doi: 10.1016/j.jamcollsurg.2007.12.029. Epub 2008 Mar 17. PMID: 18471749.

Rojas-Peña A, Sall LE, Gravel MT, et al. Donation after circulatory determination of death: the university of Michigan experience with extracorporeal support. Transplantation. 2014;98:328–334.

Mesnard B, Cantarovich D, Martin-Lefevre L, Rigaud J, Blancho G, Karam G, Badet L, Antoine C, Branchereau J. First French combined kidney/pancreas transplantation from controlled donation after circulatory arrest (Maastricht III). Prog Urol. 2022 Jan;32(1):1-2. doi: 10.1016/j.purol.2021.10.001. Epub 2021 Nov 10. PMID: 34772617.

PICO 3: For whole pancreas transplantation, is normothermic regional perfusion compatible with the procurement of other thoracic (heart, lungs) organs?

PRELIMINARY DRAFT

RECOMMENDATION/STATEMENT: For whole pancreas transplantation, normothermic regional perfusion is compatible with the procurement of other thoracic (heart, lungs) organs.

Quality of evidence: low – Expert opinion

Strength of recommendation: Strong for

Analysis of the literature:
Oniscu GC et al, 2022
Impact of NRP on Organ Utilization: Nine-thousand nine-hundred one adult DCD donors consented for donation in the period considered, of which 2587 (26%) were excluded because of prolonged time to asystole, and 12 NRP cases were removed as detailed above, giving 7302 donors. Of these 7302 donors, 6440 had at least 1 organ (abdominal or cardiothoracic) offered for transplantation and 4716 had at least 1 abdominal organ accepted for transplantation before retrieval and were considered in the subsequent analyses. Of the 4716 donors considered, there were 1862 donors who had at least 1 lung offered for transplantation. There were 379 donors who had their heart offered for transplantation, of which 42 underwent A-NRP (48% were transplanted) and 336 did not undergo NRP (23% were transplanted). The UK DCD Heart program began in February 2015 and was only in operation at certain transplant centers during the period considered. Although the utilization of the cardiothoracic organs was not a direct focus of this article, during this study, NRP has facilitated cDCD heart transplantation.

DRAFT

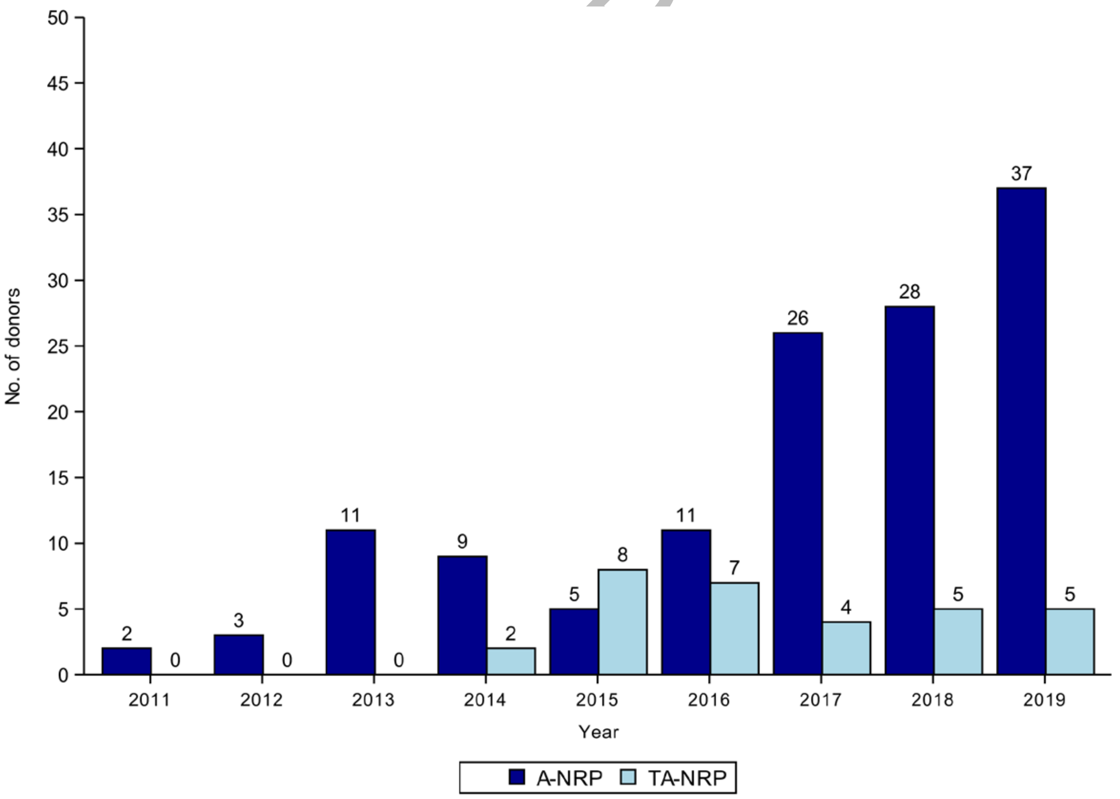

FIGURE. NRP use during the study period (2011–2019) by y and whether A-NRP or TA-NRP was used. A-NRP, abdominal normothermic regional perfusion; NRP, normothermic regional perfusion; TA-NRP, thoraco-abdominal normothermic regional perfusion.

Richards JA et al, 2021
Since April 2010, organ retrieval in the UK has been performed by a dedicated National Organ Retrieval Service (NORS), according to an agreed protocol: Thoracoabdominal NRP was performed via cannulae in the ascending aorta and IVC (either directly or via the right atrial appendage), or cannulating the abdominal aorta and IVC, with a cross-clamp placed across the origins of the brachiocephalic trunk, left common carotid and left subclavian arteries. The aortic arch is vented to ensure no cerebral flow. Given ante-mortem cannulation and heparinisation are not permitted in the UK, we add 50 000 units heparin to the NRP circuit.
No information is provided in relation to the retrieval of thoracic organs.

Miñambres E et al, 2017
A total of six double lungs were recovered and successfully transplanted. It is quite clear that abdominal
NRP combined with RR of the lungs is safe for both abdominal and thoracic grafts.
No information is provided in relation to the retrieval of the heart.

Oniscu GC et al, 2014
Two combined pancreas– kidney transplants, one islet transplant and three double lung transplants were performed with primary function.
No information is provided in relation to the retrieval of the heart.

Butler AJ et al, 2014
In one donor, both lungs were also removed after in situ cold perfusion and topical cooling; these were cold stored before transplantation and experienced primary function once transplanted, the recipient being extubated the following day.
No information is provided in relation to the retrieval of the heart.

Magliocca JF et al, 2005
No information is provided in relation to the retrieval of the heart.

Farney AC et al, 2008
No information is provided in relation to the retrieval of thoracic organs.

Rojas-Peña A, et al, 2014
No information is provided in relation to the retrieval of thoracic organs

Mesnard B et al, 2022
No information is provided in relation to the retrieval of thoracic organs

References:
Oniscu GC, Mehew J, Butler AJ, Sutherland A, Gaurav R, Hogg R, Currie I, Jones M, Watson CJE. Improved Organ Utilization and Better Transplant Outcomes With In Situ Normothermic Regional Perfusion in Controlled Donation After Circulatory Death. Transplantation. 2022 Aug 22. doi: 10.1097/TP.0000000000004280. Epub ahead of print. PMID: 35993664.

Richards JA, Roberts JL, Fedotovs A, Paul S, Cottee S, Defries G, et al. Outcomes for circulatory death and brainstem death pancreas transplantation with or without use of normothermic regional perfusion. British Journal of Surgery. 2021;108(12):1406-8.

PRELIMINARY DRAFT

Minambres E, Suberviola B, Dominguez-Gil B, Rodrigo E, Ruiz-San Millan JC, Rodriguez-San Juan JC, et al. Improving the Outcomes of Organs Obtained From Controlled Donation After Circulatory Death Donors Using Abdominal Normothermic Regional Perfusion. American Journal of Transplantation. 2017;17(8):2165-72.

Oniscu GC, Randle LV, Muiesan P, Butler AJ, Currie IS, Perera MT, et al. In situ normothermic regional perfusion for controlled donation after circulatory death--the United Kingdom experience. American Journal of Transplantation. 2014;14(12):2846-54.

Butler AJ, Randle LV, Watson CJ. Normothermic regional perfusion for donation after circulatory death without prior heparinization. Transplantation. 2014;97(12):1272-8.

Magliocca JF, Magee JC, Rowe SA, Gravel MT, Chenault RH, 2nd, Merion RM, et al. Extracorporeal support for organ donation after cardiac death effectively expands the donor pool. J Trauma. 2005;58(6):1095-101; discussion 101-2.

Farney AC, Singh RP, Hines MH, Rogers J, Hartmann EL, Reeves-Daniel A, Gautreaux MD, Iskandar SS, Adams PL, Stratta RJ. Experience in renal and extrarenal transplantation with donation after cardiac death donors with selective use of extracorporeal support. J Am Coll Surg. 2008 May;206(5):1028-37; discussion 1037. doi: 10.1016/j.jamcollsurg.2007.12.029. Epub 2008 Mar 17. PMID: 18471749.

Rojas-Peña A, Sall LE, Gravel MT, et al. Donation after circulatory determination of death: the university of Michigan experience with extracorporeal support. Transplantation. 2014;98:328–334.

Mesnard B, Cantarovich D, Martin-Lefevre L, Rigaud J, Blancho G, Karam G, Badet L, Antoine C, Branchereau J. First French combined kidney/pancreas transplantation from controlled donation after circulatory arrest (Maastricht III). Prog Urol. 2022 Jan;32(1):1-2. doi: 10.1016/j.purol.2021.10.001. Epub 2021 Nov 10. PMID: 34772617.

PICO 4: Should post-mortem normothermic regional perfusion be run for a maximum of 3 hours in the context of whole pancreas transplantation?

RECOMMENDATION/STATEMENT: In the context of whole pancreas transplantation normothermic regional perfusion should be maintained between 1 hour and 3 hours.

Quality of evidence: Low – Expert opinion
Expert opinion: Normothermic regional perfusion should be performed for more than 1h but less than 3 hours. Extended perfusion should be avoided to avoid graft edema.
The recommended time on CEC/ECMO would be 60 minutes, which can be extended to 120 minutes if necessary. It is considered that the dissection of the vascular structures and the preparation of the liver/pancreas should be carried out during this time, to avoid returning to normothermia once the extraction of the thoracic organs (lung and heart) has been carried out.

Strength of recommendation: Strong for

PRELIMINARY DRAFT

Analysis of the literature:

Oniscu GC et al, 2022

Abdominal-NRP was undertaken for 2h with postmortem cannulation of abdominal vena cava and aorta (or iliac vein and artery) with an endovascular or external clamp occluding the descending thoracic aorta. The perfusion was stopped after 30 to 60 min when thoraco-abdominal NRP (TA-NRP) was undertaken and the heart allowed support to the limited thoraco-abdominal circulation while its function was evaluated; otherwise, perfusion continued for 2h. NRP was considered to have failed when a circulation could not be established or maintained for at least 30min to allow the initial set of blood tests to be collected and analyzed.

Richards JA et al, 2021

Perfusion of the abdominal organs for 2 hours to allow viability testing of the liver with limited mobilisation of the abdominal organs prior to in situ perfusion with cold University of Wisconsin preservation solution. The liver-pancreas block is then either split in situ or on the backtable according to the preference of the retrieving surgeon.

Miñambres E et al, 2017

1 h of normothermic regional perfusion

The extracorporeal membranous oxygenation circuit used was a Maquet Rotaflow (Maquet, Rastatt, Germany). Normothermic regional perfusion monitoring: The aim of performing abdominal NRP was to maintain a pump flow of 2–2.4 L/min. A continuous pressure of 60–65 mm Hg in the femoral arterial cannula was maintained, and a temperature of 37°C was maintained; bicarbonate was always administered just after NRP had started, to maintain pH 7.35–7.45, and a hematocrit >25% was targeted.

Oniscu GC et al, 2014

2 h of normothermic regional perfusion

NRP duration 2 h (34 min–2 h 36 min)

Perfusion pressure:1.7 to 4L/min, Tº: 37ºC. Whole blood. Oxygen air mixture

Butler AJ et al, 2014

The median duration of NRP was 122 min (range 34-156).

Magliocca JF et al, 2005

The circuit sweep gas (100% oxygen) was set at 4 L/min and the flow rate adjusted to keep the PaCO2 between 30 –50 mm Hg. Sodium bicarbonate was infused to maintain pH >7.1, and heparin was infused as required to maintain the activated clotting time >500 seconds.

Concurrent with initiation of the in situ flush procedure, ECMO perfusion was discontinued, the arterial line was clamped distal to the oxygenator and proximal to the preservation solution perfusion line, and venous return was collected with suction on the field.

Time from cardiac death to organ procurement (hrs): 1.4 (range 0.5 1–2.1).

Farney AC et al, 2008

In DCD donor patients supported with extracorporeal perfusion, families consented to vascular cannulation of the femoral vessels and systemic heparinization before withdrawal of life support. After 5 minutes of asystole and declaration of cardiac death, donors were cooled to 22°C and perfused with oxygenated blood at 4 to 6 L/minute flow rates. After initiation of extracorporeal support, the donor was transported to the operating room, where multiple organ procurement was performed using standard techniques similar to those for DBD donors.

No data regarding duration of NRP

PRELIMINARY DRAFT

Rojas-Peña A, et al, 2014
The average extracorporeal support (ECS) duration was 86 +/-5 min, and blood flow was 47+/-5.3 mL/kg/min (3.5 L/min)
When rapid recovery (RR)-DCDD is instituted after the 5-min period of asystole, the organs are typically retrieved within 30 to 45 min.

Mesnard B et al, 2022
Perfusion duration: 3 h. Getinge. 37ºC. Whole blood

	**Belgium**	**France**	**Italy**	**The Netherlands**	**Norway**	**Spain**	**UK**
**Duration of NRP**	Maximum 2h	Minimum 1h Maximum 4h Preferred 2h	Minimum 1h Preferred 3-4h	Minimum 1h Maximum 2h	Preferred 2h	Minimum 1h Maximum 4h	Preferred 2h

Jochmans I, et al; ESOT Workstream 04 of the TLJ (Transplant Learning Journey) project. Consensus statement on normothermic regional perfusion in donation after circulatory death: Report from the European Society for Organ Transplantation's Transplant Learning Journey. Transpl Int. 2021 Nov;34(11):2019-2030. doi: 10.1111/tri.13951. PMID: 34145644.

References:

Oniscu GC, Mehew J, Butler AJ, Sutherland A, Gaurav R, Hogg R, Currie I, Jones M, Watson CJE. Improved Organ Utilization and Better Transplant Outcomes With In Situ Normothermic Regional Perfusion in Controlled Donation After Circulatory Death. Transplantation. 2022 Aug 22. doi: 10.1097/TP.0000000000004280. Epub ahead of print. PMID: 35993664.

Richards JA, Roberts JL, Fedotovs A, Paul S, Cottee S, Defries G, et al. Outcomes for circulatory death and brainstem death pancreas transplantation with or without use of normothermic regional perfusion. British Journal of Surgery. 2021;108(12):1406-8.

Minambres E, Suberviola B, Dominguez-Gil B, Rodrigo E, Ruiz-San Millan JC, Rodriguez-San Juan JC, et al. Improving the Outcomes of Organs Obtained From Controlled Donation After Circulatory Death Donors Using Abdominal Normothermic Regional Perfusion. American Journal of Transplantation. 2017;17(8):2165-72.

Oniscu GC, Randle LV, Muiesan P, Butler AJ, Currie IS, Perera MT, et al. In situ normothermic regional perfusion for controlled donation after circulatory death--the United Kingdom experience. American Journal of Transplantation. 2014;14(12):2846-54.

Butler AJ, Randle LV, Watson CJ. Normothermic regional perfusion for donation after circulatory death without prior heparinization. Transplantation. 2014;97(12):1272-8.

Magliocca JF, Magee JC, Rowe SA, Gravel MT, Chenault RH, 2nd, Merion RM, et al. Extracorporeal support for organ donation after cardiac death effectively expands the donor pool. J Trauma. 2005;58(6):1095-101; discussion 101-2.

Farney AC, Singh RP, Hines MH, Rogers J, Hartmann EL, Reeves-Daniel A, Gautreaux MD, Iskandar SS, Adams PL, Stratta RJ. Experience in renal and extrarenal transplantation with donation after cardiac death donors with selective use of extracorporeal support. J Am Coll

PRELIMINARY DRAFT

Surg. 2008 May;206(5):1028-37; discussion 1037. doi: 10.1016/j.jamcollsurg.2007.12.029. Epub 2008 Mar 17. PMID: 18471749.

Rojas-Peña A, Sall LE, Gravel MT, et al. Donation after circulatory determination of death: the university of Michigan experience with extracorporeal support. Transplantation. 2014;98:328–334.

Mesnard B, Cantarovich D, Martin-Lefevre L, Rigaud J, Blancho G, Karam G, Badet L, Antoine C, Branchereau J. First French combined kidney/pancreas transplantation from controlled donation after circulatory arrest (Maastricht III). Prog Urol. 2022 Jan;32(1):1-2. doi: 10.1016/j.purol.2021.10.001. Epub 2021 Nov 10. PMID: 34772617.

Jochmans I, Hessheimer AJ, Neyrinck AP, Paredes D, Bellini MI, Dark JH, Kimenai HJAN, Pengel LHM, Watson CJE; ESOT Workstream 04 of the TLJ (Transplant Learning Journey) project. Consensus statement on normothermic regional perfusion in donation after circulatory death: Report from the European Society for Organ Transplantation's Transplant Learning Journey. Transpl Int. 2021 Nov;34(11):2019-2030. doi: 10.1111/tri.13951. PMID: 34145644.

PICO 5: Should valid parameters (machine perfusion -monitoring flow and temperature-, analytical/biochemical parameters and functional warm ischemia time) be defined to assess the quality of the pancreatic graft before deciding the suitability/validity of the organ for whole pancreas transplant?

RECOMMENDATION/STATEMENT: Valid parameters (machine perfusion -monitoring flow and temperature-, analytical/biochemical parameters and functional warm ischemia time) should be defined to assess the quality of the pancreatic graft before deciding the suitability/validity of the organ for whole pancreas transplant.

Donation parameters:

- Total warm ischemia time < 60 minutes. (From withdrawal of ventilatory support to start of preservation).
- Functional or true warm ischemia time < 30 minutes. (From systolic blood pressure < 60 mmHg to start of preservation)
- Hemodynamic instability (SBP < 60mmHg) prior to WLST < 60 minutes.
- Preferable cold ischemia time < 8 hours.
WLST, withdraw life-sustaining therapies

Functional parameters

- Renal biochemistry prior to WLST (Cr < 2.0 mg/dl), after diagnosis of death (Cr < 2.5 mg/dl) and evolution curve during PAN every 30 minutes with a final Cr < 2.5 mg/dl.
- Initial liver biochemistry (before LLST and diagnosis of death): AST, ALT < 3 times the normal value.
- Evolutionary and final liver biochemistry: AST, ALT < 4 times the normal value.
- Initial and final pancreatic biochemistry (amylase and/or lipase): < 3 times the normal value.

Quality of evidence: Low – Expert opinion

Strength of recommendation: Strong for

PRELIMINARY DRAFT

Analysis of the literature:

Oniscu GC et al, 2022
- Median TWIT (min) (IQR): 31 (26–41).
- Median FWIT (min) (IQR): 23 (18–28).

TWIT is defined as the time from treatment withdrawal to the start of NRP/start of in situ cold perfusion for non-NRP group (missing data: 613 [13%] for non NRP group and 3 [2%] for NRP group).
FWIT is defined as the time from systolic BP reaching 50 mm Hg to the start of NRP/start of in situ cold perfusion for non-NRP group (missing data: 1435 [32%] for non-NRP and 46 [28%] for NRP group).
No functional parameters data is present.
A-NRP was undertaken for 2 h with postmortem cannulation of abdominal vena cava and aorta (or iliac vein and artery) with an endovascular or external clamp occluding the descending thoracic aorta. The perfusion was stopped after 30 to 60 min when thoraco-abdominal NRP (TA-NRP) was undertaken and the heart allowed support to the limited thoraco-abdominal circulation while its function was evaluated; otherwise, perfusion continued for 2 h. NRP was considered to have failed when a circulation could not be established or maintained for at least 30 min to allow the initial set of blood tests to be collected and analyzed.

Richards JA et al ,2021
- Time from WLST (or Cross-clamp in DBD) to cold perfusion or NRP (as appropriate) (minutes): 38 (16-185)
- Time from Arrest (or Cross-Clamp in DBD) to start of cold perfusion or NRP (as appropriate) (minutes): 13 (10-18)
- Pancreas Cold Ischemia Time (minutes): 517 (361-692)

UK organ retrieval teams wait a minimum of three hours from WLST before abandoning retrieval; they have previously demonstrated that it is possible to pursue donation for longer. In the UK, intravenous administration of heparin or other pre-mortem interventions aimed specifically at facilitating organ donation are not permissible.
They perfuse the abdominal organs for 2 hours to allow viability testing of the liver with limited mobilization of the abdominal organs prior to in situ perfusion with cold University of Wisconsin preservation solution. The liver-pancreas block is then either split in situ or on the backtable according to the preference of the retrieving surgeon.

Miñambres E et al, 2017
Blood samples from the ECMO device were obtained just after starting nRP and at least every 30 min for biochemistry analysis, serum lactate levels, and hematocrit values. If alanine transaminase (ALT) or aspartate transaminase (AST) levels at 30 or 60 min after nRP were >4 times the normal values, the liver was discarded even if it had a normal macroscopic appearance.
An extended withdrawal period is detrimental to the viability of grafts and prolonged WIT is a strong indicator of poor graft survival (4,5,37). Therefore, there is no clear consensus on the magnitude of the effect of WIT on graft viability. However, in current practice, FWIT of >30 min is applied as a contraindication for liver and pancreas and >60 min for kidneys and lungs in most centers, but the strict upper limit of FWIT remains controversial. The use of premortem interventions before nRP decreases the FWIT and may increase the number and quality of grafts recovered in cDCD. Some authors have proposed increasing donor time from WTLS to death up to 4 h with no negative impact in kidney recipients. We believe it might also be increased for livers if macroscopic views and biochemical marker analyses during abdominal NRP are not altered.

PRELIMINARY DRAFT

Oniscu GC et al, 2014
Two simultaneous pancreas–kidney transplants were performed in type I diabetic patients aged 52 and 34 years old, with primary renal and pancreatic function. The CITs for the pancreata were 8 h 52 min and 7 h 32 min, respectively.
- Withdrawal to asystole: 13 min (6 min–249 min)
- Asystole to NRP: 16 min (10 min–23 min)
- Functional warm ischemia time: 26 min (13 min–48 min)
- NRP duration: 2 h (34 min–2 h 36 min)

Abdominal organ function and homeostasis during NRP were monitored every 30 min using blood gases (pH, pO2, pCO2, bicarbonate, base excess, lactate), and every 30–60 min for hematology (hemoglobin, hematocrit, white cell count, platelet count, activated partial thromboplastin time) and biochemistry (alanine transaminase [ALT], bilirubin, alkaline phosphatase, urea, creatinine, glucose, sodium and potassium).
The current criteria for DCD selection were observed, with a functional warm ischemia time (WIT; from systolic BP<50mmHg to start of perfusion) of less than 30 min for the liver and pancreas, and 1 h for kidneys. In addition, as suggested by the Spanish experience in uncontrolled DCD liver donation, an ALT <3 times the upper limit of normal at the initiation of NRP and <4 times the upper limit of normal at the end of NRP were considered when selecting the liver grafts (7). There were no additional donor exclusion criteria and we attempted NRP in all donors that the teams could attend. Organs were allocated in keeping with the current United Kingdom allocation criteria

Butler AJ et al, 2014
- The median time from withdrawal of treatment to circulatory arrest of the donors was 12 min (range 6-249), the median time from asystole to the onset of warm perfusion was 15 min (range 10-20), and the median duration of NRP was 122 min (range 34-156). During the period of NRP, flow rates of 2 to 4 L/min were achieved, with mean pressures between 30 and 50 mm Hg measured with a cannula in the left external iliac artery. When the flow rate fell, it was either a result of manipulation of the liver or volume depletion, with the venous cannula often becoming occluded against the atrial wall; addition of fluid effectively restored flows. In most cases, the supplementary fluid was Gelofusine, but four cases required additional packed red cells because of bleeding and a low hematocrit.
- What threshold value for ALT or AST would be appropriate is not clear.

Magliocca JF et al, 2005
Time to cardiac death after withdrawal of support initiated (min): 17± 15 (2–50)
Time from cardiac death to organ procurement (hrs): 1.4 ± 0.5 (1–2.1)

Farney AC et al, 2008
Donor evaluation and management: The donor consent process and medical management were independently carried out by the donor organ procurement organization. Withdrawal of life support most commonly occurred in the intensive care unit setting with the family present. Vital signs (blood pressure, heart rate and rhythm, respiratory rate, and transcutaneous oxygen saturation) were monitored after withdrawal of life support until asystole occurred. Cardiac death was declared after 5 minutes of asystole, and the donor was sent to the operating room
for rapid organ procurement. The technique most commonly used for DCD donors in this study was open laparotomy with direct vascular cannulation and in situ perfusion with Viaspan (Bard Laboratories) preservation solution combined with topical cooling.
No specific parameters are provided

Rojas-Peña A, et al, 2014

PRELIMINARY DRAFT

The average extracorporeal support (ECS) duration was 86±5 min, and blood flow was 47±5.3 mL/kg/min (3.5 L/min). ECS restored the metabolic and respiratory acidosis reflected in improved oxygen delivery and saturation until organ procurement.

Mesnard B et al, 2022
Perfusion duration: 3 h. Getinge. 37ºC. Whole blood

	Belgium	France	Italy	The Netherlands	Norway	Spain	UK	TLJ 2.0 WS04 states:
**DCD pancreas evaluation with NRP**		Ischemia : fWIT <30 min; NRP >1 to 4 hours; Macroscopic aspect		Macroscopic aspect		Perfusate: AST/ALT stable and <4x ULN; Ischemia: fWIT <30 min; Macroscopic aspect	Macroscopic aspect	Pancreata may be assessed based on the evolution of biochemical markers in the perfusate and their macroscopic aspect.

Jochmans I, et al; ESOT Workstream 04 of the TLJ (Transplant Learning Journey) project. Consensus statement on normothermic regional perfusion in donation after circulatory death: Report from the European Society for Organ Transplantation's Transplant Learning Journey. Transpl Int. 2021 Nov;34(11):2019-2030. doi: 10.1111/tri.13951. PMID: 34145644.

References:

Oniscu GC, Mehew J, Butler AJ, Sutherland A, Gaurav R, Hogg R, Currie I, Jones M, Watson CJE. Improved Organ Utilization and Better Transplant Outcomes With In Situ Normothermic Regional Perfusion in Controlled Donation After Circulatory Death. Transplantation. 2022 Aug 22. doi: 10.1097/TP.0000000000004280. Epub ahead of print. PMID: 35993664.

Richards JA, Roberts JL, Fedotovs A, Paul S, Cottee S, Defries G, et al. Outcomes for circulatory death and brainstem death pancreas transplantation with or without use of normothermic regional perfusion. British Journal of Surgery. 2021;108(12):1406-8.

Minambres E, Suberviola B, Dominguez-Gil B, Rodrigo E, Ruiz-San Millan JC, Rodriguez-San Juan JC, et al. Improving the Outcomes of Organs Obtained From Controlled Donation After Circulatory Death Donors Using Abdominal Normothermic Regional Perfusion. American Journal of Transplantation. 2017;17(8):2165-72.

Oniscu GC, Randle LV, Muiesan P, Butler AJ, Currie IS, Perera MT, et al. In situ normothermic regional perfusion for controlled donation after circulatory death--the United Kingdom experience. American Journal of Transplantation. 2014;14(12):2846-54.

Butler AJ, Randle LV, Watson CJ. Normothermic regional perfusion for donation after circulatory death without prior heparinization. Transplantation. 2014;97(12):1272-8.

PRELIMINARY DRAFT

Magliocca JF, Magee JC, Rowe SA, Gravel MT, Chenault RH, 2nd, Merion RM, et al. Extracorporeal support for organ donation after cardiac death effectively expands the donor pool. J Trauma. 2005;58(6):1095-101; discussion 101-2.

Farney AC, Singh RP, Hines MH, Rogers J, Hartmann EL, Reeves-Daniel A, Gautreaux MD, Iskandar SS, Adams PL, Stratta RJ. Experience in renal and extrarenal transplantation with donation after cardiac death donors with selective use of extracorporeal support. J Am Coll Surg. 2008 May;206(5):1028-37; discussion 1037. doi: 10.1016/j.jamcollsurg.2007.12.029. Epub 2008 Mar 17. PMID: 18471749.

Rojas-Peña A, Sall LE, Gravel MT, et al. Donation after circulatory determination of death: the university of Michigan experience with extracorporeal support. Transplantation. 2014;98:328–334.

Mesnard B, Cantarovich D, Martin-Lefevre L, Rigaud J, Blancho G, Karam G, Badet L, Antoine C, Branchereau J. First French combined kidney/pancreas transplantation from controlled donation after circulatory arrest (Maastricht III). Prog Urol. 2022 Jan;32(1):1-2. doi: 10.1016/j.purol.2021.10.001. Epub 2021 Nov 10. PMID: 34772617.

Jochmans I, Hessheimer AJ, Neyrinck AP, Paredes D, Bellini MI, Dark JH, Kimenai HJAN, Pengel LHM, Watson CJE; ESOT Workstream 04 of the TLJ (Transplant Learning Journey) project. Consensus statement on normothermic regional perfusion in donation after circulatory death: Report from the European Society for Organ Transplantation's Transplant Learning Journey. Transpl Int. 2021 Nov;34(11):2019-2030. doi: 10.1111/tri.13951. PMID: 34145644.

PICO 6: Could normothermic regional perfusion in donation after circulatory death improve graft and patient outcomes compared with in situ cooling and rapid procurement in pancreas transplantation?

RECOMMENDATION/STATEMENT: For whole pancreas transplantation normothermic regional perfusion in donation after circulatory death (DCD) might improve the graft and patient outcomes compare with in situ cooling and rapid procurement.

Quality of evidence: Low – Expert opinion

Strength of recommendation: Strong for

Analysis of the literature:
Nine primary studies reporting on some pancreas transplants after NRP were identified. Not all groups used DCD with in situ cooling and rapid procurement to be compared.
Other series using NRP and rapid technique in cDCD pancreas transplantation were presented as an abstract*.

Oniscu GC et al, 2022
Of the 331 first SPK transplants undertaken, 28 (7%) used cDCD NRP grafts; 4% of the NRP transplants failed within 12 mo, compared with 10% in the non-NRP group. There was no significant difference between the groups (P = 0.2889) in terms of risk-adjusted 12-mo graft failure. There was no impact of the functional warm ischemia time in the overall model with no significant difference in graft survival for each min increase (P = 0.826).
Conclusions: The use of NRP during DCD organ recovery leads to increased organ utilization and improved transplant outcomes compared with conventional organ recovery.

PRELIMINARY DRAFT

Richards JA et al, 2021
A total of 211 patients (139 DBD and 72 DCD, of which 59 were conventional DCD (sDCD) and 13 normothermic regional perfusion were included.
In this series of DCD simultaneous pancreas and kidney transplantation, long-term follow-up data demonstrate that patient and graft survival are equivalent for sDCD and DBD organs with no difference in graft function at 1 year. The findings indicate that pancreas transplantation following normothermic regional perfusion is both feasible and offers comparable outcomes. Lower levels of both amylase and lipase were seen in recipients of grafts from DBD compared with sDCD donors. Serum lipase, but not amylase, levels were also significantly lower in the normothermic regional perfusion cohort compared with sDCD, which may suggest less severe graft pancreatitis.
There was no significant difference in patient or graft survival between sDCD or normothermic regional perfusion donors, nor in the rates of primary non-function, DGF, thrombosis, episodes of acute rejection, reoperation or readmission between sDCD or normothermic regional perfusion cohorts. There was no significant difference between sDCD and normothermic regional perfusion cohorts in terms of serum creatinine, eGFR or HbA1c at 1 year.

Miñambres E et al, 2017
Simultaneous kidney–pancreas transplantation was performed using NRP with appropriate graft function after 6-month follow-up.

Oniscu GC et al, 2014
DCD data with in situ cooling and rapid procurement were not provided

Butler AJ et al, 2014
DCD data with in situ cooling and rapid procurement were not provided

Magliocca JF et al, 2005
DCD data with in situ cooling and rapid procurement were not provided

Farney AC et al, 2008
Patient- and graft-survival rates were 100% in the 4 simultaneous kidney-pancreas transplantations.
Pancreas grafts were used for transplantation only if extracorporeal support was used during procurement. Kidney and pancreas grafts were procured from four DCD donors and transplanted into 4 recipients. All 4 simultaneous kidneypancreas transplantation recipients experienced immediate graft function (no dialysis, no insulin) and continue to have excellent dual graft function, with a mean followup of 24 months.
DCD data with in situ cooling and rapid procurement were not provided

Rojas-Peña A, et al, 2014
Two pancreatic grafts were recovered, and one was transplanted.
DCD data with in situ cooling and rapid procurement were not provided

Mesnard B et al, 2022
Splenic thrombosis
DCD data with in situ cooling and rapid procurement were not provided

***ABSTRACT**
Ferrer, J., et al. (2021). "Pancreas transplantation from donors declared death by circulatory criteria: Initial experience in Spain." Transplant International 34(SUPPL 1): 173-174.

PRELIMINARY DRAFT

Data from the Spanish National Transplant Organization database and from transplant centers were retrospectively analyzed (2015- 2020). Result(s): During the study period, 471 pancreas transplants were performed, including 20 combined kidney-pancreas transplants from cDCDD donors. Of these, NRP was used in 18 procedures, all with ante-mortem cannulation, and rapid recovery (RR) in 2 cases. The median donor age was 33 years, 65% were male. The median total warm ischemia time (WIT) and the functional WIT were 19 (13.2-23.7) min. and 10 (7-15.5) min., respectively. Postmortem NRP was run for a 113.5 (91.5-134.5) min. The median pancreas cold ischemia was 412.5 (330- 636.7) min. The pancreatic graft function was optimal in all cases except for three (NRP cases), for which the cause was primary nonfunction in two (one of them requiring transplantectomy of pancreatic and kidney grafts) and cardiogenic and septic shock secondary to pancreatic fistula for a fatal case. Seven patients presented with delayed kidney graft function, with five cases requiring dialysis. Pancreas related surgical complications were present in 70% cases, haemorrhage being the most common. After a median follow-up of 13.6 (5.6-36.4) months, 5-year pancreas graft survival was 85% for the whole series, kidney graft survival was 90% for and patient survival was 94.7%. Conclusion(s): To date, this is the largest series describing the use of postmortem NRP in cDCDD pancreas transplantation, displaying competitive results in terms of graft/patient survival.

20th Biennial European Society for Organ Transplantation Congress, ESOT 2021. Milan Italy

Ferrer-Fabrega, J., et al. (2021). "Controlled donation after circulatory death pancreas transplantation in Spain. Initial experience." American Journal of Transplantation 21(SUPPL 4): 841.

Purpose: To report on the Spanish Pancreas Transplantation experience in controlled donation after circulatory death (cDCD) by analyzing the normothermic regional perfusion (NRP) and super-rapid recovery Methods: Data from January 2015 to December 2020 were analyzed regarding pancreas transplantations using cDCD donors Results: Some 471 pancreas transplants were performed, 20 being from cDCD donors. NRP was used in 18 pancreases. The median functional WIT was 10 (7-15.5) min. NRP was run for 113.5 (91.5-134.5) minutes. Pancreas cold ischemia was 412.5 (330-636.7) minutes. Surgical complications were present in 70% of cases. Two patients of the NRP group presented with primary pancreas graft non-function. After a median follow-up of 13.6 (5.6-36.4) months, the 1 and 5-year overall pancreas survival rate was 85% for the whole series, 83.3% for NRP group and 100% for SSR group.Overall 1 and 5-year patient survival was 94.7%, with no group differences Conclusion(s): To date, this is the largest series describing the use of postmortem NRP in cDCD pancreas transplantation, providing competitive results in terms of graft/patient survival.

American Transplant Congress, ATC 2021. Virtual

Richards, J., et al. (2021). "Comparable outcomes for circulatory death and brain-stem death pancreas transplantation irrespective of the use of normothermic regional perfusion." Transplant International 34(SUPPL 1): 28.

Background: Simultaneous pancreas and kidney transplantation is the optimum treatment for patients with type 1 diabetes and renal failure, providing survival benefit over deceased donor kidney transplant alone. Method(s): We performed a retrospective analysis of prospectively collected outcomes of the first 10 years of our Donation after Circulatory Death (DCD) pancreas transplant program, including DCD donors undergoing Normothermic Regional Perfusion (NRP). Result(s): 211 patients (139 donation after brainstem death (DBD), 72 DCD (59 conventional DCD and 13 NRP retrieval)) were included in the study. Patient survival at 1, 3, 5, and 10 years was 99.0%, 96.6%, 93.4% and 84.3%, respectively, with no significant difference in patient survival between those recipients receiving grafts from DBD or conventional DCD donors. Death-censored pancreas and kidney graft survival at 5 years was 83.9% and 93.2%, respectively, with no significant difference between DCD and DBD cohorts. For those receiving a DCD graft, patient survival, and pancreas and kidney transplant outcomes were comparable, irrespective of whether the organs were procured conventionally or following NRP. Conclusions In conclusion, utilisation of DCD pancreases is a safe approach to expanding the donor pool with equivalent results to DBD transplantation. Pancreas transplantation following NRP appears to be feasible, but warrants further study.

20th Biennial European Society for Organ Transplantation Congress, ESOT 2021. Milan Italy

PRELIMINARY DRAFT

Richards, J., et al. (2021). "The impact of normothermic regional perfusion on simultaneous kidney and pancreas transplantation." Transplant International 34(SUPPL 1): 205.

Background: Simultaneous pancreas and kidney (SPK) transplantation is the optimum treatment for patients with type 1 diabetes and renal failure and provides survival benefit over deceased donor kidney transplantation alone. Donation after circulatory death (DCD) SPK transplantation has equivalent long-term results to organs from brainstem dead donors (DBD), but is associated with increased rates of ischemia reperfusion injury and delayed graft function. Normothermic Regional Perfusion (NRP) has emerged as a promising technique to minimise or reverse the additional ischemic insult associated with conventional DCD (sDCD) donation by placing the donor on a modified extra-corporeal membrane oxygenator circuit. To date, little has been published on the outcomes of pancreas transplantation following NRP beyond case reports. Method(s): We performed a retrospective analysis of prospectively collected outcomes our DCD pancreas transplant program and comparing the outcomes of recipients receiving SPK grafts following sDCD and NRP procurement. Result(s): 266 patients were included in the study (171 DBD, 77 sDCD, 18 NRP). There was no significant difference between cohorts in terms of serum creatinine, eGFR at 1 year or HbA1c. There were no significant differences in the potential biochemical markers of graft pancreatitis (CRP, White Blood Count, Neutrophil Count, Albumin, Platelet Count, Amylase, Lipase). There was a significantly lower rate of renal delayed graft function (DGF) in the DBD 41/171 (24.0%) and NRP cohorts 5/18 (27.8%) compared to sDCD cohort 42/77 (54.5%). No differences were seen in the rates of pancreas DGF in DBD 4/171 (2.3%), sDCD cohort 3/77 (3.9%) and NRP cohorts 0/18 (0%). Conclusion(s): While there is increasingly strong evidence showing benefit in the setting of liver transplantation, the benefit in the setting of pancreas transplantation is less clear. We believe this paper represents the largest single centre DCD and NRP series in the setting of SPK presented to date and in it demonstrated that.

20th Biennial European Society for Organ Transplantation Congress, ESOT 2021. Milan Italy

References:

Oniscu GC, Mehew J, Butler AJ, Sutherland A, Gaurav R, Hogg R, Currie I, Jones M, Watson CJE. Improved Organ Utilization and Better Transplant Outcomes With In Situ Normothermic Regional Perfusion in Controlled Donation After Circulatory Death. Transplantation. 2022 Aug 22. doi: 10.1097/TP.0000000000004280. Epub ahead of print. PMID: 35993664.

Richards JA, Roberts JL, Fedotovs A, Paul S, Cottee S, Defries G, et al. Outcomes for circulatory death and brainstem death pancreas transplantation with or without use of normothermic regional perfusion. British Journal of Surgery. 2021;108(12):1406-8.

Minambres E, Suberviola B, Dominguez-Gil B, Rodrigo E, Ruiz-San Millan JC, Rodriguez-San Juan JC, et al. Improving the Outcomes of Organs Obtained From Controlled Donation After Circulatory Death Donors Using Abdominal Normothermic Regional Perfusion. American Journal of Transplantation. 2017;17(8):2165-72.

Oniscu GC, Randle LV, Muiesan P, Butler AJ, Currie IS, Perera MT, et al. In situ normothermic regional perfusion for controlled donation after circulatory death--the United Kingdom experience. American Journal of Transplantation. 2014;14(12):2846-54.

Butler AJ, Randle LV, Watson CJ. Normothermic regional perfusion for donation after circulatory death without prior heparinization. Transplantation. 2014;97(12):1272-8.

Magliocca JF, Magee JC, Rowe SA, Gravel MT, Chenault RH, 2nd, Merion RM, et al. Extracorporeal support for organ donation after cardiac death effectively expands the donor pool. J Trauma. 2005;58(6):1095-101; discussion 101-2.

PRELIMINARY DRAFT

Farney AC, Singh RP, Hines MH, Rogers J, Hartmann EL, Reeves-Daniel A, Gautreaux MD, Iskandar SS, Adams PL, Stratta RJ. Experience in renal and extrarenal transplantation with donation after cardiac death donors with selective use of extracorporeal support. J Am Coll Surg. 2008 May;206(5):1028-37; discussion 1037. doi: 10.1016/j.jamcollsurg.2007.12.029. Epub 2008 Mar 17. PMID: 18471749.

Rojas-Peña A, Sall LE, Gravel MT, et al. Donation after circulatory determination of death: the university of Michigan experience with extracorporeal support. Transplantation. 2014;98:328–334.

Mesnard B, Cantarovich D, Martin-Lefevre L, Rigaud J, Blancho G, Karam G, Badet L, Antoine C, Branchereau J. First French combined kidney/pancreas transplantation from controlled donation after circulatory arrest (Maastricht III). Prog Urol. 2022 Jan;32(1):1-2. doi: 10.1016/j.purol.2021.10.001. Epub 2021 Nov 10. PMID: 34772617.

PICO 7: Does the normothermic regional perfusion have the potential to expand the donor pool for whole pancreas transplantation?

RECOMMENDATION/STATEMENT: Normothermic regional perfusion has the potential to expand the donor pool for whole pancreas transplantation.

Quality of evidence: Low – Expert opinion

Strength of recommendation: Strong for

Analysis of the literature:
Oniscu GC et al, 2022
NRP is an independent factor in increasing organ utilization for all abdominal organs.
This is a retrospective analysis of UK adult cDCD donors' where at least 1 abdominal organ was accepted for transplantation between January 1, 2011, and December 31, 2019.
Results: A mean of 3.3 organs was transplanted when NRP was used compared with 2.6 organs per donor when NRP was not used. When adjusting for organ-specific donor risk profiles, the use of NRP increased the odds of all abdominal organs being transplanted by 3-fold for liver (P < 0.0001; 95% confidence interval [CI], 2.20-4.29), 1.5-fold for kidney (P = 0.12; 95% CI, 0.87-2.58), and 1.6-fold for pancreas (P = 0.0611; 95% CI, 0.98-2.64). Twelve-mo liver transplant survival was superior for recipients of a cDCD NRP graft with a 51% lower risk-adjusted hazard of transplant failure (HR = 0.494). In risk-adjusted analyses, NRP kidneys had a 35% lower chance of developing delayed graft function than non-NRP kidneys (odds ratio, 0.65; 95% CI, 0.465-0.901)' and the expected 12-mo estimated glomerular filtration rate was 6.3 mL/min/1.73 m2 better if abdominal NRP was used (P < 0.0001).
The use of NRP (not adjusted for differences in donor risk profile) was associated with improved organ utilization rates for liver, kidney, and pancreas from the total number of organ offers. They found a significant increase in transplantation rates for livers (P = 0.006, chi-square) and kidneys (P = 0.03, chi-square) if NRP was used.
Utilization rates, unadjusted for donor risk profile, were similar for donors who underwent A-NRP or TA-NRP, despite the TA-NRP donors being significantly younger (median age 40 y old versus 54 y old for A-NRP donors), but the numbers are rather small to draw definitive conclusions.

Richards JA et al, 2021

PRELIMINARY DRAFT

In this series of DCD simultaneous pancreas and kidney transplantation, long-term follow-up data demonstrate that patient and graft survival are equivalent for conventional DCD (sDCD) and DBD organs with no difference in graft function at 1 year.
Utilization of DCD pancreases is a safe approach to expanding the donor pool with equivalent results to DBD transplantation. Also, pancreas transplantation after normothermic regional perfusion is feasible, but requires on-going prospective study to ensure that the benefits seen for liver transplantation do not come at the expense of pancreas transplant outcomes.

Miñambres E et al, 2017
Simultaneous kidney–pancreas transplantation was performed with appropriate graft function after 6-month follow-up. There are portable ECMO devices that could be used for recovering organs in smaller centers that have no device available. The ECMO device should be used by experienced personnel. In our case, the system is managed by perfusionist nurses who are trained to manage ECMO devices, under the supervision of the donor coordinator.

Oniscu GC et al, 2014
The results of this study are encouraging on many accounts such as organ function and recovery rate. While NRP will not change organ allocation it may lead to a wider utilization and sharing of organs currently discarded or indeed lead to more liberal acceptance criteria

Butler AJ et al, 2014
Sixteen potential DCD donors were attended of whom nine died within 4 hr of treatment withdrawal, and one at just over 4 hr. Eight of the 10 DCD donors were treated with normothermic regional perfusion. NRP was attempted in the other two but abandoned. Our first attempt at NRP was abandoned because the circuit thrombosed due to clot forming on the oxygenator. After this, a shunt was introduced to the circuit to allow blood to bypass the oxygenator and heat exchanger for 2min to allow fullmixing with heparin. In the other failed attempt, cardiac cannulation was abandoned when the cannula was lodged in the coronary sinus, and the right atrium was perforated during attempts to dislodge it.
Initially, cannulation was in the ascending aorta and right atrium, but after our initial experience we adopted an abdominal approach, cannulating the right common iliac artery and the inferior vena cava (IVC) or the right common iliac vein. In both failed attempts at NRP, cold in situ perfusion of the donor organs was rapidly performed and kidneys used for transplantation; in neither case was the liver or pancreas usable for transplantation, but not for reasons related to the failed NRP attempt.

Magliocca JF et al, 2005
The implementation of a DCD protocol using extracorporeal perfusion increased the potential organ donor pool at our institution by 33%. This was accomplished without short term adverse effect on organ function compared with kidneys transplanted from DBD donors.
A total of 30 kidneys, seven livers, and one pancreas were procured from patients who would not have otherwise met criteria for declaration of brain death and subsequent donation. The DCD protocol resulted in a 24% increase in the overall number of deceased donor kidneys transplanted from donors at our institution.

Farney AC et al, 2008
Pancreas grafts were used for transplantation only if extracorporeal support was used during procurement. Kidney and pancreas grafts were procured from four DCD donors and transplanted into 4 recipients. All 4 simultaneous kidney pancreas transplantation recipients experienced immediate graft function (no dialysis, no insulin) and continue to have excellent dual graft function, with a mean follow up of 24 months.

PRELIMINARY DRAFT

Rojas-Peña A, et al, 2014
Extracorporeal support (ECS) can be routinely implemented in controlled DCDD. In our experience, the organs provided per donor was 2.59. Widely applied, EDCDD could result in more donor organs, especially when applied to DCDD in uncontrolled conditions.

Mesnard B et al, 2022
One simultaneous pancreas-kidney transplantation was performed.

References:

Oniscu GC, Mehew J, Butler AJ, Sutherland A, Gaurav R, Hogg R, Currie I, Jones M, Watson CJE. Improved Organ Utilization and Better Transplant Outcomes With In Situ Normothermic Regional Perfusion in Controlled Donation After Circulatory Death. Transplantation. 2022 Aug 22. doi: 10.1097/TP.0000000000004280. Epub ahead of print. PMID: 35993664.

Richards JA, Roberts JL, Fedotovs A, Paul S, Cottee S, Defries G, et al. Outcomes for circulatory death and brainstem death pancreas transplantation with or without use of normothermic regional perfusion. British Journal of Surgery. 2021;108(12):1406-8.

Minambres E, Suberviola B, Dominguez-Gil B, Rodrigo E, Ruiz-San Millan JC, Rodriguez-San Juan JC, et al. Improving the Outcomes of Organs Obtained From Controlled Donation After Circulatory Death Donors Using Abdominal Normothermic Regional Perfusion. American Journal of Transplantation. 2017;17(8):2165-72.

Oniscu GC, Randle LV, Muiesan P, Butler AJ, Currie IS, Perera MT, et al. In situ normothermic regional perfusion for controlled donation after circulatory death--the United Kingdom experience. American Journal of Transplantation. 2014;14(12):2846-54.

Butler AJ, Randle LV, Watson CJ. Normothermic regional perfusion for donation after circulatory death without prior heparinization. Transplantation. 2014;97(12):1272-8.

Magliocca JF, Magee JC, Rowe SA, Gravel MT, Chenault RH, 2nd, Merion RM, et al. Extracorporeal support for organ donation after cardiac death effectively expands the donor pool. J Trauma. 2005;58(6):1095-101; discussion 101-2.

Farney AC, Singh RP, Hines MH, Rogers J, Hartmann EL, Reeves-Daniel A, Gautreaux MD, Iskandar SS, Adams PL, Stratta RJ. Experience in renal and extrarenal transplantation with donation after cardiac death donors with selective use of extracorporeal support. J Am Coll Surg. 2008 May;206(5):1028-37; discussion 1037. doi: 10.1016/j.jamcollsurg.2007.12.029. Epub 2008 Mar 17. PMID: 18471749.

Rojas-Peña A, Sall LE, Gravel MT, et al. Donation after circulatory determination of death: the university of Michigan experience with extracorporeal support. Transplantation. 2014;98:328–334.

Mesnard B, Cantarovich D, Martin-Lefevre L, Rigaud J, Blancho G, Karam G, Badet L, Antoine C, Branchereau J. First French combined kidney/pancreas transplantation from controlled donation after circulatory arrest (Maastricht III). Prog Urol. 2022 Jan;32(1):1-2. doi: 10.1016/j.purol.2021.10.001. Epub 2021 Nov 10. PMID: 34772617.

PRELIMINARY DRAFT

PRELIMINARY DRAFT

2- ISLET TRANSPLANTATION
IN-SITU NORMOTHERMIC REGIONAL PERFUSION (NRP)

PICO QUESTIONS

1. Is normothermic regional perfusion a reliable and reproducible method for donation after controlled circulatory death in the scenario of islet transplantation?
2. For islet transplantation, is normothermic regional perfusion compatible with the procurement of other abdominal (kidneys, liver) organs?
3. For islet transplantation, is normothermic regional perfusion compatible with the procurement of other thoracic (heart, lungs) organs?
4. Should post-mortem normothermic regional perfusion be run for a maximum of 3 hours in the context of islet transplantation?
5. Valid parameters (machine perfusion, laboratory analysis and functional warm ischemia time) should be defined to assess the quality of the pancreatic graft before deciding the suitability/validity of the organ for islet transplant?
6. Could normothermic regional perfusion in donation after circulatory death improve graft and patient outcomes compared with in situ cooling and rapid procurement in islet transplantation?
7. Does the normothermic regional perfusion have the potential to expand the donor pool for islet transplantation?

PRELIMINARY DRAFT

PICO 1: Is normothermic regional perfusion a reliable and reproducible method for donation after controlled circulatory death in the scenario of islet transplantation?

RECOMMENDATION/STATEMENT: Normothermic regional perfusion is a reliable and reproducible method for donation after controlled circulatory death in the scenario of islet transplantation.

Quality of evidence: Low

Strength of recommendation: Strong for

Analysis of the literature:
A total of two studies reporting outcome after cDCD islet transplantation using normothermic regional perfusion have been published so far.

Oniscu GC et al, 2022 (CASE SERIES, 1 kidney and pancreas islets, and 2 pancreas islets)
Oniscu et al published a retrospective analysis of UK adult cDCD donors' where at least 1 abdominal organ was accepted for transplantation between January 1, 2011, and December 31, 2019.
There were 1906 donors who had their pancreas offered, of which 413 (22%) proceeded to transplant. Thirty-one NRP pancreases were transplanted (28 as SPK, 1 as kidney and pancreas islets, and 2 as pancreas islets). Three-hundred eighty-two non-NRP pancreases were transplanted (312 as SKP, 3 as kidney and pancreas islet, 41 as pancreas alone, and 26 as pancreas islets).
The use of NRP influenced the probability of pancreas transplantation (SPK) with a 1.6 odds ratio for donors undergoing NRP compared with those who did not (LRT P = 0.0611).

Oniscu GC et al, 2014 (CASE SERIES, 2 islet isolation, 1 research).
Five pancreata were recovered from these donors. Two simultaneous pancreas–kidney transplants were performed in type I diabetic patients aged 52 and 34 years old, with primary renal and pancreatic function.. Two pancreata were sent for islet isolation. One achieved a good islet yield and was transplanted while the second one was fibrotic and achieved a lower yield than required for transplantation. The fifth pancreas was initially accepted for transplantation as a solid organ by two centers but later turned down on logistic reasons and was eventually sent for research. The remainder of the pancreata were not recovered (three citing a long WIT, eight donor age and five donor history as the reasons for non use).

References:
Oniscu GC, Mehew J, Butler AJ, Sutherland A, Gaurav R, Hogg R, Currie I, Jones M, Watson CJE. Improved Organ Utilization and Better Transplant Outcomes with In Situ Normothermic Regional Perfusion in Controlled Donation After Circulatory Death. Transplantation. 2022 Aug 22. doi: 10.1097/TP.0000000000004280. Epub ahead of print. PMID: 35993664.

Oniscu GC, Randle LV, Muiesan P, Butler AJ, Currie IS, Perera MT, et al. In situ normothermic regional perfusion for controlled donation after circulatory death--the United Kingdom experience. American Journal of Transplantation. 2014;14(12):2846-54.

PICO 2: For islet transplantation, is normothermic regional perfusion compatible with the procurement of other abdominal (kidneys, liver) organs?

PRELIMINARY DRAFT

RECOMMENDATION/STATEMENT: For islet transplantation, normothermic regional perfusion is compatible with the procurement of other abdominal (kidneys, liver) organs.

Quality of evidence: Low

Strength of recommendation: Strong for

Analysis of the literature:
The two published studies describing the use of normothermic regional perfusion in the context of cDCD islet transplant reported recovery and utilization of the liver and kidney for transplantation.

Oniscu GC et al, 2022
Impact of NRP on Organ Utilization: Nine-thousand nine-hundred one adult DCD donors consented for donation in the period considered, of which 2587 (26%) were excluded because of prolonged time to asystole, and 12 NRP cases were removed, giving 7302 donors. Of these 7302 donors, 6440 had at least 1 organ (abdominal or cardiothoracic) offered for transplantation and 4716 had at least 1 abdominal organ accepted for transplantation before retrieval and were considered in the subsequent analyses.
When applying organ-specific criteria, 4276 donors had the liver offered, 1906 donors had the pancreas offered, and 4622 donors had at least 1 kidney offered for transplantation before retrieval (9223 kidneys offered in total).

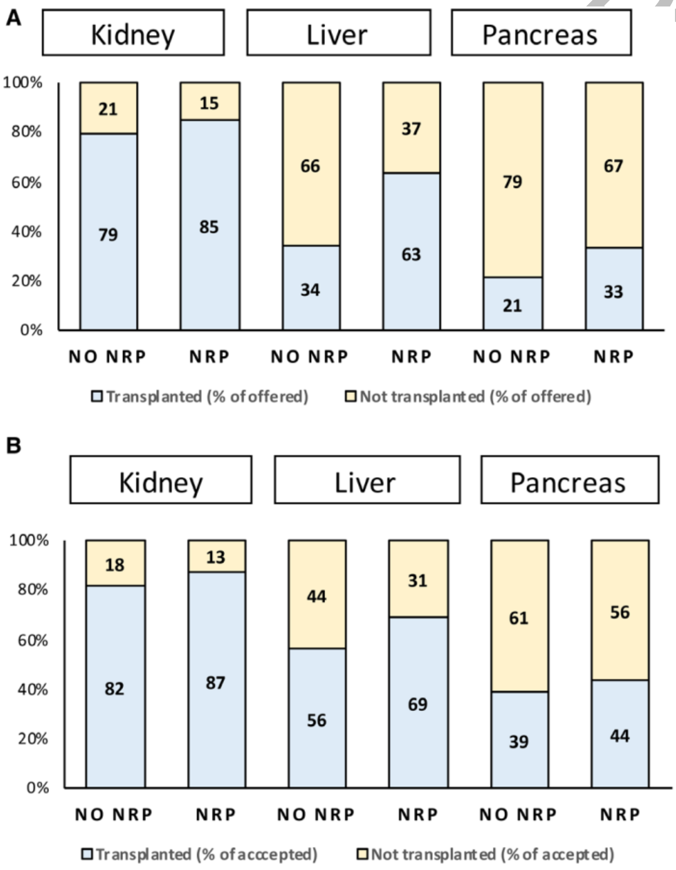

PRELIMINARY DRAFT

FIGURE. Individual organ utilization according to the use of NRP. A, Percentage of organs transplanted of those offered. B, Percentage of organs transplanted of those retrieved. NRP, normothermic regional perfusion.

The use of NRP (not adjusted for differences in donor risk profile) was associated with improved organ utilization rates for liver, kidney, and pancreas from the total number of organ offers as illustrated in Figure A. We also undertook a comparison of the number of organs accepted that were retrieved and transplanted (Figure B) and found a significant increase in transplantation rates for livers (P = 0.006, chi-square) and kidneys (P = 0.03, chi-square) if NRP was used.

Oniscu GC et al, 2014
Sixty-three organs were recovered achieving an organ recovery rate of three organs/donor compared to the national DCD average of 2.6 organs/donor. Forty-nine patients received a transplant. The abdominal organ transplant activity is detailed in Table 2.
Two combined pancreas– kidney transplants, one islet transplant and three double lung transplants were performed with primary function. Eleven livers were recovered and transplanted.

Table 2: Individual center normothermic regional perfusion retrieval and organ transplant activity

	Number of			
Transplant center	Donors	Livers	Kidneys	Pancreata
Birmingham	3	2	5[1]	–
Cambridge	9	4	16[2]	2
Edinburgh	9	5	17[3]	1[4]
All	21	11	38	3

[1]One donor had a previous nephrectomy.
[2]Three double kidney transplants, two discarded, two combined pancreas and kidney transplants.
[3]One double kidney transplant and one discarded.
[4]One pancreas used for research, one pancreas for islet isolation with insufficient yield and one pancreas used for islets.

References:

Oniscu GC, Mehew J, Butler AJ, Sutherland A, Gaurav R, Hogg R, Currie I, Jones M, Watson CJE. Improved Organ Utilization and Better Transplant Outcomes With In Situ Normothermic Regional Perfusion in Controlled Donation After Circulatory Death. Transplantation. 2022 Aug 22. doi: 10.1097/TP.0000000000004280. Epub ahead of print. PMID: 35993664.

Oniscu GC, Randle LV, Muiesan P, Butler AJ, Currie IS, Perera MT, et al. In situ normothermic regional perfusion for controlled donation after circulatory death--the United Kingdom experience. American Journal of Transplantation. 2014;14(12):2846-54.

PICO 3: For islet transplantation, is normothermic regional perfusion compatible with the procurement of other thoracic (heart, lungs) organs?

RECOMMENDATION/STATEMENT: For islet transplantation, normothermic regional perfusion is compatible with the procurement of other thoracic (heart, lungs) organs.

Quality of evidence: low – Expert opinion

Strength of recommendation: Strong for

PRELIMINARY DRAFT

Analysis of the literature:

Oniscu GC et al, 2022

Impact of NRP on Organ Utilization: Nine-thousand nine-hundred one adult DCD donors consented for donation in the period considered, of which 2587 (26%) were excluded because of prolonged time to asystole, and 12 NRP cases were removed as detailed above, giving 7302 donors. Of these 7302 donors, 6440 had at least 1 organ (abdominal or cardiothoracic) offered for transplantation and 4716 had at least 1 abdominal organ accepted for transplantation before retrieval and were considered in the subsequent analyses. Of the 4716 donors considered, there were 1862 donors who had at least 1 lung offered for transplantation. There were 379 donors who had their heart offered for transplantation, of which 42 underwent A-NRP (48% were transplanted) and 336 did not undergo NRP (23% were transplanted). The UK DCD Heart program began in February 2015 and was only in operation at certain transplant centers during the period considered. Although the utilization of the cardiothoracic organs was not a direct focus of this article, during this study, NRP has facilitated cDCD heart transplantation.

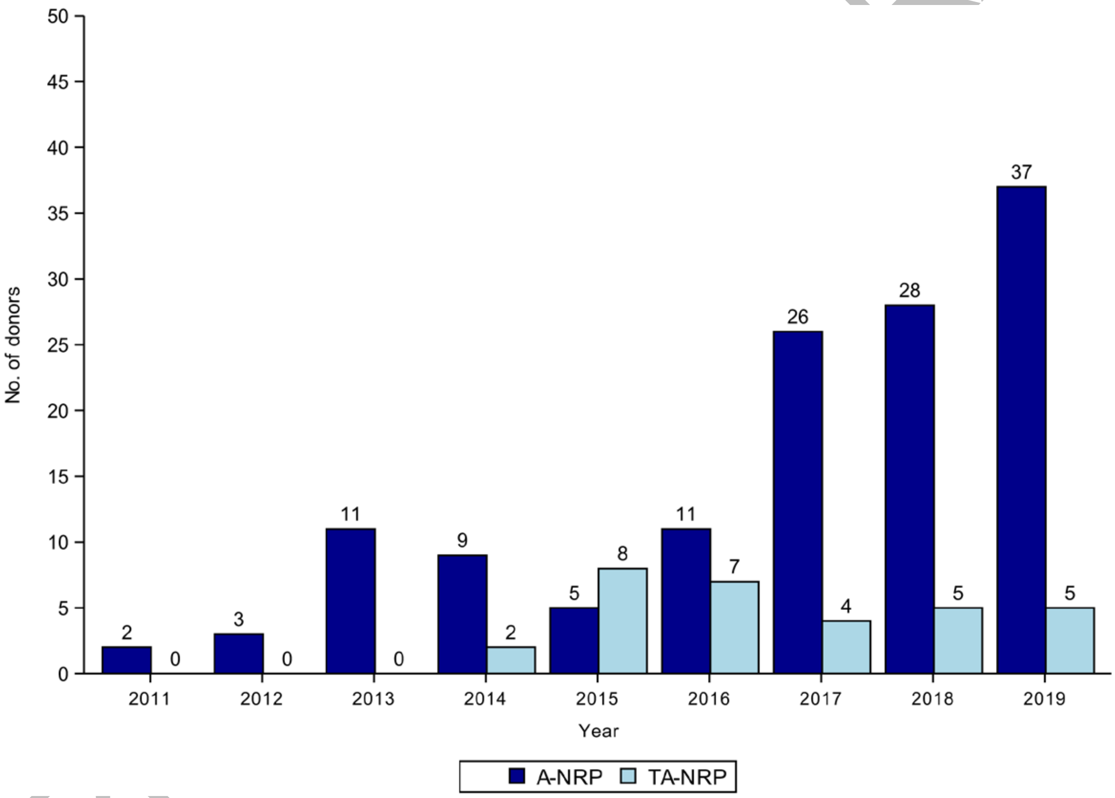

FIGURE. NRP use during the study period (2011–2019) by y and whether A-NRP or TA-NRP was used. A-NRP, abdominal normothermic regional perfusion; NRP, normothermic regional perfusion; TA-NRP, thoraco-abdominal normothermic regional perfusion.

Oniscu GC et al, 2014

Two combined pancreas– kidney transplants, one islet transplant and three double lung transplants were performed with primary function.

No information is provided in relation to the retrieval of the heart.

References:

Oniscu GC, Mehew J, Butler AJ, Sutherland A, Gaurav R, Hogg R, Currie I, Jones M, Watson CJE. Improved Organ Utilization and Better Transplant Outcomes With In Situ Normothermic Regional Perfusion in Controlled Donation After Circulatory Death. Transplantation. 2022 Aug 22. doi: 10.1097/TP.0000000000004280. Epub ahead of print. PMID: 35993664.

Oniscu GC, Randle LV, Muiesan P, Butler AJ, Currie IS, Perera MT, et al. In situ normothermic regional perfusion for controlled donation after circulatory death--the United Kingdom experience. American Journal of Transplantation. 2014;14(12):2846-54.

PRELIMINARY DRAFT

PICO 4: Should post-mortem normothermic regional perfusion be run for a maximum of 3 hours in the context of islet transplantation?

RECOMMENDATION/STATEMENT: In the context of islet transplantation normothermic regional perfusion should be maintained between 1 hour and 3 hours.

Quality of evidence: Low – Expert opinion
Expert opinion: Normothermic regional perfusion should be performed for more than 1h but less than 3 hours. Extended perfusion should be avoided to avoid graft edema.
The recommended time on CEC/ECMO would be 60 minutes, which can be extended to 120 minutes if necessary. It is considered that the dissection of the vascular structures and the preparation of the liver/pancreas should be carried out during this time, to avoid returning to normothermia once the extraction of the thoracic organs (lung and heart) has been carried out.

Strength of recommendation: Strong for

Analysis of the literature:
Oniscu GC et al, 2022
Abdominal-NRP was undertaken for 2h with postmortem cannulation of abdominal vena cava and aorta (or iliac vein and artery) with an endovascular or external clamp occluding the descending thoracic aorta. The perfusion was stopped after 30 to 60 min when thoraco-abdominal NRP (TA-NRP) was undertaken and the heart allowed support to the limited thoraco-abdominal circulation while its function was evaluated; otherwise, perfusion continued for 2h. NRP was considered to have failed when a circulation could not be established or maintained for at least 30min to allow the initial set of blood tests to be collected and analyzed.

Oniscu GC et al, 2014
2 h of normothermic regional perfusion
NRP duration 2 h (34 min–2 h 36 min)
Perfusion pressure:1.7 to 4L/min, Tº: 37ºC. Whole blood. Oxygen air mixture

References:
Oniscu GC, Mehew J, Butler AJ, Sutherland A, Gaurav R, Hogg R, Currie I, Jones M, Watson CJE. Improved Organ Utilization and Better Transplant Outcomes With In Situ Normothermic Regional Perfusion in Controlled Donation After Circulatory Death. Transplantation. 2022 Aug 22. doi: 10.1097/TP.0000000000004280. Epub ahead of print. PMID: 35993664.

Oniscu GC, Randle LV, Muiesan P, Butler AJ, Currie IS, Perera MT, et al. In situ normothermic regional perfusion for controlled donation after circulatory death--the United Kingdom experience. American Journal of Transplantation. 2014;14(12):2846-54.

PICO 5: Should valid parameters (machine perfusion -monitoring flow and temperature-, analytical/biochemical parameters and functional warm ischemia time) be defined to assess the quality of the pancreatic graft before deciding the suitability/validity of the organ for islet transplant?

RECOMMENDATION/STATEMENT: Valid parameters (machine perfusion -monitoring flow and temperature-, analytical/biochemical parameters and functional warm ischemia time) should be defined to assess the quality of the pancreatic graft before deciding the suitability/validity of the organ for islet transplant.

Donation parameters:

- Total warm ischemia time < 60 minutes. (From withdrawal of ventilatory support to start of preservation).
- Functional or true warm ischemia time < 30 minutes. (From systolic blood pressure < 60 mmHg to start of preservation)
- Hemodynamic instability (SBP < 60mmHg) prior to WLST < 60 minutes.
- Preferable cold ischemia time < 8 hours.
WLST, withdraw life-sustaining therapies

Functional parameters

- Renal biochemistry prior to WLST (Cr < 2.0 mg/dl), after diagnosis of death (Cr < 2.5 mg/dl) and evolution curve during PAN every 30 minutes with a final Cr < 2.5 mg/dl.
- Initial liver biochemistry (before LLST and diagnosis of death): AST, ALT < 3 times the normal value.
- Evolutionary and final liver biochemistry: AST, ALT < 4 times the normal value.
- Initial and final pancreatic biochemistry (amylase and/or lipase): < 3 times the normal value.

Quality of evidence: Low – Expert opinion

Strength of recommendation: Strong for

Analysis of the literature:
Oniscu GC et al, 2022
- Median TWIT (min) (IQR): 31 (26–41).
- Median FWIT (min) (IQR): 23 (18–28).

PRELIMINARY DRAFT

TWIT is defined as the time from treatment withdrawal to the start of NRP/start of in situ cold perfusion for non-NRP group (missing data: 613 [13%] for non NRP group and 3 [2%] for NRP group).
FWIT is defined as the time from systolic BP reaching 50 mm Hg to the start of NRP/start of in situ cold perfusion for non-NRP group (missing data: 1435 [32%] for non-NRP and 46 [28%] for NRP group).
No functional parameters data is present.
A-NRP was undertaken for 2 h with postmortem cannulation of abdominal vena cava and aorta (or iliac vein and artery) with an endovascular or external clamp occluding the descending thoracic aorta. The perfusion was stopped after 30 to 60 min when thoraco-abdominal NRP (TA-NRP) was undertaken and the heart allowed support to the limited thoraco-abdominal circulation while its function was evaluated; otherwise, perfusion continued for 2 h. NRP was considered to have failed when a circulation could not be established or maintained for at least 30 min to allow the initial set of blood tests to be collected and analyzed.

Oniscu GC et al, 2014
Two simultaneous pancreas–kidney transplants were performed in type I diabetic patients aged 52 and 34 years old, with primary renal and pancreatic function. The CITs for the pancreata were 8 h 52 min and 7 h 32 min, respectively.
- Withdrawal to asystole: 13 min (6 min–249 min)
- Asystole to NRP: 16 min (10 min–23 min)
- Functional warm ischemia time: 26 min (13 min–48 min)
- NRP duration: 2 h (34 min–2 h 36 min)

Abdominal organ function and homeostasis during NRP were monitored every 30 min using blood gases (pH, pO2, pCO2, bicarbonate, base excess, lactate), and every 30–60 min for hematology (hemoglobin, hematocrit, white cell count, platelet count, activated partial thromboplastin time) and biochemistry (alanine transaminase [ALT], bilirubin, alkaline phosphatase, urea, creatinine, glucose, sodium and potassium).
The current criteria for DCD selection were observed, with a functional warm ischemia time (WIT; from systolic BP<50mmHg to start of perfusion) of less than 30 min for the liver and pancreas, and 1 h for kidneys. In addition, as suggested by the Spanish experience in uncontrolled DCD liver donation, an ALT <3 times the upper limit of normal at the initiation of NRP and <4 times the upper limit of normal at the end of NRP were considered when selecting the liver grafts (7). There were no additional donor exclusion criteria and we attempted NRP in all donors that the teams could attend. Organs were allocated in keeping with the current United Kingdom allocation criteria

References:
Oniscu GC, Mehew J, Butler AJ, Sutherland A, Gaurav R, Hogg R, Currie I, Jones M, Watson CJE. Improved Organ Utilization and Better Transplant Outcomes With In Situ Normothermic Regional Perfusion in Controlled Donation After Circulatory Death. Transplantation. 2022 Aug 22. doi: 10.1097/TP.0000000000004280. Epub ahead of print. PMID: 35993664.

Oniscu GC, Randle LV, Muiesan P, Butler AJ, Currie IS, Perera MT, et al. In situ normothermic regional perfusion for controlled donation after circulatory death--the United Kingdom experience. American Journal of Transplantation. 2014;14(12):2846-54.

PRELIMINARY DRAFT

PICO 6: Could normothermic regional perfusion in donation after circulatory death improve graft and patient outcomes compared with in situ cooling and rapid procurement in islet transplantation?

RECOMMENDATION/STATEMENT: For whole pancreas transplantation normothermic regional perfusion in donation after circulatory death (DCD) might improve the graft and patient outcomes compare with in situ cooling and rapid procurement.

Quality of evidence: Very Low – Expert opinion

Strength of recommendation: Strong for

Analysis of the literature:

Oniscu GC et al, 2022
Thirty-one NRP pancreases were transplanted (28 as SPK, 1 as kidney and pancreas islets, and 2 as pancreas islets).
Three-hundred eighty-two non-NRP pancreases were transplanted (312 as SKP, 3 as kidney and pancreas islet, 41 as pancreas alone, and 26 as pancreas islets).
There was no significant difference between the groups (P = 0.2889) in terms of risk-adjusted 12-mo graft failure.
There was no impact of the functional warm ischemia time in the overall model with no significant difference in graft survival for each min increase (P = 0.826).

Oniscu GC et al, 2014
Two pancreata were sent for islet isolation. One achieved a good islet yield and was transplanted while the second one was fibrotic and achieved a lower yield than required for transplantation. The fifth pancreas was initially accepted for transplantation as a solid organ by two centers but later turned down on logistic reasons and was eventually sent for research. The remainder of the pancreata were not recovered (three citing a long WIT, eight donor age and five donor history as the reasons for non use).

DCD data with in situ cooling and rapid procurement were not provided

References:
Oniscu GC, Mehew J, Butler AJ, Sutherland A, Gaurav R, Hogg R, Currie I, Jones M, Watson CJE. Improved Organ Utilization and Better Transplant Outcomes With In Situ Normothermic Regional Perfusion in Controlled Donation After Circulatory Death. Transplantation. 2022 Aug 22. doi: 10.1097/TP.0000000000004280. Epub ahead of print. PMID: 35993664.

PRELIMINARY DRAFT

Oniscu GC, Randle LV, Muiesan P, Butler AJ, Currie IS, Perera MT, et al. In situ normothermic regional perfusion for controlled donation after circulatory death--the United Kingdom experience. American Journal of Transplantation. 2014;14(12):2846-54.

PICO 7: Does the normothermic regional perfusion have the potential to expand the donor pool for islet transplantation?

RECOMMENDATION/STATEMENT: Normothermic regional perfusion has the potential to expand the donor pool for islet transplantation.

Quality of evidence: very low – Expert opinion

Strength of recommendation: Strong for

Analysis of the literature:
Oniscu GC et al, 2022
NRP is an independent factor in increasing organ utilization for all abdominal organs.
This is a retrospective analysis of UK adult cDCD donors' where at least 1 abdominal organ was accepted for transplantation between January 1, 2011, and December 31, 2019.
Results: A mean of 3.3 organs was transplanted when NRP was used compared with 2.6 organs per donor when NRP was not used. When adjusting for organ-specific donor risk profiles, the use of NRP increased the odds of all abdominal organs being transplanted by 3-fold for liver (P < 0.0001; 95% confidence interval [CI], 2.20-4.29), 1.5-fold for kidney (P = 0.12; 95% CI, 0.87-2.58), and 1.6-fold for pancreas (P = 0.0611; 95% CI, 0.98-2.64). Twelve-mo liver transplant survival was superior for recipients of a cDCD NRP graft with a 51% lower risk-adjusted hazard of transplant failure (HR = 0.494). In risk-adjusted analyses, NRP kidneys had a 35% lower chance of developing delayed graft function than non-NRP kidneys (odds ratio, 0.65; 95% CI, 0.465-0.901)' and the expected 12-mo estimated glomerular filtration rate was 6.3 mL/min/1.73 m2 better if abdominal NRP was used (P < 0.0001).
The use of NRP (not adjusted for differences in donor risk profile) was associated with improved organ utilization rates for liver, kidney, and pancreas from the total number of organ offers. They found a significant increase in transplantation rates for livers (P = 0.006, chi-square) and kidneys (P = 0.03, chi-square) if NRP was used.
Utilization rates, unadjusted for donor risk profile, were similar for donors who underwent A-NRP or TA-NRP, despite the TA-NRP donors being significantly younger (median age 40 y old versus 54 y old for A-NRP donors), but the numbers are rather small to draw definitive conclusions.

Oniscu GC et al, 2014
The results of this study are encouraging on many accounts such as organ function and recovery rate. While NRP will not change organ allocation it may lead to a wider utilization and sharing of organs currently discarded or indeed lead to more liberal acceptance criteria

References:
Oniscu GC, Mehew J, Butler AJ, Sutherland A, Gaurav R, Hogg R, Currie I, Jones M, Watson CJE. Improved Organ Utilization and Better Transplant Outcomes With In Situ Normothermic

PRELIMINARY DRAFT

Regional Perfusion in Controlled Donation After Circulatory Death. Transplantation. 2022 Aug 22. doi: 10.1097/TP.0000000000004280. Epub ahead of print. PMID: 35993664.

Oniscu GC, Randle LV, Muiesan P, Butler AJ, Currie IS, Perera MT, et al. In situ normothermic regional perfusion for controlled donation after circulatory death--the United Kingdom experience. American Journal of Transplantation. 2014;14(12):2846-54.

PRELIMINARY DRAFT
