# Supplementary material for: European Society for Organ Transplantation (ESOT) Consensus Statement on the Role of Pancreas Machine Perfusion to Increase the Donor Pool for Beta Cell Replacement Therapy
Source: Transpl Int. 2023 Jul 19;36:11374. doi: 10.3389/ti.2023.11374 (PMC10402633; doi:10.3389/ti.2023.11374)
Supplement: Supplementary file 3 [file DataSheet3.pdf]

Appendix S3
Studies included in review
(n = 46)

1. Augier D, Boucard JP, Pascal JP, Ribet A, Vaysse N. Relationships between blood flow and secretion in the isolated perfused canine pancreas. J Physiol (1972) 221(1):55-69. doi:10.1113/jphysiol.1972.sp009738

2. Barlow AD, Hamed MO, Mallon DH, Brais RJ, Gribble FM, Scott MA, et al. Use of Ex Vivo Normothermic Perfusion for Quality Assessment of Discarded Human Donor Pancreases. Am J Transplant (2015) 15(9):2475-2482. doi:10.1111/ajt.13303

3. Branchereau J, Renaudin K, Kervella D, Bernadet S, Karam G, Blancho G, et al. Hypothermic pulsatile perfusion of human pancreas: Preliminary technical feasibility study based on histology. Cryobiology (2018) 85:56-62. doi: 10.1016/j.cryobiol.2018.10.002

4. Brynger H. Twenty-four-hour preservation of the duct-ligated canine pancreatic allograft. Eur Surg Res (1975) 7(6):341-354. doi:10.1159/000127819

5. Butler AJ, Randle LV, Watson CJ. Normothermic regional perfusion for donation after circulatory death without prior heparinization. Transplantation (2014) 97(12):1272-1278. doi:10.1097/TP.0000000000000082

6. de Gruyl J, Westbroek DL, Macdicken I, Ridderhof E, Verschoor L, van Strik R. Cryoprecipitated plasma perfusion preservation and cold storage preservation of duct-ligated pancreatic allografts. Br J Surg (1977) 64(7):490-493. doi:10.1002/bjs.1800640711

7. Doppenberg JB, Leemkuil M, Engelse MA, Krikke C, de Koning EJP, Leuvenink HGD. Hypothermic oxygenated machine perfusion of the human pancreas for clinical islet isolation: a prospective feasibility study. Transpl Int (2021) 34(8):1397-1407. doi:10.1111/tri.13927

8. Eckhauser F, Knol JA, Porter-Fink V, Lockery D, Edgcomb L, Strodel WE, et al. Ex vivo normothermic hemoperfusion of the canine pancreas: applications and limitations of a modified experimental preparation. J Surg Res (1981) 31(1):22-37. doi: 10.1016/0022-4804(81)90026-3

9. Eloy MR, Kachelhoffer J, Pousse A, Dauchel J, Grenier JF. Ex vivo vascular perfusion of the isolated canine pancreas. Experimental procedure, haemodynamic data and experimental applications. Eur Surg Res (1974) 6(6):341-353. doi:10.1159/000127741

10. Farney AC, Singh RP, Hines MH, Rogers J, Hartmann EL, Reeves-Daniel A, et al. Experience in renal and extrarenal transplantation with donation after cardiac death donors with selective use of extracorporeal support. J Am Coll Surg (2008) 206(5):1028-37; discussion 1037. doi: 10.1016/j.jamcollsurg.2007.12.029.

11. Ferrer-Fàbrega J, Muñoz R, Ruiz J, Casanova D, Sánchez-Bueno F, Pérez-Daga et al. Pancreas transplantation from donors declared death by circulatory criteria: Initial experience in Spain. Transplant International (2021) 34(SUPPL 1): 173-174.

12. Ferrer-Fàbrega J, Muñoz R, Ruiz J, Casanova D, Sánchez-Bueno F, Pérez-Daga et al. Controlled Donation After Circulatory Death Pancreas Transplantation in Spain. Initial Experience [abstract]. Am J Transplant (2021) 21 (suppl 3). https://atcmeetingabstracts.com/abstract/controlled-donation-after-circulatory-death-pancreas-transplantation-in-spain-initial-experience/.

13. Florack G, Sutherland DE, Heil J, Squifflet JP, Najarian JS. Preservation of canine segmental pancreatic autografts: cold storage versus pulsatile machine perfusion. J Surg Res (1983) 34(5):493-504. doi:10.1016/0022-4804(83)90101-4

14. Hamaoui K, Gowers S, Sandhu B, Vallant N, Cook T, Boutelle M, et al. Development of pancreatic machine perfusion: translational steps from porcine to human models. J Surg Res (2018) 223:263-274. doi:10.1016/j.jss.2017.11.052

15. Hanf W, Codas R, Meas-Yedid V, Berthiller J, Buron F, Chauvet C, et al. Kidney graft outcome and quality (after transplantation) from uncontrolled deceased donors after cardiac arrest. Am J Transplant (2012) 12(6):1541-1550. doi:10.1111/j.1600-6143.2011.03983.x

16. Jochmans I, Hessheimer AJ, Neyrinck AP, Paredes D, Bellini MI, Dark JH, et al. Consensus statement on normothermic regional perfusion in donation after circulatory death: Report from the European Society for Organ Transplantation's Transplant Learning Journey. Transpl Int (2021) 34(11):2019-2030. doi:10.1111/tri.13951

17. Karcz M, Cook HT, Sibbons P, Gray C, Dorling A, Papalois V. An ex-vivo model for hypothermic pulsatile perfusion of porcine pancreata: hemodynamic and morphologic characteristics. Exp Clin Transplant (2010) 8(1):55-60

18. Kelly AC, Smith KE, Purvis WG, Min CG, Weber CS, Cooksey AM, et al. Oxygen Perfusion (Persufflation) of Human Pancreata Enhances Insulin Secretion and Attenuates Islet Proinflammatory Signaling. Transplantation (2019) 103(1):160-167. doi:10.1097/TP.0000000000002400

19. Kenmochi T, Asano T, Nakagouri T, Enomoto K, Isono K, Horie H. Prediction of viability of ischemically damaged canine pancreatic grafts by tissue flow rate with machine perfusion. Transplantation (1992) 53(4):745-750. doi:10.1097/00007890-199204000-00007

20. Kuan KG, Wee MN, Chung WY, Kumar R, Mees ST, Dennison A, et al. A Study of Normothermic Hemoperfusion of the Porcine Pancreas and Kidney. Artif Organs (2017) 41(5):490-495. doi:10.1111/aor.12770

21. Kumar R, Chung WY, Runau F, Isherwood JD, Kuan KG, West K, et al. Ex vivo normothermic porcine pancreas: A physiological model for preservation and

transplant study. Int J Surg (2018) 54(Pt A):206-215. doi:10.1016/j.ijsu.2018.04.057

22. Leemkuil M, Lier G, Engelse MA, Ploeg RJ, de Koning EJP, 't Hart NA, et al. Hypothermic Oxygenated Machine Perfusion of the Human Donor Pancreas. Transplant Direct (2018) 4(10):e388. Published 2018 Sep 7. doi:10.1097/TXD.0000000000000829

23. Leeser DB, Bingaman AW, Poliakova L, Shi Q, Gage F, Bartlett ST, et al. Pulsatile pump perfusion of pancreata before human islet cell isolation. Transplant Proc (2004) 36(4):1050-1051. doi:10.1016/j.transproceed.2004.04.041

24. Magliocca JF, Magee JC, Rowe SA, Gravel MT, Chenault RH 2nd, Merion RM, et al. Extracorporeal support for organ donation after cardiac death effectively expands the donor pool. J Trauma (2005) 58(6):1095-1102. doi:10.1097/01.ta.0000169949.82778.df

25. Mazilescu LI, Parmentier C, Kalimuthu SN, Ganesh S, Kawamura M, Goto T, et al. Normothermic ex situ pancreas perfusion for the preservation of porcine pancreas grafts. Am J Transplant (2022) 22(5):1339-1349. doi:10.1111/ajt.17019

26. Mesnard B, Cantarovich D, Martin-Lefevre L, Rigaud J, Blancho G, Karam G, et al. First French combined kidney/pancreas transplantation from controlled donation after circulatory arrest (Maastricht III). Prog Urol (2022) 32(1):1-2. doi:10.1016/j.purol.2021.10.001

27. Miñambres E, Suberviola B, Dominguez-Gil B, Rodrigo E, Ruiz-San Millan JC, Rodríguez-San Juan JC, et al. Improving the Outcomes of Organs Obtained From Controlled Donation After Circulatory Death Donors Using Abdominal Normothermic Regional Perfusion. Am J Transplant (2017) 17(8):2165-2172. doi:10.1111/ajt.14214

28. Nassar A, Liu Q, Walsh M, Quintini C. Normothermic Ex Vivo Perfusion of Discarded Human Pancreas. Artif Organs (2018) 42(3):334-335. doi:10.1111/aor.12985

29. O'Malley VP, Keyes DM, Postier RG. The fluosol-perfused isolated canine pancreas: a model for the study of blood component effects in acute pancreatitis. J Surg Res (1986) 40(3):210-215. doi:10.1016/0022-4804(86)90153-8

30. Ogbemudia AE, Hakim G, Dengu F, El-Gilani F, Dumbill R, Mulvey J, et al. Development of ex situ normothermic reperfusion as an innovative method to assess pancreases after preservation. Transpl Int (2021) 34(9):1630-1642. doi:10.1111/tri.13990

31. Oniscu GC, Mehew J, Butler AJ, Sutherland A, Gaurav R, Hogg R, et al. Improved Organ Utilization and Better Transplant Outcomes With In Situ Normothermic Regional Perfusion in Controlled Donation After Circulatory Death. Transplantation (2023) 107(2):438-448. doi:10.1097/TP.0000000000004280

32. Oniscu GC, Randle LV, Muiesan P, Butler AJ, Currie IS, Perera MT, et al. In situ normothermic regional perfusion for controlled donation after circulatory death--the United Kingdom experience. Am J Transplant (2014) 14(12):2846-2854. doi:10.1111/ajt.12927

33. Pegg DE, Klempnauer J, Diaper MP, Taylor MJ. Assessment of hypothermic preservation of the pancreas in the rat by a normothermic perfusion assay. J Surg Res (1982) 33(3):194-200. doi:10.1016/0022-4804(82)90029-4

34. Prudhomme T, Kervella D, Ogbemudia AE, Gauttier V, Le Bas-Bernardet S, Minault D, et al. Successful pancreas allotransplantations after hypothermic machine perfusion in a novel diabetic porcine model: a controlled study. Transpl Int (2021) 34(2):353-364. doi:10.1111/tri.13797

35. Prudhomme T, Renaudin K, Lo Faro ML, Cantarovich D, Kervella D, Minault D, et al. Ex situ hypothermic perfusion of nonhuman primate pancreas: A feasibility study. Artif Organs (2020) 44(7):736-743. doi:10.1111/aor.13655

36. Reddy MS, Carter N, Cunningham A, Shaw J, Talbot D. Portal Venous Oxygen Persufflation of the Donation after Cardiac Death pancreas in a rat model is superior to static cold storage and hypothermic machine perfusion. Transpl Int (2014) 27(6):634-639. doi:10.1111/tri.12313

37. Richards JA, Roberts JL, Fedotovs A, Paul S, Cottee S, Defries G, et al. Outcomes for circulatory death and brainstem death pancreas transplantation with or without use of normothermic regional perfusion. Br J Surg (2021) 108(12):1406-1408. doi:10.1093/bjs/znab212

38. Richards, J., et al. The impact of normothermic regional perfusion on simultaneous kidney and pancreas transplantation. Transplant International (2021) 34(SUPPL 1): 205.

39. Richards, J., et al. Comparable outcomes for circulatory death and brain-stem death pancreas transplantation irrespective of the use of normothermic regional perfusion. Transplant International (2021) 34(SUPPL 1): 28.

40. Rojas-Peña A, Sall LE, Gravel MT, Cooley EG, Pelletier SJ, Bartlett RH, et al. Donation after circulatory determination of death: the university of michigan experience with extracorporeal support. Transplantation (2014) 98(3):328-334. doi:10.1097/TP.0000000000000070

41. Scott WE 3rd, O'Brien TD, Ferrer-Fabrega J, Avgoustiniatos ES, Weegman BP, Anazawa T, et al. Persufflation improves pancreas preservation when compared with the two-layer method. Transplant Proc (2010) 42(6):2016-2019. doi:10.1016/j.transproceed.2010.05.092

42. Taylor MJ, Baicu S, Greene E, Vazquez A, Brassil J. Islet isolation from juvenile porcine pancreas after 24-h hypothermic machine perfusion preservation. Cell Transplant (2010) 19(5):613-628. doi:10.3727/096368910X486316

43. Tersigni R, Toledo-Pereyra LH, Pinkham J, Najarian JS. Pancreaticoduodenal preservation by hypothermic pulsatile perfusion for twenty-four hours. Ann Surg (1975) 182(6):743-748. doi:10.1097/00000658-197512000-00016

44. Toledo-Pereyra LH, Valgee KD, Castellanos J, Chee M. Hypothermic pulsatile perfusion: its use in the preservation of pancreases for 24 to 48 hours before islet cell transplantation. Arch Surg (1980) 115(1):95-98. doi:10.1001/archsurg.1980.01380010081022

45. Wahlberg J, Southard JH, Belzer FO. Preservation-induced pancreatitis in an isolated perfused pancreas model in the dog. Transpl Int (1989);2(3):165-167. doi:10.1007/BF02414603

46. Weegman BP, Taylor MJ, Baicu SC, Scott WE 3rd, Mueller KR, Kitzmann JD, et al. Hypothermic Perfusion Preservation of Pancreas for Islet Grafts: Validation Using a Split Lobe Porcine Model. Cell Med (2012) 2(3):105-110. doi:10.3727/215517911X617897
