# Supplementary material for: European Society for Organ Transplantation (ESOT) Consensus Statement on the Role of Pancreas Machine Perfusion to Increase the Donor Pool for Beta Cell Replacement Therapy
Source: Transpl Int. 2023 Jul 19;36:11374. doi: 10.3389/ti.2023.11374 (PMC10402633; doi:10.3389/ti.2023.11374)
Supplement: Supplementary file 4 [file DataSheet1.pdf]

EVIDENCE REPORT

PICO

Population:
Pancreatic transplantation (whole organ transplantation)
Islet transplantation

Intervention:
Pancreatic transplantation: Hypothermic machine perfusion (HMP), normothermic machine perfusion (NMP), persufflation or in-situ normothermic regional perfusion

Comparator:
Preservation in cold storage

Outcomes:
Outcomes include
- graft characteristics
- perfusion parameters
- cold ischemia time
- overall patient survival
- overall graft survival
- insulin independence
- complications rate
- rejection rate
- macroscopic evaluation
- histological examination
- immunohistochemical analysis
- biochemical analysis
- islet mass isolated
- viability of islet
- IEQ / gram, OCR, MiRNA, cell free (insulin) DNA, GSIS, MouseTx
- IGLS-score improvement of patients dependent on IEQ/Kg

Pre-clinical setting (animal model)
- animal and graft characteristics
- perfusion parameters
- cold ischemia time
- animal survival
- graft survival
- islet mass isolated
- viability of islet

- complications rate and rejection rate.
- macroscopic evaluation
- histological examination
- immunohistochemical analysis
- biochemical analysis
- IEQ / gram, OCR, MiRNA, cell free (insulin) DNA, GSIS, MouseTx
- IGLS-score improvement of patients dependent on IEQ/Kg

Study design:
Any study design, clinical or pre-clinical

Exclusion criteria:

- Any language other than English

Search strategy and results

The Transplant Library (TL) was searched on May 30, 2022. The TL includes all randomised controlled trials and systematic reviews in the field of solid organ transplantation published as full text or in abstract form, sourced mainly from MEDLINE/PubMed and hand-searches of congress proceedings.

The search strategy used is as follows:

1. pancreas transplantation/
2. pancreas.ti,ab.
3. islets of langerhans transplantation/
4. islet.ti,ab.
5. or/1-4
6. Perfusion/
7. Organ Preservation/
8. persufflation.ti,ab.
9. (perfusion or preservation).ti,ab.
10. (two layer method or two-layer method or TLM).ti,ab.
11. or/6-10
12. 5 and 11

Searches identified 32 potentially relevant references.

Searches were expanded to include non-randomised studies. We searched MEDLINE and EMBASE from [enter date range] using the search strategy below.

1. pancreas transplantation/
2. (pancreas adj3 transplant$).mp.
3. islets of langerhans transplantation/
4. (islet adj2 transplant$).mp.
5. organ transplant$.ti.
6. (simultaneous pancreas kidney or simultaneous pancreas-kidney or SPK).ti,ab.
7. or/1-6
8. persufflation.ti,ab.
9. (two layer method or two-layer method or TLM).ti,ab.
10. Cardiopulmonary Bypass/ or Cardiopulmonary Bypass.ti,ab. or heart-lung bypass.ti,ab. or Extracorporeal Circulation/ or extracorporeal circulation.ti,ab. or Extracorporeal Membrane Oxygenation/ or extracorporeal membrane oxygenation.ti,ab. or ECMO.ti,ab. or regional perfusion.ti,ab. or machine perfusion.ti,ab. or perfusion/ or ex-situ perfusion.ti,ab. or ((in situ or in-situ) adj perfusion).ti,ab. or oxygenation.ti,ab. or hypothermic perfusion.ti,ab. or normothermic perfusion.ti,ab.
11. 8 or 9 or 10
12. 7 and 11

Searches identified 763 potentially relevant references.

Clinical practice guidelines

Boggi, U, Vistoli, F, Andres, A, et al. First World Consensus Conference on pancreas transplantation: Part II – recommendations. Am J Transplant. 2021; 21(Suppl. 3): 17– 59

DOI: https://doi.org/10.1111/ajt.16750

The First World Consensus Conference on Pancreas Transplantation provided 49 jury deliberations regarding the impact of pancreas transplantation on the treatment of diabetic patients, and 110 experts' recommendations for the practice of pancreas transplantation. It is stated that "No recommendation was drawn on the use of machine perfusion because of lack of clinical studies".

Systematic reviews

De Beule J, Vandendriessche K, Pengel LHM, et al. A systematic review and meta-analyses of regional perfusion in donation after circulatory death solid organ transplantation. Transpl Int. 2021 Nov;34(11):2046-2060.

DOI: https://dx.doi.org/10.1111/tri.14121

Jochmans I, Hessheimer AJ, Neyrinck AP, et al; ESOT Workstream 04 of the TLJ (Transplant Learning Journey) project. Consensus statement on normothermic regional perfusion in donation after circulatory death: Report from the European Society for Organ Transplantation's Transplant Learning Journey. Transpl Int. 2021 Nov;34(11):2019-2030

DOI: https://dx.doi.org/10.1111/tri.13951

The systematic review on NRP included three studies reporting on outcomes after DCD pancreas transplantation. All cases experienced immediate graft function. Two studies reported 100% 6-month and 1-year graft survival.

Prudhomme T, Kervella D, Le Bas-Bernardet S, et al. Ex situ Perfusion of Pancreas for Whole-Organ Transplantation: Is it Safe and Feasible? A Systematic Review. J Diabetes Sci Technol. 2020 Jan;14(1):120-134

DOI: https://dx.doi.org/10.1177/1932296819869312

The systematic review evaluated safety and feasibility of ex situ perfusion of pancreas for whole-organ transplantation of studies published up to 2018. Nine studies on hypothermic machine perfusion and 10 studies on normothermic machine perfusion were included. The evidence was summarized regarding the machine perfusion model, types of experimental model, anatomy, perfusion parameters, flushing and perfusion solution, length of perfusion, and comparison between static cold storage and perfusion.

van de Leemkolk FEM, Schurink IJ, et al. Abdominal Normothermic Regional Perfusion in Donation After Circulatory Death: A Systematic Review and Critical Appraisal. Transplantation. 2020;104(9):1776-1791.

DOI: https://dx.doi.org/10.1097/TP.0000000000003345

The review aimed to evaluate the clinical evidence for the use of aNRP regarding donor organ assessment, function and outcomes. Three studies reported on pancreas or islet transplantation: One studies on pancreas as whole organ transplant, three studies on simultaneous pancreas-kidney (SPK) transplants and one study on islet transplantation.

Kuan KG, Wee MN, Chung WY, et al. Extracorporeal machine perfusion of the pancreas: technical aspects and its clinical implications--a systematic review of experimental models. Transplant Rev (Orlando). 2016 Jan;30(1):31-47.

DOI: https://doi.org/10.1016/j.trre.2015.06.002

The systematic review summarizes the evidence of experimental models of pancreas machine perfusion (MP), its benefits, technical aspects and clinical implications. The literature search included studies up to 2014. Seven articles on normothermic MP and 13 articles on hypothermic MP were included (tables 1&2). The evidence regarding the anatomy, perfusion duration, pulsatile flow, pressure and flow rates, perfusate and perfusate oxygenation is presented.

Hilling DE, Bouwman E, Terpstra OT, Marang-Van De Mheen PJ. Effects of Donor-, Pancreas-, and Isolation-Related Variables on Human Islet Isolation Outcome: A Systematic Review. Cell Transplantation. August 2014:921-928.

DOI: https://doi.org/10.3727/096368913X666412

The systematic review assessed the impact of donor-, pancreas-, and isolation-related variables on successful human islet isolation outcome. The review included 74 retrospective studies. Higher pre- and postpurification islet yields and a higher proportion of successful islet isolations were obtained when pancreata were preserved with the two-layer method rather than University of Wisconsin solution in donors with shorter cold ischemia times.

Li X, Zhang J, Sang L, Chu Z, Dong M. Influence of the two-layer preservation method on human pancreatic islet isolation: a meta-analysis. Int J Artif Organs. 2015 Mar;38(3):117-25

DOI: https://dx.doi.org/10.5301/ijao.5000391

The objective the meta-analysis of 18 human studies is to compare islet isolation outcomes of pancreas preserved by the 2 different preservation methods, and to assess which method is superior. In comparison to UW alone, TLM alone produced a significantly higher islet yield.

TLM alone also yielded higher proportion of transplantable preparations. Islet viability, purity and function did not differ.

Qin H, Matsumoto S, Klintmalm GB, De Vol EB. A meta-analysis for comparison of the two-layer and university of Wisconsin pancreas preservation methods in islet transplantation. Cell Transplant. 2011;20(7):1127-37

DOI: https://dx.doi.org/10.3727/096368910X544942

The meta-analysis aimed to compared the human islet yield and viability of the two-layer method (TLM) and the University of Wisconsin for preserving pancreata. Seventeen studies were included. Use of TLM significantly increased islet yield and viability. The beneficial effects of TLM on islet yield were more evident when TLM was used following UW storage or when prolonged CIT was used. TLM used alone, shorter CIT, and no chemical use all resulted in similar islet viability between TLM and UW groups.

Agrawal A, Gurusamy K, Powis S, Gray DW, Fuller B, Davidson BR. A meta-analysis of the impact of the two-layer method of preservation on human pancreatic islet transplantation. Cell Transplant. 2008;17(12):1315-22.

DOI: https://doi.org/10.3727/096368908787648065

The review evaluated the effectiveness of perfluorocarbons used in the two-layer method (TLM) of pancreas preservation for human islet transplantation. 11 human studies were eligible for the meta-analysis. When comparing TLM with preservation in University of Wisconsin (UW) solution, there was a statistically significant higher islet yield in the TLM group. The proportion of transplantable preparations obtained was not significantly different between the two groups. The rate of successful islet isolations for marginal organs was higher in the TLM group.

Observational studies

Nano R, Kerr-Conte JA, Scholz H, Engelse M, et al. Heterogeneity of Human Pancreatic Islet Isolation Around Europe: Results of a Survey Study. Transplantation. 2020 Jan;104(1):190-196.

DOI: https://dx.doi.org/10.1097/TP.0000000000002777

The questionnaire aims to identify differences in the human pancreatic islet isolation processes within European countries. Eleven islet isolation facilities completed the questionnaire. Differences among facilities emerged in donor selection (age, cold ischemia time, intensive care unit length, amylase concentration), pancreas procurement, isolation procedures (brand and concentration of collagenase, additive, maximum acceptable

digestion time), quality evaluation, and release criteria for transplantation (glucose-stimulated insulin secretion tests, islet numbers, and purity).

Other observational studies are summarised in the evidence table on page 14.

Review articles

Balfoussia, D., et al. (2012). "Advances in machine perfusion graft viability assessment in kidney, liver, pancreas, lung, and heart transplant." Experimental & Clinical Transplantation: Official Journal of the Middle East Society for Organ Transplantation 10(2): 87-100.

Solid organ transplant constitutes the definitive treatment for end-stage organ failure. Better organ preservation methods have enabled use of marginal grafts, thereby expanding the donor pool to meet the growing demand for organs. Static cold storage as a preservation method has been superseded largely by machine perfusion in kidney transplant, with work regarding its use in other organ transplants ongoing. We hope that machine perfusion will allow better graft preservation, and pretransplant assessment, and optimization. The most extensive laboratory, preclinical, and clinical research into machine perfusion organ preservation has focused on kidneys. Successful outcomes in its use in renal transplant have sparked interest for its development and application to the liver, pancreas, heart, and lungs. This article reviews the current state of machine perfusion in abdominal and thoracic organ transplant, focusing on the recent developments in assessing graft viability.

Barlow, A. D., et al. (2013). "Current state of pancreas preservation and implications for DCD pancreas transplantation." Transplantation 95(12): 1419-1424.

One of the main factors limiting potential uptake of pancreas transplantation, particularly in the United Kingdom, is the shortage of grafts. There has therefore been a recent expansion, particularly in the United Kingdom, in the utilization of grafts from donation after cardiac death (DCD) donors. These grafts are subjected to a greater ischemic insult and are arguably at higher risk of poor functional outcome. Although conventional preservation techniques may be adequate for donation after brain death (DBD) and low-risk DCD pancreases, as the number of DCD pancreas transplants increase and the threshold for rejecting organs decreases, the importance of optimal preservation techniques is going to increase. Over recent years, there have been significant advances in preservation techniques for DCD kidneys, improving the outcome of these marginal grafts. However, the use of such techniques for pancreas preservation is extremely limited and mainly historical. This overview describes the background and results of the established method of pancreas preservation for DBD, namely, cold static storage, and describes the use of the two-layer

method. It also reviews pulsatile machine perfusion and normothermic perfusion for pancreas preservation techniques, which have shown promise in the preservation of DCD kidney grafts. The use of these techniques in pancreas preservation is predominantly historical but warrants reevaluation as to the feasibility of applying these techniques to DCD pancreas grafts not only for preservation but also for viability assessment. Further areas for development of pancreas preservation are discussed.

Bellini, M. I., et al. (2019). "The Effect of Preservation Temperature on Liver, Kidney, and Pancreas Tissue ATP in Animal and Preclinical Human Models." J Clin Med 8(9).

The recent advances in machine perfusion (MP) technology involve settings ranging between hypothermic, subnormothermic, and normothermic temperatures. Tissue level adenosine triphosphate (ATP) is a long-established marker of viability and functionality and is universal for all organs. In the midst of a growing number of complex clinical parameters for the quality assessment of graft prior to transplantation, a revisit of ATP may shed light on the underlying reconditioning mechanisms of different perfusion temperatures in the form of restoration of metabolic and energy status. This article aims to review and critically analyse animal and preclinical human studies (discarded grafts) during MP of three abdominal organs (liver, kidney, and pancreas) in which ATP was a primary endpoint. A selective review of recent novel reconditioning approaches relevant to mitigation of graft ischaemia-reperfusion injury via MP and for different perfusion temperatures was also conducted. With a current reiterated interest for oxygenation during MP, a re-introduction of tissue ATP levels may be valuable for graft viability assessment prior to transplantation. Further studies may help delineate the benefits of selective perfusion temperatures on organs viability.

Branchereau, J., et al. (2020). "Pancreas preservation: clinical practice and future developments." Current Opinion in Organ Transplantation 25(4): 329-335.

PURPOSE OF REVIEW: To summarize recently published studies of preservation strategies including machine perfusion in pancreas transplantation.
RECENT FINDINGS: The shortage of conventional donors is leading units to use extended criteria donors (ECDs) and donors after cardiac death (DCD). Static cold storage (SCS) is still the standard method of preservation for pancreases and University of Wisconsin remains the gold standard preservation solution. In experimental studies, oxygen delivered during preservation reduced tissue injury and improved islet cell yield and function. Hypothermic machine perfusion of discarded human pancreases has been shown to improve adenosine triphosphate levels without adversely effect histology and oedema compared with SCS. Normothermic machine perfusion of discarded human organs has so far been challenging and led to increasing injury, rather than preservation. There are currently no clinical studies in pancreas transplant with the exception of a small number of pancreases being transplanted following normothermic regional perfusion.

SUMMARY: The storm of new organ preservation methods is now being more widely studied in the pancreas, with some promising results. These new strategies have the potential to allow expansion of the donor pool and greater utilization of ECD and DCD organs.

Branchereau, J., et al. (2022). "Novel Organ Perfusion and Preservation Strategies in Controlled Donation After Circulatory Death in Pancreas and Kidney Transplantation." Transplantation Proceedings 54(1): 77-79.

BACKGROUND: Kidney and pancreatic transplants from controlled donation after circulatory death donors are vulnerable to ischemia-reperfusion injuries. In this context of transplant shortage, there is a need to optimize the function of these transplants and to develop novel perfusion and preservation strategies in controlled donation after circulatory death in kidney and pancreatic transplants.
IN SITU PERFUSION AND PRESERVATION STRATEGIES: In situ regional normothermic perfusion improves the outcome of kidney transplants from controlled donation after circulatory death and provides equivalent results for the kidney from brain-dead donors. In situ regional normothermic perfusion is under investigation for pancreatic transplants.
EX SITU PERFUSION AND PRESERVATION STRATEGIES: Perfusion on hypothermic machine perfusion is highly recommended for the kidney from controlled donation after cardiac death. Hypothermic oxygenated perfusion machine decreases the rate of graft rejection and graft failure in kidney transplantation. Ex situ normothermic perfusion is an easy way to assess renal function. In the future, kidney transplants could benefit from drug therapy during ex situ normothermic perfusion. In pancreas transplantation, hypothermic machine perfusion and ex situ normothermic perfusion present encouraging results in preclinical studies.

Dholakia, S., et al. (2018). "Preserving and perfusing the allograft pancreas: Past, present, and future." Transplant Rev (Orlando) 32(3): 127-131.

The concept of organ preservation by perfusion dates back to the mid-19th century. Innovations since then have included temperature regulation, perfusion fluid composition and various pumping systems. Advances made in liver, heart and kidney machine preservation are now contributing to increased graft utilisation, assessment of graft viability and potentially improved graft survival. Pancreas transplantation has not benefitted to the same extent from the application of perfusion technology, although the need is just as great. This overview reviews current pancreas specific preservation techniques. We explore concepts, which include static cold storage, use of preservation solutions, the 'two-layer method', and machine perfusion. We also discuss ideas for future development. Narrative review of literature from inception to December 2017 using OVID interfaces searching EMBASE, Google Scholar, and MEDLINE databases. All studies relevant to pancreas perfusion and preservation were examined for clinical relevance with no exclusion criteria. Conference papers and presentations were also reviewed and included where appropriate. The application of recent advances in understanding in ischaemia-reperfusion as well as

technical developments in machine preservation Ischaemia-reperfusion have the potential to improve organ utilisation, viability and outcome.

Fujino, Y. (2010). "Two-layer cold storage method for pancreas and islet cell transplantation." World Journal of Gastroenterology 16(26): 3235-3238.

The two-layer cold storage method (TLM) was first reported in 1988, consisting of a perfluorochemical (PFC) and initially Euro-Collins' solution, which was later replaced by University of Wisconsin solution (UW). PFC is a biologically inert liquid and acts as an oxygen-supplying agent. A pancreas preserved using the TLM is oxygenated through the PFC and substrates are supplied by the UW solution. This allows the pancreas preserved using the TLM to generate adenosine triphosphate during storage, prolonging the preservation time. In a canine model, the TLM was shown to repair and resuscitate warm ischemically damaged pancreata during preservation, improve pancreas graft survival after transplantation, and also improve the islet yield after isolation. Clinical trials using the TLM in pancreas preservation before whole-pancreas transplantation and islet isolation have shown promising outcomes. We describe the role of the TLM in pancreas and islet transplantation.

Hamaoui, K. and V. Papalois (2019). "Machine Perfusion and the Pancreas: Will It Increase the Donor Pool?" Current Diabetes Reports 19(8): 56.

PURPOSE OF REVIEW: Pancreas transplantation enables complete patient independence from exogenous insulin administration and increases both patient survival and quality of life. Despite this, there has been a decline in pancreas transplantation for the past 20 years, influenced by changing donor demographics with more high-risk extended criteria (ECD) and donation after cardiac death (DCD) donors. This review discusses whether the advent of machine perfusion (MP), if extended to the pancreas, can increase the pool of suitable donor organs.

RECENT FINDINGS: Hypothermic and normothermic MP, as forms of preservation deemed superior to cold storage for high-risk kidney and liver donor organs, have opened the avenue for translation of this work into the pancreas. Recent experimental models of porcine and human ex-vivo pancreatic MP are promising. Applications of MP to the pancreas however need refinement-focusing on perfusion protocols and viability assessment tools. Emerging research shows pancreatic MP can potentially offer superior preservation capacity, the ability to both resuscitate and manipulate organs, and assess functional and metabolic organ viability. The future of MP will lie in organ assessment and resuscitation after retrieval, where ultimately organs initially considered high risk and unsuitable for transplantation will be optimised and transformed, making them then available for clinical use, thus increasing the pool of suitably viable pancreata for transplantation.

Min, C. G. and K. K. Papas (2018). "Recent developments in persufflation for organ preservation." Current Opinion in Organ Transplantation 23(3): 330-335.

PURPOSE OF REVIEW: To summarize current literature and recent findings on the potential of humidified oxygenated gas perfusion (persufflation) as an alternative method for improved organ preservation. RECENT FINDINGS: Although there are some conflicting data, the majority of the evidence suggests that persufflation, by enhancing oxygenation, can improve preservation and even rescue organs, including organs with prior exposure to warm ischemia. In some cases, persufflation produced better results than hypothermic machine perfusion. The timing of persufflation is of importance; benefits of persufflation appear to increase as the timing of its administration postprocurement decreases. This may be particularly true for tissues that are more sensitive to ischemia, such as the pancreas prior to islet isolation. Combining oxygen persufflation with nitric oxide and addition of pulsatile flow may provide further benefits and amplify its effects on improving transplant outcomes. SUMMARY: Persufflation is a promising, relatively simple, preservation technique that enables improved oxygenation, which provides protection and improvement in the graft condition during preservation and prior to transplantation. More detailed studies are needed to optimize persufflation and evaluate its short and long-term effects in vivo.

Nicholson, M. L. and S. A. Hosgood (2017). "Organ retrieval and preservation." Surgery (United Kingdom) 35(7): 346-352.

The success of organ transplantation can be attributed to many factors but ultimately depends upon retrieval and preservation techniques to maintain the quality of an organ. Thoracic and abdominal organs from deceased donors are retrieved during a multi-organ procedure. The organs are flushed and cooled in-situ with preservation solution followed by dissection and removal. With the high risk donors such as donation after cardiac death, rapid in-situ cooling is essential to minimize the injury. Live kidney donation is becoming increasingly popular and the disincentives have been significantly reduced by the introduction of minimally invasive techniques such as the laparoscopic retrieval technique. Segments of the liver, pancreas, lung and small bowel are also being used for live donation. Organ preservation traditionally relies on hypothermic temperatures to reduce the metabolism and requirement for oxygen. Storing the organ on ice is the most practiced method of preservation (static cold storage), although other methods such as hypothermic machine perfusion are becoming popular for kidney preservation. Normothermic techniques are also being adopted into clinical practice to improve the quality of marginal organs. This review focuses on retrieval and preservation techniques in solid organ transplantation. Copyright © 2017

Prudhomme, T., et al. (2021). "Ischemia-Reperfusion Injuries Assessment during Pancreas Preservation." International Journal of Molecular Sciences 22(10): 13.

Maintaining organ viability between donation and transplantation is of critical importance for optimal graft function and survival. To date in pancreas transplantation, static cold storage (SCS) is the most widely practiced method of organ preservation. The first

experiments in ex vivo perfusion of the pancreas were performed at the beginning of the 20th century. These perfusions led to organ oedema, hemorrhage, and venous congestion after revascularization. Despite these early hurdles, a number of factors now favor the use of perfusion during preservation: the encouraging results of HMP in kidney transplantation, the development of new perfusion solutions, and the development of organ perfusion machines for the lung, heart, kidneys and liver. This has led to a resurgence of research in machine perfusion for whole organ pancreas preservation. This review highlights the ischemia-reperfusion injuries assessment during ex vivo pancreas perfusion, both for assessment in pre-clinical experimental models as well for future use in the clinic. We evaluated perfusion dynamics, oedema assessment, especially by impedance analysis and MRI, whole organ oxygen consumption, tissue oxygen tension, metabolite concentrations in tissue and perfusate, mitochondrial respiration, cell death, especially by histology, total cell free DNA, caspase activation, and exocrine and endocrine assessment.

Squifflet, J. P., et al. (2011). "Pancreas preservation for pancreas and islet transplantation: a minireview." Transplantation Proceedings 43(9): 3398-3401.

Pancreas preservation by cold storage using University of Wisconsin solution was the mainstay method used for pancreas transplantation during the past 2 decades. Other solutions, such as HTK, Celsior, and SCOT 15, could not demonstrate any advantage for short preservation periods. But the advent of clinical islet transplantation and the larger use of controlled non-heart-beating donors have prompted the transplantation community to develop methods for increasing pancreas graft quality while preventing ischemic reperfusion damages. Oxygenation by 1- or 2-layer methods during pancreas preservation, as well as the use of perfluorocarbons, might increase the islet yield. Based on the former methods, there is a renewed interest in machine perfusion and oxygenation in pancreas preservation for pancreas transplantation and islet preparation.

Taylor, M. J. and S. C. Baicu (2010). "Current state of hypothermic machine perfusion preservation of organs: The clinical perspective." Cryobiology 60(3 Suppl): S20-35.

This review focuses on the application of hypothermic perfusion technology as a topic of current interest with the potential to have a salutary impact on the mounting clinical challenges to improve the quantity and quality of donor organs and the outcome of transplantation. The ex vivo perfusion of donor organs on a machine prior to transplant, as opposed to static cold storage on ice, is not a new idea but is being re-visited because of the prospects of making available more and better organs for transplantation. The rationale for pursuing perfusion technology will be discussed in relation to emerging data on clinical outcomes and economic benefits for kidney transplantation. Reference will also be made to on-going research using other organs with special emphasis on the pancreas for both segmental pancreas and isolated islet transplantation. Anticipated and emerging benefits of hypothermic machine perfusion of organs are: (i) maintaining the patency of the vascular bed, (ii) providing nutrients and low demand oxygen to support reduced energy demands, (iii) removal of metabolic by-products and toxins, (iv) provision of access for administration

of cytoprotective agents and/or immunomodulatory drugs, (v) increase of available assays for organ viability assessment and tissue matching, (vi) facilitation of a change from emergency to elective scheduled surgery with reduced costs and improved outcomes, (vii) improved clinical outcomes as demonstrated by reduced PNF and DGF parameters, (viii) improved stabilization or rescue of ECD kidneys or organs from NHBD that increase the size of the donor pool, (ix) significant economic benefit for the transplant centers and reduced health care costs, and (x) provision of a technology for ex vivo use of non-transplanted human organs for pharmaceutical development research.

Weissenbacher, A., et al. (2019). "The future of organ perfusion and re-conditioning." Transplant International 32(6): 586-597.

Organ preservation and re-conditioning using machine perfusion technologies continue to generate promising results in terms of viability assessment, organ utilization and improved initial graft function. Here, we summarize the latest findings and study the results of ex-vivo/ex-situ hypothermic (HMP) and normothermic machine perfusion (NMP) in the area of abdominal organ transplantation (kidney, liver, pancreas and intestine). We also consider the potential role of normothermic regional perfusion (NRP) to re-condition donors after circulatory death organs before retrieval. The findings from clinical studies reported to date suggest that machine perfusion will offer real benefits when compared with conventional cold preservation. Several randomized trials are expected to report their findings within the next 2 years which may shed light on the relative merits of different perfusion methods and could indicate which perfusion parameters may be most useful to predict organ quality and viability. Further work is needed to identify composite endpoints that are relevant for transplanted organs that have undergone machine preservation. Multi-centre trials to compare and analyse the combinations of NRP followed by HMP and/or NMP, either directly after organ retrieval using transportable devices or when back-to-base, are needed. The potential applications of machine preservation technology beyond the field of solid organ transplantation are also considered.

Author (date)	Population	Type of study	Intervention
**Hypothermic machine perfusion studies**			
Ogbemudia 2021 (1) https://doi.org10.1111/tri.13990	Porcine pancreas	Comparative groups	6h oxygenated HMP versus SCS
Prudhomme 2021 (2) http://dx.doi.org/10.1111/tri.13797	Porcine pancreas	Animal study, cohort	24h HMP vs 24h SCS
Doppenberg 2021 (3) https://dx.doi.org/10.1111/tri.13927	Discarded human pancreas	Cohort study	6h oxygenated HMP versus SCS
Prudhomme 2020 (4) https://dx.doi.org/10.1111/aor.13655	Primate pancreas	Animal study, cohort	24h HMP (at 3 different pressures) vs static cold storage
Branchereau 2018 (5) https://dx.doi.org/10.1016/j.cryobiol.2018.10.002	Discarded human pancreas	Cohort study	24h hypothermic pulsatile perfusion (split and whole organ) vs SCS
Hamaoui 2018 (6) https://dx.doi.org/10.1016/j.jss.2017.11.052	Porcine and human pancreas	Animal study, cohort Human pancreata were used in the development of a preclinical model.	24h CS + viability assessment on NRP (n=3) versus 24h CS + 5h HMP + viability assessment on NRP (n=3).

Author (date)	Population	Type of study	Intervention
Leemkuil 2018 (7)<br>https://dx.doi.org/10.1097/TXD.0000000000000829	Discarded human pancreas	Cohort	6h oxygenated hypothermic MP (n=10) vs static cold storage (n=10)
Fu 2014 (8)<br>** no access to full text article	Pig pancreas	Animal study, cohort	MP (temperature not specified for 120, 180 and 240 mins) vs SCS
Reddy 2014 (9)<br>https://dx.doi.org/10.1111/tri.12313	Rat pancreas	Animal study, cohort	6h HMP vs 6h persufflation vs SCS
Hanf 2012 (10)<br>http://dx.doi.org/10.1111/j.1600-6143.2011.03983.x	Human pancreas	Cohort study	HMP (uDCD) vs SCS (ECD and SPK donors)
Weegman 2012 (11)<br>https://pubmed.ncbi.nlm.nih.gov/24083059/	Porcine pancreas/islet	Each pancreas acted as its own control as the model was a "split lobe" model.	Twenty-four hours of HMP of pancreata followed by islet isolation.
Taylor 2010 (12)<br>https://dx.doi.org/10.3727/096368910X486316	Porcine pancreas	Animal study, cohort	HMP (n=14) vs SCS (n=9)
Karcz 2010 (13)<br>https://pubmed.ncbi.nlm.nih.gov/20199372/	Porcine pancreas	Animal study, case series	HMP (315 mins)

Author (date)	Population	Type of study	Intervention
Taylor 2008 (14) http://dx.doi.org/10.1016/j.transproceed.2008.01.004	Porcine pancreas	Animal study, cohort	24h HMP (n=7) vs SCS (n=9)
Leeser 2004 (15) https://pubmed.ncbi.nlm.nih.gov/15194365/	Human pancreas/islet	Single group, case series	SCS (for mean of 13h) followed by 4h machine pulsatile perfusion vs nonpumped islet isolations

**Normothermic machine perfusion studies**			
Mazilescu 2022 (16) https://dx.doi.org/10.1111/ajt.17019	Porcine pancreas	Animal study, case series	6h Normothermic ex situ pancreas perfusion (NESPP)
Richards 2021 (17) https://dx.doi.org/10.1093/bjs/znab212	Human pancreas	Case series	2h normothermic regional perfusion (DCD) versus SCS (DBD)
Kumar 2018 (18) https://dx.doi.org/10.1016/j.ijsu.2018.04.057	Porcine pancreas	Animal study, cohort	4h ex-vivo perfusion at 50mmHg (n=9) vs 20mmHg (n=4)
Nassar 2018 (19) https://doi.org/10.1111/aor.12985	Discarded human pancreas	Case reports	6h (n=2) or 12h (n=1) normothermic ex-vivo perfusion
Kuan 2017 (20) https://doi.org/10.1111/aor.12770	Porcine pancreas (+/- kidney)	Animal study	2h normothermic hemoperfusion (with and without addition of the kidney as a dialysis organ)

Miñambres 2017 (21) http://dx.doi.org/10.1111/ajt.14214	Human pancreas	Case report	1h normothermic regional perfusion (n=1)
Barlow 2015 (22) https://dx.doi.org/10.1111/ajt.13303	Discarded human pancreas	Single group, case series	1-2h ex-vivo normothermic perfusion (n=5)
Oniscu 2014 (23) https://dx.doi.org/10.1111/ajt.12927	Human pancreas	Case series	2h normothermic regional perfusion (n=5, including 2 for SPK and 2 for islet isolation)
Butler 2014 (24) (see also Oniscu 2014) https://dx.doi.org/10.1097/TP.0000000000000082	Human pancreas	Case series	2h normothermic regional perfusion (n=5, including 2 for SPK and 2 for islet isolation)
Magliocca 2005 (25)	Human pancreas	Case series	Normothermic ECMO (DCD) vs SCS (DBD)

Author (date)	Population	Type of study	Intervention
**Persufflation study**			
Kelly 2019 (26) https://doi.org/10.1097/tp.0000000000002400	Human islet preparations	Cohort study. Treatment groups were not randomized. SCS+PSF pancreata were collected from institutions capable of organ persufflation.	SCS followed by persufflation (n=13) vs SCS (n=11)

**Two layer method studies**			
Goto 2014 (27)	Rat pancreas/islet	Animal study, cohort (different warm ischemia times)	3h TLM
Aldibbiat 2012 (28) https://dx.doi.org/10.3727/215517911X617905	Human pancreas/islets	Cohort study	TLM vs SCS
Gioviale 2011 (29) https://dx.doi.org/10.5301/IJAO.2011.8465	Porcine pancreas/islet	Animal study, cohort	4h TLM (n=10) vs SCS (n=10)

Scott 2010 (30) https://dx.doi.org/10.1016/j.transproceed.2010.05.092	Porcine pancreas	Animal study, cohort	24h persufflation vs 24h TLM
Goto 2007 (37)	Rat pancreas/islet	Animal study, cohort	TLM + preoxygenated perfluorocarbon vs TLM
Caballero-Corbalan 2007 (38)	Human pancreas/islet	Cohort study	TLM vs SCS (UW)
Noguchi 2006 (33) **	Porcine pancreas/islet (slaughterhouse)	Animal study, cohort	TLM (UW) vs TLM (M-Kyoto)
Takahashi 2006 (39)	Rat isolated islets	Animal study, cohort	Modified TLM 37 degrees vs modified TLM 4 degrees vs SCS vs TLM
Salehi 2006 (40)	Human pancreas/islet	Cohort study	Part A: TLM (n=3) vs SCS (n=3)<br>Part B: 2-4h TLM after 7-11h SCS (n=32) or SCS (n=33)
Kin 2006 (41)	Human pancreas/islet	Cohort study	TLM (n=91) vs SCS (n=91)
Tanaka 2005 (42)	Rat pancreas/islet	Animal study, cohort	3h TLM vs no preservation
Brandhorst 2005 (43)	Porcine pancreas/islet	Animal study, cohort	TLM preloaded (distended with enzymes) vs TLM postloaded vs SCS
Kakinoki 2005 (44)	Rat pancreas/islet	Animal study, cohort	TLM vs SCS

Tsujimura 2004 (45)	Human pancreas/islet	Cohort study	TLM (TLM for 3.2 +/- 0.5h after 11.1 +/- 0.9h of cold storage in UW) vs SCS
Tsujimura 2004 (46)	Human pancreas/islet	Cohort study	TLM (n=30) vs SCS (n=112)
Matsumoto 2004 (31)	Human pancreas/islets	Cohort study	18h TLM (n=3) vs 12h SCS (n=6)
Matsumoto 2003 (32)	Human (marginal) and primate pancreas/islet	Cohort study	Primate model: TLM (n=5) vs SCS (n=5)<br>Human model: TLM (n=8) vs SCS (n=8)
Matsuda 2003 (47)	Rat pancreas/islet	Animal study, cohort	TLM vs SCS
Matsumoto 2002 (35)	Human pancreas/islet	RCT	Short TLM vs long TLM vs 12h SCS vs 24h SCS
Matsumoto 2002 (36) **	Human and primate pancreas/islet	Cohort study	TLM vs SCS<br>Primate model: pancreata were preserved by the static TLM, the original TLM, and UW for 5 h prior to islet isolation.<br>Human model: pancreata were preserved with the static TLM or the original TLM or UW for 4–13h
Tsujimura 2002 (48)	Human pancreas/islet	Cohort study	TLM after SCS (n=7) versus SCS (n=14)

Tanioka 1997 (49)	Canine pancreas/islet	Animal study, cohort	3h TLM vs 24h TLM vs 3h SCS vs 24h SCS vs no preservation
No access to full text:<br>Kneteman 1990 (50)			

** These studies are summarised by Matsumoto 2009 (34) https://doi.org/10.1177/096368970901805-610

References

1. Ogbemudia AE, Hakim G, Dengu F, El-Gilani F, Dumbill R, Mulvey J, et al. Development of ex situ normothermic reperfusion as an innovative method to assess pancreases after preservation. Transpl Int. 2021;34(9):1630-42.
2. Prudhomme T, Kervella D, Ogbemudia AE, Gauttier V, Le Bas-Bernardet S, Minault D, et al. Successful pancreas allotransplantations after hypothermic machine perfusion in a novel diabetic porcine model: a controlled study. Transplant International. 2021;34(2):353-64.
3. Doppenberg JB, Leemkuil M, Engelse MA, Krikke C, de Koning EJP, Leuvenink HGD. Hypothermic oxygenated machine perfusion of the human pancreas for clinical islet isolation: a prospective feasibility study. Transplant International. 2021;34(8):1397-407.
4. Prudhomme T, Renaudin K, Lo Faro ML, Cantarovich D, Kervella D, Minault D, et al. Ex situ hypothermic perfusion of nonhuman primate pancreas: A feasibility study. Artificial Organs. 2020;44(7):736-43.
5. Branchereau J, Renaudin K, Kervella D, Bernadet S, Karam G, Blancho G, et al. Hypothermic pulsatile perfusion of human pancreas: Preliminary technical feasibility study based on histology. Cryobiology. 2018;85:56-62.
6. Hamaoui K, Gowers S, Sandhu B, Vallant N, Cook T, Boutelle M, et al. Development of pancreatic machine perfusion: translational steps from porcine to human models. Journal of Surgical Research. 2018;223:263-74.
7. Leemkuil M, Lier G, Engelse MA, Ploeg RJ, de Koning EJP, t Hart NA, et al. Hypothermic Oxygenated Machine Perfusion of the Human Donor Pancreas. Transplant Direct. 2018;4(10):e388.
8. Fu D, Liu H, Cheng Y, Song W, Wang Y, Liu Y. Preservative effect evaluation of machine perfusion on pig DCD pancreas. Experimental and Clinical Cardiology. 2014;20(1):2151-61.
9. Reddy MS, Carter N, Cunningham A, Shaw J, Talbot D. Portal Venous Oxygen Persufflation of the Donation after Cardiac Death pancreas in a rat model is superior to static cold storage and hypothermic machine perfusion. Transplant International. 2014;27(6):634-9.
10. Hanf W, Codas R, Meas-Yedid V, Berthiller J, Buron F, Chauvet C, et al. Kidney graft outcome and quality (after transplantation) from uncontrolled deceased donors after cardiac arrest. American Journal of Transplantation. 2012;12(6):1541-50.

11. Weegman BP, Taylor MJ, Baicu SC, Scott WE, 3rd, Mueller KR, Kitzmann JD, et al. Hypothermic Perfusion Preservation of Pancreas for Islet Grafts: Validation Using a Split Lobe Porcine Model. Cell med. 2012;2(3):105-10.
12. Taylor MJ, Baicu S, Greene E, Vazquez A, Brassil J. Islet isolation from juvenile porcine pancreas after 24-h hypothermic machine perfusion preservation. Cell Transplantation. 2010;19(5):613-28.
13. Karcz M, Cook HT, Sibbons P, Gray C, Dorling A, Papalois V. An ex-vivo model for hypothermic pulsatile perfusion of porcine pancreata: hemodynamic and morphologic characteristics. Exp Clin Transplant. 2010;8(1):55-60.
14. Taylor MJ, Baicu S, Leman B, Greene E, Vazquez A, Brassil J. Twenty-Four Hour Hypothermic Machine Perfusion Preservation of Porcine Pancreas Facilitates Processing for Islet Isolation. Transplantation Proceedings. 2008;40(2):480-2.
15. Leeser DB, Bingaman AW, Poliakova L, Shi Q, Gage F, Bartlett ST, et al. Pulsatile pump perfusion of pancreata before human islet cell isolation. Transplantation Proceedings. 2004;36(4):1050-1.
16. Mazilescu LI, Parmentier C, Kalimuthu SN, Ganesh S, Kawamura M, Goto T, et al. Normothermic ex situ pancreas perfusion for the preservation of porcine pancreas grafts. American Journal of Transplantation. 2022;22(5):1339-49.
17. Richards JA, Roberts JL, Fedotovs A, Paul S, Cottee S, Defries G, et al. Outcomes for circulatory death and brainstem death pancreas transplantation with or without use of normothermic regional perfusion. British Journal of Surgery. 2021;108(12):1406-8.
18. Kumar R, Chung WY, Runau F, Isherwood JD, Kuan KG, West K, et al. Ex vivo normothermic porcine pancreas: A physiological model for preservation and transplant study. International Journal Of Surgery. 2018;54(Pt A):206-15.
19. Nassar A, Liu Q, Walsh M, Quintini C. Normothermic Ex Vivo Perfusion of Discarded Human Pancreas. Artif Organs. 2018;42(3):334-5.
20. Kuan KG, Wee MN, Chung WY, Kumar R, Mees ST, Dennison A, et al. A Study of Normothermic Hemoperfusion of the Porcine Pancreas and Kidney. Artif Organs. 2017;41(5):490-5.
21. Minambres E, Suberviola B, Dominguez-Gil B, Rodrigo E, Ruiz-San Millan JC, Rodriguez-San Juan JC, et al. Improving the Outcomes of Organs Obtained From Controlled Donation After Circulatory Death Donors Using Abdominal Normothermic Regional Perfusion. American Journal of Transplantation. 2017;17(8):2165-72.
22. Barlow AD, Hamed MO, Mallon DH, Brais RJ, Gribble FM, Scott MA, et al. Use of Ex Vivo Normothermic Perfusion for Quality Assessment of Discarded Human Donor Pancreases. American Journal of Transplantation. 2015;15(9):2475-82.
23. Oniscu GC, Randle LV, Muiesan P, Butler AJ, Currie IS, Perera MT, et al. In situ normothermic regional perfusion for controlled donation after circulatory death--the United Kingdom experience. American Journal of Transplantation. 2014;14(12):2846-54.
24. Butler AJ, Randle LV, Watson CJ. Normothermic regional perfusion for donation after circulatory death without prior heparinization. Transplantation. 2014;97(12):1272-8.
25. Magliocca JF, Magee JC, Rowe SA, Gravel MT, Chenault RH, 2nd, Merion RM, et al. Extracorporeal support for organ donation after cardiac death effectively expands the donor pool. J Trauma. 2005;58(6):1095-101; discussion 101-2.

26. Kelly AC, Smith KE, Purvis WG, Min CG, Weber CS, Cooksey AM, et al. Oxygen Perfusion (Persufflation) of Human Pancreata Enhances Insulin Secretion and Attenuates Islet Proinflammatory Signaling. Transplantation. 2019;103(1):160-7.
27. Goto T, Fujino Y, Kanashiro M, Tanioka Y, Sakai T, Yoshikawa T, et al. Two-layer challenge test as an assessment to predict islet graft function. Hepato-Gastroenterology. 2014;61(130):502-6.
28. Aldibbiat A, Huang GC, Zhao M, Holliman GN, Ferguson L, Hughes S, et al. Validation of Islet Transport From a Geographically Distant Isolation Center Enabling Equitable Access and National Health Service Funding of a Clinical Islet Transplant Program for England. Cell med. 2012;2(3):97-104.
29. Gioviale MC, Damiano G, Palumbo VD, Bellavia M, Cacciabaudo F, Cassata G, et al. Pancreatic islets from non-heart-beating donor pig: two-layer preservation method in an in vitro porcine model. International Journal of Artificial Organs. 2011;34(6):519-25.
30. Scott WE, 3rd, O'Brien TD, Ferrer-Fabrega J, Avgoustiniatos ES, Weegman BP, Anazawa T, et al. Persufflation improves pancreas preservation when compared with the two-layer method. Transplantation Proceedings. 2010;42(6):2016-9.
31. Matsumoto S, Zhang G, Qualley S, Clever J, Tombrello Y, Strong DM, et al. The effect of two-layer (University of Wisconsin solution/perfluorochemical) preservation method on clinical grade pancreata prior to islet isolation and transplantation. Transplantation Proceedings. 2004;36(4):1037-9.
32. Matsumoto S, Rigley TH, Reems JA, Kuroda Y, Stevens RB. Improved islet yields from Macaca nemestrina and marginal human pancreata after two-layer method preservation and endogenous trypsin inhibition. American Journal of Transplantation. 2003;3(1):53-63.
33. Noguchi H, Ueda M, Nakai Y, Iwanaga Y, Okitsu T, Nagata H, et al. Modified two-layer preservation method (M-Kyoto/PFC) improves islet yields in islet isolation. American Journal of Transplantation. 2006;6(3):496-504.
34. Matsumoto S, Noguchi H, Hatanaka N, Shimoda M, Kobayashi N, Jackson A, et al. Estimation of donor usability for islet transplantation in the United States with the kyoto islet isolation method. Cell Transplantation. 2009;18(5):549-56.
35. Matsumoto S, Qualley SA, Goel S, Hagman DK, Sweet IR, Poitout V, et al. Effect of the two-layer (University of Wisconsin solution-perfluorochemical plus O2) method of pancreas preservation on human islet isolation, as assessed by the Edmonton Isolation Protocol. Transplantation. 2002;74(10):1414-9.
36. Matsumoto S, Rigley TH, Qualley SA, Kuroda Y, Reems JA, Stevens RB. Efficacy of the oxygen-charged static two-layer method for short-term pancreas preservation and islet isolation from nonhuman primate and human pancreata. Cell Transplantation. 2002;11(8):769-77.
37. Goto T, Tanioka Y, Sakai T, Terai S, Kamoda Y, Li S, et al. Application of the two-layer method on pancreas digestion results in improved islet yield and maintained viability of isolated islets. Transplantation. 2007;83(6):754-8.
38. Caballero-Corbalan J, Eich T, Lundgren T, Foss A, Felldin M, Kallen R, et al. No beneficial effect of two-layer storage compared with UW-storage on human islet isolation and transplantation. Transplantation. 2007;84(7):864-9.

39. Takahashi T, Tanioka Y, Matsuda T, Toyama H, Kakinoki K, Li S, et al. Impact of the two-layer method on the quality of isolated pancreatic islets. Hepato-Gastroenterology. 2006;53(68):179-82.
40. Salehi P, Mirbolooki M, Kin T, Tsujimura T, Shapiro AM, Churchill TA, et al. Ameliorating injury during preservation and isolation of human islets using the two-layer method with perfluorocarbon and UW solution. Cell Transplantation. 2006;15(2):187-94.
41. Kin T, Mirbolooki M, Salehi P, Tsukada M, O'Gorman D, Imes S, et al. Islet isolation and transplantation outcomes of pancreas preserved with University of Wisconsin solution versus two-layer method using preoxygenated perfluorocarbon. Transplantation. 2006;82(10):1286-90.
42. Tanaka T, Suzuki Y, Tanioka Y, Sakai T, Kakinoki K, Goto T, et al. Possibility of islet transplantation from a nonheartbeating donor pancreas resuscitated by the two-layer method. Transplantation. 2005;80(6):738-42.
43. Brandhorst D, Iken M, Tanioka Y, Brendel MD, Bretzel RG, Brandhorst H. Influence of collagenase loading on long-term preservation of pig pancreas by the two-layer method for subsequent islet isolation. Transplantation. 2005;79(1):38-43.
44. Kakinoki K, Fujino Y, Suzuki Y, Li S, Tanioka Y, Sakai T, et al. Superiority of the two-layer method before islet isolation confirmed by in vivo viability assessment. Transplantation. 2005;79(11):1516-21.
45. Tsujimura T, Kuroda Y, Churchill TA, Avila JG, Kin T, Shapiro AM, et al. Short-term storage of the ischemically damaged human pancreas by the two-layer method prior to islet isolation. Cell Transplantation. 2004;13(1):67-73.
46. Tsujimura T, Kuroda Y, Avila JG, Kin T, Oberholzer J, Shapiro AM, et al. Influence of pancreas preservation on human islet isolation outcomes: impact of the two-layer method. Transplantation. 2004;78(1):96-100.
47. Matsuda T, Suzuki Y, Tanioka Y, Toyama H, Kakinoki K, Hiraoka K, et al. Pancreas preservation by the 2-layer cold storage method before islet isolation protects isolated islets against apoptosis through the mitochondrial pathway. Surgery. 2003;134(3):437-45.
48. Tsujimura T, Kuroda Y, Kin T, Avila JG, Rajotte RV, Korbutt GS, et al. Human islet transplantation from pancreases with prolonged cold ischemia using additional preservation by the two-layer (UW solution/perfluorochemical) cold-storage method. Transplantation. 2002;74(12):1687-91.
49. Tanioka Y, Sutherland DE, Kuroda Y, Gilmore TR, Asaheim TC, Kronson JW, et al. Excellence of the two-layer method (University of Wisconsin solution/perfluorochemical) in pancreas preservation before islet isolation. Surgery. 1997;122(2):435-41; discussion 41-2.
50. Kneteman NM, DeGroot TJ, Warnock GL, Rajotte RV. Pancreas preservation prior to islet isolation: evaluation of storage solutions in a rodent model. Transplantation Proceedings. 1990;22(2):541-2.
